# Restoration‐mediated secondary contact leads to introgression of alewife ecotypes separated by a colonial‐era dam

**DOI:** 10.1111/eva.12890

**Published:** 2019-11-18

**Authors:** Kerry Reid, John Carlos Garza, Steven R. Gephard, Adalgisa Caccone, David M. Post, Eric P. Palkovacs

**Affiliations:** ^1^ Department of Ecology and Evolutionary Biology University of California Santa Cruz CA USA; ^2^ Southwest Fisheries Science Center National Marine Fisheries Service Santa Cruz CA USA; ^3^ Department of Ocean Sciences University of California Santa Cruz CA USA; ^4^ Fisheries Division Connecticut Department of Energy and Environmental Protection Old Lyme CT USA; ^5^ Department of Ecology and Evolutionary Biology Yale University New Haven CT USA

**Keywords:** admixture, anthropogenic alteration, dams, genetic swamping, human‐mediated hybridization, management experiment, parentage, river herring

## Abstract

Secondary contact may have important implications for ecological and evolutionary processes; however, few studies have tracked the outcomes of secondary contact from its onset in natural ecosystems. We evaluated an anadromous alewife (*Alosa pseudoharengus* **)** reintroduction project in Rogers Lake (Connecticut, USA), which contains a landlocked alewife population that was isolated as a result of colonial‐era damming. After access to the ocean was restored, adult anadromous alewife were stocked into the lake. We assessed anadromous juvenile production, the magnitude and direction of introgression, and the potential for competition between ecotypes. We obtained fin clips from all adult alewife stocked into the lake during the restoration and a sample of juveniles produced in the lake two years after the stocking began. We assessed the ancestry of juveniles using categorical assignment and pedigree reconstruction with newly developed microhaplotype genetic markers. Anadromous alewives successfully spawned in the lake and hybridized with the landlocked population. Parentage assignments revealed that male and female anadromous fish contributed equally to juvenile F1 hybrids. The presence of landlocked backcrosses shows that some hybrids were produced within the first two years of secondary contact, matured in the lake, and reproduced. Therefore, introgression appears directional, from anadromous into landlocked, in the lake environment. Differences in estimated abundance of juveniles of different ecotypes in different habitats were also detected, which may reduce competition between ecotypes as the restoration continues. Our results illustrate the utility of restoration projects to study the outcomes of secondary contact in real ecosystems.

## 1 INTRODUCTION

Secondary contact occurs when two populations that have been isolated for a period of time reestablish the ability to interact. Assessing the ecological and evolutionary dynamics of secondary contact from its initial onset is essential for understanding how these processes shape the final outcomes (Arnegard et al., 2014). However, it is difficult to monitor such interactions in natural populations. Work that has been able to evaluate some aspects of the initial stages of secondary contact includes introduction experiments (Morissette, Sirois, Lester, Wilson, & Bernatchez, 2018; Veale & Russello, 2016) and genomic analyses of long‐studied systems such as Darwin's finches (Lamichaney et al., 2018).

Fragmentation of habitat by anthropogenic activities may disrupt gene flow and lead to allopatric isolation of previously connected populations, leaving them on independent evolutionary trajectories (Saunders, Hobbs, & Margules, 1991). Subsequent efforts to restore connectivity may lead to secondary contact (Hutchings & Myers, 1985; Jones, Brown, Pemberton, & Braithwaite, 2006; Tulp et al., 2013). Examples of restoration efforts to enhance connectivity include human movement of organisms to encourage gene flow, establishment of migration corridors between fragmented habitats, and the removal of physical barriers to movement (Mech & Hallett, 2001; Tewksbury et al., 2002; Veale & Russello, 2016). The ecological and evolutionary processes at play after the initiation of secondary contact may include (a) competition and competitive exclusion, if resources are limited and one ecotype outcompetes the other (Gurnell, Wauters, Lurz, & Tosi, 2004; Perry, Feder, Dwyer, & Lodge, 2001), (b) coexistence and speciation via reinforcement, if ecotypes fill unique niches within the shared environment (Butlin & Smadja, 2018; Levine & HilleRisLambers, 2009; Mayfield & Levine, 2010), and (c) hybridization, if pre‐ and postzygotic barriers are relatively weak (Garrick et al., 2012; Morissette et al., 2018; Rius & Darling, 2014; Veale & Russello, 2016).

The consequences of hybridization between phenotypically distinct ecotypes coming into secondary contact are dependent on the relative fitness of hybrids, the relative abundance of the distinct ecotypes, and the extent and nature of genetic differentiation between them. Hybridization can increase genetic diversity and lead to the transfer of adaptive alleles between populations (Crispo, Moore, Lee‐Yaw, Gray, & Haller, 2011; Hamilton & Miller, 2016; Hedrick, 2013) but may also lead to the introgression of maladaptive alleles, lowering the fitness of the population targeted for recovery (Araki, Cooper, & Blouin, 2007; Todesco et al., 2016). Continued hybridization and introgression can ultimately lead to the formation of hybrid swarms and the fusion of distinct ecotypes or species, so‐called ‘extinction through hybridization’ (Garrick et al., 2014; Hasselman et al., 2014; Rhymer & Simberloff, 1996; Seehausen, Takimoto, Roy, & Jokela, 2008; Todesco et al., 2016).

In aquatic ecosystems, the construction of dams and other barriers disrupts ecosystem connectivity and can sever gene flow between populations of fish and other species. In addition, dam construction can lead to the isolation of fish populations with the formation of freshwater‐resident ecotypes from anadromous ancestors (Clemento, Anderson, Boughton, Girman, & Garza, 2009; Palkovacs, Dion, Post, & Caccone, 2008; Pearse, Miller, Abadía‐Cardoso, & Garza, 2014). The phenotypic and genetic consequences of such barriers have been well characterized in several species and include reduced genetic diversity, diminished age and size at maturation, reduced fecundity, and altered foraging traits in populations isolated above barriers (Closs, Hicks, & Jellyman, 2013; Franssen, Harris, Clark, Schaefer, & Stewart, 2013; Jones, Palkovacs, & Post, 2013; Palkovacs et al., 2008; Post, Palkovacs, Schielke, & Dodson, 2008). Restoration efforts that remove dams, build fishways to circumvent dams or stock fish above dams, allow access to previously suitable habitat and can lead to secondary contact between populations that have been on independent evolutionary trajectories.

The alewife (*Alosa pseudoharengus*) displays two life history forms or ecotypes—an anadromous form and a freshwater landlocked form. Anadromous alewife migrate up streams and rivers in the spring to spawn in lakes, where the juveniles rear for several months, before making their seaward out‐migration in the late summer and autumn (Loesch, 1987). Landlocked populations of alewife complete their entire life cycle in freshwater lakes. The construction of colonial‐era dams led to the isolation of alewife populations in several coastal lakes in Connecticut, USA (Palkovacs et al., 2008). These populations show parallel shifts in phenotype, including life history (migratory behavior, fecundity, generation time, spawning time), morphology (body size, gape width, gill‐raker spacing), prey selectivity, salinity tolerance, and osmoregulation (Jones et al., 2013; Palkovacs et al., 2008; Palkovacs, Mandeville, & Post, 2014; Velotta et al., 2017). Changes in some traits, such as salinity tolerance and osmoregulation, are the direct result of residency in freshwater (Velotta et al., 2017). In contrast, changes in foraging traits are likely the result of eco‐evolutionary feedbacks driven by the strong effects of alewives themselves on lake zooplankton communities (Palkovacs & Post, 2008, 2009; Post et al., 2008). Differences in migratory behavior and foraging traits create a cascade of ecological and evolutionary changes that propagate through lake food webs, shaping the ecology and evolution of alewife prey (e.g., *Daphnia ambigua*; Walsh, Delong, Hanley, & Post, 2012; Walsh & Post, 2012), competitors (e.g., juvenile largemouth bass and bluegill sunfish; Boel, Brodersen, Baktoft, Koed, & Post, 2018; Huss, Howeth, Osterman, & Post, 2014), and predators (e.g., chain pickerel; Broderson, Howeth, & Post, 2015).

Range‐wide population genetic studies of anadromous alewife have identified regional genetic groups and an overall pattern of isolation by distance caused by limited gene flow among spawning populations that is dependent on geographic proximity (Palkovacs, Hasselman, et al., 2014; Reid et al., 2018). Landlocked alewife populations show genetic patterns indicative of several independent isolations from downstream anadromous populations due to the construction of dams (Palkovacs et al., 2008).

Anadromous alewife populations have severely declined in recent years, and lack of recovery is likely due to a combination of factors that include barriers such as dams preventing access to suitable spawning habitat and overfishing in the form of bycatch (Atlantic States Marine Fisheries Commission, 2012; Palkovacs, Hasselman, et al., 2014). Conservation and management of alewife has focused on eliminating most harvest in freshwater, limiting bycatch in marine fisheries, and, most recently, removing dams or installing fishways to restore access to historical freshwater spawning habitat (Hasselman et al., 2015; Hasselman & Limburg, 2012). Landlocked alewife populations are found in several of the Connecticut lakes targeted for anadromous alewife restoration through dam removals and fishways (Palkovacs et al., 2008). In lakes where landlocked alewife populations are found, restoration projects will lead to secondary contact between anadromous and landlocked alewife, offering an opportunity to examine the outcome of secondary contact at the scale of whole‐lake ecosystems.

**Figure 1:**
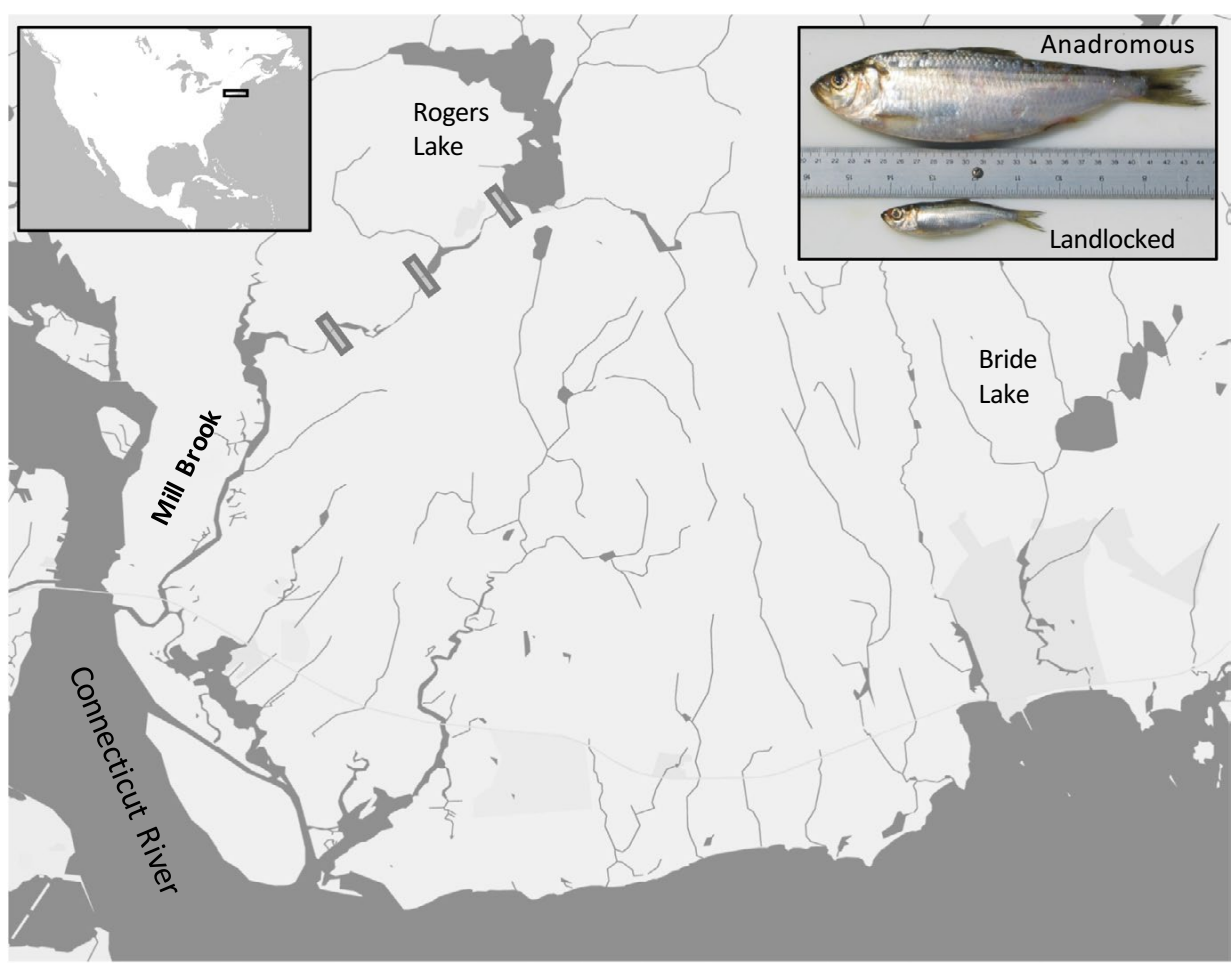
Location of Rogers Lake, with the resident landlocked population, and Mill Brook and Bride Lake from where anadromous adults were stocked. Gray boxes indicate where fishways were built to restore anadromous access to Rogers Lake. Inset image showing the gross morphological differences between adult anadromous (top) and landlocked (bottom) alewife

In this study, we assessed the initial outcome of secondary contact between adults of the anadromous and landlocked alewife ecotypes in Rogers Lake (Old Lyme, Connecticut; Figure 1). Previous research has shown that alewife, and its sister species blueback herring, hybridize when isolated above barriers (Hasselman et al., 2014), indicating that there may be limited postzygotic barriers to reproduction between alewife ecotypes. In addition, a recent study by Littrell et al. (2018) assessed whether differences in spawning time between anadromous and landlocked ecotypes could create a prezygotic barrier that would limit hybridization in secondary contact and found ~3%–13% overlap in the period of spawning time over several years. We therefore hypothesize that hybridization will occur during the initial stages of secondary contact in Rogers Lake, but its extent will be limited by the degree of spawning time overlap between alewife ecotypes.

Access to Rogers Lake was restored for anadromous alewife in 2014 with the completion of the last of three fishways on colonial‐era dams isolating Rogers Lake from the ocean. This allowed anadromous alewife access to Rogers Lake for the first time in over 300 years. To initiate recovery of the anadromous alewife run, anadromous adults were stocked into Rogers Lake starting in 2015. In 2017, we sampled alewife juveniles produced in the lake following this stocking. This restoration project provided a rare opportunity to assess the abundance of anadromous, landlocked, and hybrid offspring produced immediately following secondary contact. To provide sufficient power to distinguish the different ecotypes and hybrid classes, we developed a set of microhaplotype genetic markers, a relatively novel type of marker that has high discriminatory power for both categorical assignment and pedigree reconstruction (Baetscher, Clemento, Ng, Anderson, & Garza, 2018; McKinney, Seeb, & Seeb, 2017). Our study provides insight into the magnitude and direction of introgression and the potential for differences in habitat use to mediate competition between alewife ecotypes during the initial stages of secondary contact.

## 2 METHODS

### 2.1 Study system

Rogers Lake is a 106‐ha, 20‐m‐deep, moderately productive lake in the Connecticut River watershed (Figure 1). It contains a diverse fish community and a population of landlocked alewife. Historically, Rogers Lake provided spawning habitat for anadromous alewife, but this habitat was lost in the 1670s when two low‐head dams were built along Mill Brook isolating Rogers Lake from the ocean (Twining & Post, 2013; Twining, West, & Post, 2013). A third dam was built at the outlet of Rogers Lake in the late 1700s or early 1800s. Molecular genetic data, historical records, and paleolimnological evidence all indicate that the current landlocked population was likely isolated from their anadromous ancestors in the late 1600s, around the time that the first colonial dams were built (Palkovacs et al., 2008; Twining & Post, 2013).

### 2.2 Anadromous stocking and fishway monitoring

Recovery of anadromous fish runs is often initiated by stocking adult fish into the newly accessible habitat. In the spring of 2015, 134 fish collected from Mill Brook were stocked into Rogers Lake, downstream of the first fishway, which was all the alewife that were detected entering Mill Brook that year. Due to the continued low abundance of anadromous alewife in Mill Brook, in 2016 and 2017, adult anadromous alewife from nearby Bride Brook were stocked into Rogers Lake. Bride Brook has a consistently large spawning alewife population and is genetically very similar to the Mill Brook population (*F*_ST_ = 0.013; Reid et al., 2018). Rogers Lake was stocked with 1,144 and 2,787 anadromous adults in 2016 and 2017, respectively.

Anadromous adult alewife were collected and stocked into Rogers Lake on four separate dates in 2015 (36 fish on April 15, 3 fish on April 17, 89 fish on May 1, and 6 fish on May 13), two separate dates in 2016 (576 on May 2 and 568 on May 4), and three separate dates in 2017 (1,004 fish on March 30, 891 fish on April 12, and 892 fish on April 19). For each adult stocked, we measured length and recorded sex, and we collected a small fin clip nonlethally. Fin clips were placed on blotting paper to dry and then stored in coin envelopes. We extracted genomic DNA from all samples using DNeasy Blood and Tissue Kits and a BioRobot 3000 following the manufacturer's specifications (Qiagen, Inc.).

### 2.3 Juvenile sampling

We sampled fin clips from 186 landlocked juveniles before stocking began (2013/2014) to represent the Rogers Lake landlocked population. We sampled fin clips from juveniles collected on August 15 and 16, 2017, from pelagic (offshore, 1,370 fish) and littoral (nearshore, 46 fish) habitats in Rogers Lake collected using a purse seine. DNA was extracted as described above. We sampled pelagic and littoral habitats because previous research has documented different patterns of habitat and resource use for anadromous and landlocked juveniles (Jones et al., 2013; Schielke, Palkovacs, & Post, 2011). Landlocked alewife are predominantly pelagic in their distribution and consume pelagic prey. In contrast, anadromous alewife also utilize littoral habitat, where they can be found at very high densities, and consume both pelagic and littoral prey before migrating to the ocean (Jones et al., 2013). Juveniles are able to out‐migrate from Rogers Lake using the fishway, which is open in the summer and fall to facilitate out‐migration, over the dam spillway, and through an outlet pipe that is used to control the water level in the lake.

### 2.4 Library preparation and microhaplotype identification

Many next‐generation DNA sequencing approaches provide phased short‐read sequences, which may contain multiple single nucleotide polymorphisms (SNPs) per fragment, or ‘microhaplotypes’ (Baetscher et al., 2018; Kidd et al., 2013, 2014). We developed microhaplotypes for alewife with data from double‐digest restriction site‐associated DNA sequencing (ddRAD‐seq; Peterson, Weber, Kay, Fisher, & Hoekstra, 2012) by identifying short regions of the genome (<150 bp) containing multiple SNPs and several haplotypes per region. Methodological details for microhaplotype development are described in Appendix S1.

### 2.5 Amplicon sequencing and bioinformatics pipeline

We generated amplicons targeting the selected microhaplotype loci using the Genotyping‐in‐Thousands by sequencing (GT‐seq) protocol described by Campbell, Harmon, and Narum (2015), with the modifications described in Baetscher et al. (2018). We ran prepared libraries, each containing 384 individuals on a MiSeq with a 2 × 75 bp paired‐end sequencing protocol. Following sequencing, individuals were de‐multiplexed, and raw paired‐end reads were merged using Fast Length Adjustment of SHort reads (FLASH; Magoč & Salzberg, 2011) allowing a minimum overlap of three nucleotides. We mapped reads to reference sequences (ddRAD‐seq data from which the loci were developed) with the Burrows–Wheeler Aligner using the MEM option (BWA‐MEM, Li & Durbin, 2009). We converted the mapped reads from SAM to BAM files with SAMtools (Li et al., 2009), and we used FreeBayes v. 1.1 (Garrison & Marth, 2012) to call SNPs in individual MiSeq runs. Individual VCF files were then merged using VCFtools (Danecek et al., 2011) to account for all SNPs detected in all sequencing runs.

We then used the package “*microhaplot*” (Ng, https://doi.org/10.5281/zenodo.820110) implemented in *R* v. 3.4.4 (R Core Development Team, 2018) to filter on read depth (10) and read depth ratio (0.2), to reduce sequencing errors (Baetscher et al., 2018). *microhaplot* uses the VCF file as a reference to identify target SNP sites in each locus. It extracts these sites from the SAM files and maintains the phase information from single reads to identify haplotypes and call final microhaplotypes from this haplotypic information. We removed individuals with at least 20% missing data to maintain genotype consistency across individuals. We also removed individuals with more than two haplotypes at several loci, indicating they were likely contaminated. The final datasets contained 183 (~98) landlocked juveniles from before stocking, 133 (~99%) stocked adults from 2015, 1,144 (100%) stocked adults from 2016, 2,749 stocked anadromous adults (~98%) from 2017, and 1,381 sampled juveniles (~98%) with genotypes that met our data criteria.

### 2.6 Assessing suitability of microhaplotypes for hybrid inference and parentage analyses

To evaluate whether the microhaplotype data were sufficiently powerful to confidently identify the ancestry of anadromous, landlocked, and hybrid alewife, we simulated 100 offspring from each of the pure and potential hybrid classes (F1, F2, and the reciprocal backcrosses) using HYBRIDLAB v. 1.0 (Nielsen, Bach, & Kotlicki, 2006) and the genotypes from our reference samples (anadromous adults stocked into Rogers Lake in 2017 and landlocked alewife juveniles before stocking). The simulated offspring genotypes were combined with those from the anadromous and landlocked reference samples and analyzed in NEWHYBRIDS v. 1.0 (Anderson & Thompson, 2002), which computes Bayesian posterior probabilities that each individual is in the parental, F1, F2, and reciprocal backcross categories. NEWHYBRIDS was run with a burn‐in of 10,000 replicates and 20,000 additional iterations. STRUCTURE v. 2.3.4 (Pritchard, Stephens, & Donnelly, 2000), a Bayesian model‐based clustering method, was also used to identify the ancestry of simulated individuals and infer the accuracy of ancestry assignment. STRUCTURE used the admixture model, correlated allele frequencies, and no prior on location/group. Ten iterations were run for *K* = 2, each including 50,000 burn‐in and 150,000 retained iterations of the simulations.

We used the package ‘*CKMRsim*’ (Anderson, https://doi.org/10.5281/zenodo.820162; Bravington, Skaug, & Anderson, 2016) to assess the power of the microhaplotype loci for pairwise pedigree inference in anadromous alewife populations. *CKMRsim* simulates related and unrelated individuals from estimates of allele frequencies, which were from 384 anadromous adults stocked into Rogers Lake. The probabilities of the related pairs (single parent–offspring, full‐sibling, and half‐sibling) and unrelated pairs, accounting for genotyping and sequencing errors, are used to compute the log‐likelihood ratio of the true relationship compared to the null hypothesis that they are unrelated. The distributions of these log‐likelihoods are compared to calculate the false‐positive and false‐negative rates. We then assessed the false‐positive rate at a false‐negative rate of 0.01.

### 2.7 Comparing microhaplotypes to SNPs for hybrid detection and parentage

To evaluate the power of the newly developed microhaplotypes for categorical hybrid assignment, we compared our results to those from individuals genotyped with a previously developed SNP panel (Baetscher, Hasselman, Reid, Palkovacs, & Garza, 2017). A subset of juveniles from 2017 (180), landlocked juveniles before stocking (50), and adults from Bride Brook (48) were genotyped with 96 SNP assays using 96.96 Dynamic SNP Genotyping Arrays on an EP1 system (Fluidigm Corporation) according to the manufacturer's specifications. Genotypes were called using the Fluidigm Genotyping Analysis Software v. 2.1.1. The SNPs were analyzed for their power to do categorical assignment following the same protocol described above. In addition, we also used *CKMRsim* to evaluate the most informative SNP per microhaplotype locus to provide a comparison between the inference from the microhaplotypes and SNPs for parent–offspring and full‐sibling pair identification.

### 2.8 Genetic diversity estimates and population characterization

The number of alleles and observed and expected heterozygosity for the stocked anadromous adults and landlocked alewife populations, and for the different classes of assigned juveniles, were calculated with the MICROSATELLITE TOOLKIT v. 3.1 (Park, 2001) using the microhaplotype data. Genetic differentiation between the anadromous and landlocked alewife populations was calculated in GENETIX v. 4.05 (Belkhir, Borsa, Chikhi, Raufaste, & Bonhomme, 1996–2004). STRUCTURE was run as described above to infer the ancestry of juveniles. The results were visualized using CLUMPP v. 1.1.2 (Jakobsson & Rosenberg, 2007) and DISTRUCT v. 1.1 (Rosenberg, 2004). NEWHYBRIDS was also used as described above to identify potential anadromous and hybrid offspring produced in 2017. As with the simulations, the same reference dataset as described above was used and included all the anadromous adults stocked into Rogers Lake in 2017 and the landlocked alewife before stocking. Individuals were assigned as landlocked, as anadromous, or as F1, F2, and reciprocal backcross hybrids. Clustering patterns of assigned juveniles were assessed with principal component analyses (PCA) implemented in the R package ‘*adegenet’* v. 2.0.1. (Jombart, 2008).

### 2.9 Pedigree reconstruction

To identify which anadromous adults produced offspring in the lake, we used maximum‐likelihood pedigree reconstruction with FRANz v. 2.0.0 (Riester, Standler, & Klemm, 2009). We ran both unconstrained and constrained analyses. Prior information provided for the constrained analysis was the year the adults were stocked, the year the offspring were born in, and analyses were run with and without sex, as assignments in the field can be inaccurate. All years of stocked adults (2015–2017) were analyzed as potential parents of the 2017 juveniles. We only retained parent–offspring assignments if there was a posterior probability greater than 0.98 and LOD score above 10. We also inferred sibling relationships through parent assignments and summarized the number of offspring per parent by sex, when applicable.

### 2.10 Estimates of abundance of landlocked, anadromous, and hybrids in Rogers Lake

Juvenile alewife densities were estimated from replicate sets of nighttime purse seines that encircles an area of 100 m^2^ conducted on August 15 and 16, 2017. We sampled after dark because alewife move up in the water column and spread out across the lake at night, allowing us to more effectively estimate juvenile population densities. We used three sets each in the littoral and the pelagic habitats of the lake. The habitat‐specific density estimates were then used to estimate habitat‐specific abundances of each alewife ancestry class (determined genetically) to estimate the total number of landlocked, anadromous, and hybrid juveniles in Rogers Lake for the different habitats and the lake overall.

## 3 RESULTS

### 3.1 Microhaplotype development and validation

**Figure 2:**
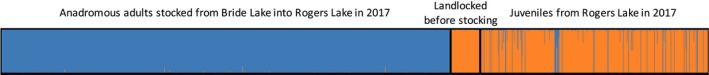
Model‐based clustering analysis of reference adult and unknown ancestry juvenile alewives (1,381 individuals) using STRUCTURE at *K* = 2. Anadromous adult alewife stocked from Bride Lake in 2017 are in blue (2,749 individuals), and landlocked alewife before stocking (2013/2014; 183 individuals) from Rogers Lake are in orange. Each line represents an individual, and the proportion of color indicates the posterior probability of ancestry to a specific ecotype

We retained 114 microhaplotype loci that met all filtering criteria (Table S1) and had 453 segregating alleles with 2–8 haplotypes per locus (Figure S1). Anadromous (Bride Lake) alewife had twice as many alleles per locus (~4.07) as the landlocked (Rogers Lake before stocking) alewife (2.02), and a similar pattern was observed for heterozygosity (Table 1). The anadromous and landlocked populations are quite genetically distinct (Figure 2) with *F*_ST_ = 0.12 (*p* < .001).

**Table 1:** Summary statistics for the Rogers Lake restoration project

Population	Year	Ecotype	Age	*N*	Loci	*N*a	*H*e	*H*o
Baseline								
Mill Brook	2015	Anadromous	Adults	133	114	3.91	0.46	0.43
Bride Brook	2016	Anadromous	Adults	1,144	114	4.17	0.47	0.45
Bride Brook	2017	Anadromous	Adults	2,749	114	4.07	0.47	0.46
Rogers lake	2013/2014	Landlocked	Juveniles	183	114	2.02	0.30	0.29
Juveniles								
Juveniles	2017	mixed	Juveniles	1,381	114	3.82	0.33	0.31
Anadromous	2017	Anadromous	Juveniles	88	114	3.59	0.47	0.45
F1	2017	Hybrid	Juveniles	27	114	3.24	0.47	0.52
Landlocked backcross	2017	Hybrid	Juveniles	33	114	2.94	0.40	0.40
Landlocked	2017	Landlocked	Juveniles	1,231	114	2.07	0.30	0.29

Note

The two anadromous backcross individuals were not included in the hybrid classes in this summary tables.

### 3.2 Microhaplotype accuracy for hybrid detection and pedigree reconstruction

Simulated anadromous and landlocked fish were assigned with 100% accuracy using NEWHYBRIDS (Figure S2, Table S2). Landlocked backcrosses were assigned correctly with 99% accuracy, with one individual being misidentified as an F2 hybrid. F1 and F2 hybrids, which can be difficult to distinguish even with a large amount of data (Anderson & Thompson, 2002; Veale & Russello, 2016), were relatively accurately assigned to their respective classes, with an error rate of ~4%. Assignments using STRUCTURE also provided 100% accuracy to pure ecotypic classes, with similar accuracy in assignments to hybrid classes, thus demonstrating the utility of the novel microhaplotype markers to distinguish between landlocked and anadromous ancestry, as well as to detect hybrids between these ecotypes (Figure S2).

The estimated false‐positive rate for parent–offspring pair assignments was 1.56 × 10^–9^ and for full siblings was 3.84 × 10^–7^. In 2017, there were at most 1.28 × 10^6^ parent–offspring pairs and 6.26 × 10^5^ full‐sibling comparisons necessary, indicating more than sufficient power to accurately infer parent–offspring and full‐sibling relationships within any year. In contrast, the false‐positive rate for identification of half‐sibling pairs was 9.20 × 10^–2^ (Figure S3), again emphasizing the large amount of data necessary to accurately identify them (Baetscher et al., 2018).

### 3.3 Microhaplotype versus SNP comparison

The novel microhaplotype markers we describe here provide more power than a similar number of SNP markers for both categorical (i.e., hybrid) identification and pedigree reconstruction. We evaluated the accuracy of these microhaplotype loci and a previously published set of 96 alewife SNPs (Baetscher et al., 2017) for identifying pure anadromous, pure landlocked, F1, and backcross alewife. To evaluate the relative accuracy of these markers, we compared mean q‐values (posterior probabilities of assignment) to the landlocked cluster for each pure and hybrid class. We found that the microhaplotypes (mean *q* = 1) and SNPs (mean *q* = 0.99, range 0.97–1) had similar power to discern the pure landlocked ecotype from all other classes. However, categorical assignment for the purely anadromous individuals was more accurate for the microhaplotypes (mean *q* = 0.00, range 0–0.01) than for the SNPs (mean *q* = 0.019, range 0–0.320). In addition, the range of *q*‐values for F1 and landlocked backcrosses did not overlap for the microhaplotypes, but did for the SNPs (data not shown), which is likely due to the higher number of loci and heterozygosity of the microhaplotypes.

The simulations using *CKMRsim* also demonstrated increased accuracy of these microhaplotype loci for single‐parent–offspring pair and full‐sibling identification (Figure S3). Whereas the false‐positive rates estimated above for the microhaplotypes indicate more than sufficient power to accurately identify these kin relationships with the required number of comparisons, the false‐positive rate estimated with SNPs at the same false‐negative rate was 1.12 × 10^–4^ for parent–offspring assignments and 1.27 × 10^–3^ for full‐sibling assignments, indicating that a similar number of SNPs would have resulted in multiple incorrect identifications.

### 3.4 Identification of landlocked, anadromous, and hybrid juveniles

**Figure 3:**
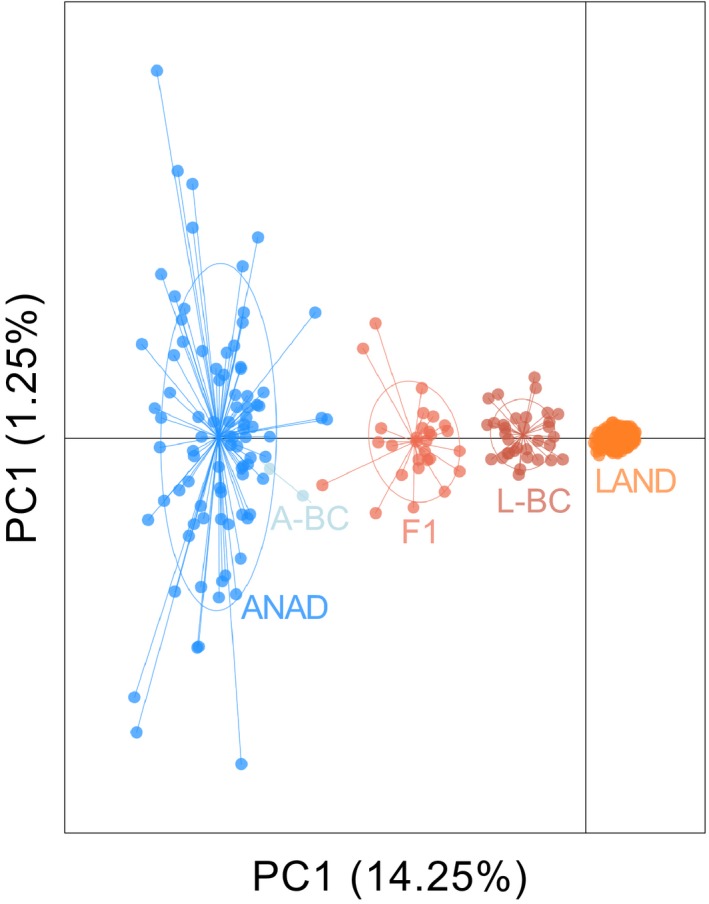
Principal component analysis of juveniles collected in 2017 colored by posterior probability assignment to class with NEWHYBRIDS. Class designations are pure anadromous (ANAD), pure landlocked (LAND), first‐generation hybrid (F1), landlocked backcross (L‐BC), and anadromous backcross (A‐BC)

Of the 1,381 juvenile alewife retained in the dataset after filtering, 1,231 (~89%) were identified as pure landlocked, 88 (6.4%) as pure anadromous, and 62 (4.6%) as hybrids (Figures 2 and 3b, Table S3). Hybrids were comprised of three distinct classes: F1 hybrids (27 individuals), landlocked backcrosses (33 individuals), and anadromous backcrosses (two individuals). No F2 juveniles were identified in 2017. Individual assignments to hybrid class are visualized in the PCA (Figure 3).

### 3.5 Parentage assignments and sibship groups

Parentage analysis confirmed the results from genetic hybrid identification. All confirmed anadromous juveniles collected in 2017 were assigned to adult anadromous alewife stocked in 2017 (no juveniles caught in 2017 were assigned to adults from 2015 or 2016). Both parents were identified for ~97% (85/88) of the putative anadromous juveniles and single parents identified for the other 3% (3/88). Similarly, a single anadromous parent was assigned to 96% (26/27) of putative F1 hybrids (Table S3) and to each of the two anadromous backcrosses. These assignments confirmed that all of these juveniles were born in 2017 because all of the parents were stocked in 2017. As expected, no pure landlocked juveniles or putative landlocked backcrosses were assigned to the potential anadromous parents. Of the 1,166 females stocked, offspring of 69 unique dams (6%) were identified, and of the 1,621 males stocked, 83 unique sires (5%) were identified. The majority (82%) of male and female parents produced a single sampled offspring; however, we identified sibling groups of size two, three, and four (Table S3).

Anadromous and F1 juveniles were produced from anadromous adults stocked on all three stocking dates in 2017 (Table 2, Table S3). These included adults stocked on the same and different stocking dates. Juvenile F1 hybrids were assigned to an equivalent number of male and female adults (13 each, Table S3). Six of the sibling groups we inferred from the parent–offspring identification had both anadromous and F1 hybrid offspring assigned to the same parent (four females, two males), meaning that these individuals spawned with both anadromous and landlocked partners.

**Table 2:** Summary of juveniles with assigned anadromous parents by stocking date

Stocking day	Anadromous			Hybrids
	March 30, 2017	April 12, 2017	April 19, 2017	
March 30, 2017	11 (12.9%)	13 (15.3%)	8 (9.4)	3 (11.5%)
April 12, 2017		28 (32.9%)	21 (24.7%)	13 (50.0%)
April 19, 2017			4 (4.7%)	10 (38.5%)

Note

Only anadromous fish that could have both parents assigned and hybrids with a parent assigned are reported in this table.

### 3.6 Abundance of landlocked, anadromous, and hybrid alewives

The landlocked alewives had the highest estimated juvenile abundance in the lake based on our density estimates and genetic identification (Table 3). Anadromous alewife juveniles were the second most abundant ecotype in the lake, and hybrids (F1 and landlocked backcrosses) were the least abundant in the lake. Different patterns of habitat abundance were observed for each of the ecotypes. The composition of anadromous, landlocked, and hybrid juveniles differed between the pelagic and littoral habitat (*p* < .01; *X* ^2^), but landlocked juveniles were the most abundant in both (Table 3). The highest estimated abundance of landlocked juveniles (74%) was found in the pelagic habitat with the highest estimated abundance of anadromous juveniles in the littoral habitat (76.5%). Hybrids were found at similar abundances in both habitats (Table 3).

**Table 3:** Estimates of total abundance and percent abundance in different habitats of juvenile alewife in Rogers Lake in August 2017

Ecotype	Total estimated abundance	Pelagic habitat (%)	Littoral habitat (%)
Landlocked	350,186 (CI: 144,867–555,506)	74	26
Anadromous	68,527 (CI: 34,803–102,252)	23.5	76.5
F1 hybrids	9,730 (CI: 5,344–14,116)	36	64
Landlocked backcrosses	13,223 (CI: 9,039–17,407)	53	47

## 4 DISCUSSION

We documented the magnitude and direction of introgression at the initiation of secondary contact between anadromous and landlocked alewife ecotypes in Rogers Lake, CT, USA, following anadromous alewife restoration efforts. We developed a novel panel of microhaplotype markers, which assign individuals to hybrid classes and ascertain parentage with high accuracy. We genotyped all anadromous adults stocked into Rogers Lake from the start of secondary contact (2015–2017) and a sample of 1,416 juveniles collected in 2017. We show that anadromous alewife spawned successfully in the lake, producing anadromous and hybrid offspring. Both sexes of anadromous adults stocked at three different dates in 2017 contributed to hybrids. The presence of landlocked backcrosses showed that at least some F1 hybrids, produced during previous stocking years, are maturing in the lake and spawning with landlocked partners, as no alewife were detected ascending the Rogers lake fishway during 2015–2017. The result of this pattern is directional gene flow from anadromous alewife into the landlocked population. We found that anadromous juveniles were more abundant in littoral habitat than pelagic habitat, with the opposite pattern observed for landlocked juveniles. This potential differential pattern of habitat use may indicate different resource use patterns, with potential implications for competition and successful anadromous alewife restoration.

In developing the microhaplotypes, we targeted regions of high variability in the genome to take advantage of the phased, short‐read information provided by next‐generation DNA sequencing platforms. This amplicon sequencing method allowed us to efficiently genotype thousands of individuals at loci that have high discriminatory power for hybrid and backcross classes, which can be difficult to distinguish, especially with limited genetic data (Elliot & Russello, 2018; Veale & Russello, 2016). We show through simulations that we can distinguish anadromous from landlocked ecotypes with 100% accuracy and landlocked backcrosses with 99% accuracy, which will be important as the restoration project continues. We were also able to correctly distinguish F1 from F2 hybrids with high (96%) accuracy using categorical assignments only. These microhaplotype markers are also extremely powerful for pedigree reconstruction applications including parentage analysis (Figure S3). Using parentage, we were able to verify the hybridization events in 2017 resulting in alewife juveniles. The simulation results show that both categorical assignments and parentage analyses are more accurate with microhaplotype markers than with an equivalent number of biallelic SNP markers, indicating that we are maximizing our inference power with the same number of sequences required to genotype single SNPs. The development and implementation of the microhaplotype panel also allowed us to reduce the genotyping cost per specimen (Meek & Larson, 2019).

We detected successful spawning of anadromous alewife in Rogers Lake. Nearly 6.5% of the juveniles collected in August 2017 were of purely anadromous origin. We could identify both parents of 97%, and single parents for the remaining 3%, of the anadromous juveniles (Table S3). The small number of unidentified parents is consistent with expectations, given the proportion (2%) of missing parental genotypes. Anadromous juveniles were produced by individuals from all stocking dates and not restricted to crosses between anadromous fish stocked on the same dates. This is consistent with previous observations that alewife have asynchronous oocyte development (Ganias et al., 2015) and can remain in freshwater from 1 to 12 weeks to spawn (Gahagan, Gherard, & Schultz, 2010; Kissil, 1974; Rosset et al., 2017). A recent study showed that introduced anadromous alewife adults remained in a lake and spawned with individuals from several stocking events before leaving the lake (Marjadi et al., 2018).

Landlocked and anadromous alewives are hybridizing in Rogers Lake at the onset of secondary contact, in spite of over 300 years of isolation (over 100 landlocked generations) and dramatically different life histories. Two years after the initiation of secondary contact, we identified hybrids representing two main hybrid classes—F1 hybrids (landlocked × anadromous) and landlocked backcross (landlocked × F1 adults). We identified 4.6% of juveniles as hybrids, with similar proportions of F1 hybrids and landlocked backcrosses. Just two sampled juveniles were putative anadromous backcrosses (anadromous × F1). Anadromous alewife juveniles migrate to the ocean from July to October, so it is possible that when sampling occurred, in early August, some juveniles would have already left the lake.

It appears that there are limited postzygotic barriers to hybridization between alewife ecotypes in Rogers Lake. Previous work has shown that postzygotic barriers between anadromous and resident ecotypes of salmonid fishes can form on short time frames and limit hybrid production through high mortality of developing eggs (Dion‐Côté et al., 2017), but that does not appear to be occurring here. Instead, prezygotic barriers such as spawning time may limit F1 hybrid formation in alewives. Reproductive timing, in particular, may play a role in limiting hybrid formation (Turbek, Scordato, & Safran, 2018). Anadromous alewives in this region generally spawn earlier in the year and for a shorter duration than landlocked populations (Littrell et al., 2018). The frequency of hybrids we observed (4.6%) is roughly consistent with the percent spawning time overlap (3%) observed among isolated anadromous and landlocked populations in the study period, supporting our hypothesis that spawning time overlap serves to limit the extent of hybridization.

Patterns of hybridization did not show evidence of size selection for mates. Anadromous adults are substantially larger than landlocked adults (Palkovacs et al., 2008), so landlocked and anadromous males might favor the larger anadromous females, as seen in salmonids with similar life history variation (Hutchings & Myers, 1985, 1988). If so, we would expect the majority of hybrids to have landlocked fathers and anadromous mothers. However, we found that male and female anadromous fish contributed equally to hybrids. In addition, we identified families consisting of both anadromous and hybrid offspring, so some anadromous individuals spawned with both anadromous and landlocked partners. It is thought that alewife aggregate to spawn, with one female to several males, and then release eggs and sperm into the water column where fertilization takes place (Marjadi et al., 2018). It appears that this broadcast spawning limits opportunities for mate selection relative to fish species that pair and build nests, such as salmonids and stickleback (Hutchings & Meyer, 1985; Kraak & Bakker, 1998).

The direction of introgression we observed at this early stage of secondary contact was predominantly from the anadromous to the landlocked population, and appears to be driven by the maturation of F1 hybrids in the lake. These hybrid individuals would have had to be born in 2015 and 2016 and matured in the lake, as no alewife ascended the Rogers Lake fishway from 2015 to 2017. Landlocked alewife are thought to sexually mature at a younger age (1–2 years of age; Nigro & Ney, 1982) than anadromous alewife (3–4 years of age; Davis & Schultz, 2009). The presence of anadromous and landlocked backcrosses indicates that F1 hybrids are maturing at similar ages to the landlocked adults in the lake. Furthermore, the majority of backcrosses were to landlocked fish, showing that F1 hybrids that mature in the lake spawned with more landlocked individuals, likely due to their higher abundance and similar spawning cues experienced in the lake. Only two putative anadromous backcross individuals were found, and they were progeny of two different female anadromous parents from different stocking dates in 2017 with adult F1 hybrids born in previous years.

Several recent studies have shown that there is a loss of important migratory traits in landlocked alewife populations, which include reduced salinity tolerance, and osmoregulatory and swimming ability (Velotta, McCormick, Jones, & Schultz, 2018; Velotta, McCormick, & Schultz, 2015; Velotta et al., 2017), which may lead to lower fitness of hybrids attempting to migrate and survive in the ocean. Evidence that hybrids might have poor survival in the marine environment, coupled with our results showing the directional movement of alleles from the anadromous population into the landlocked population, suggests that maladaptation due to the introgression of landlocked alleles into the anadromous population may not be a major factor limiting anadromous alewife restoration.

The spatial distribution of each ecotype varied by habitat, with higher abundance of anadromous juveniles estimated in the littoral habitats than pelagic habitats in the lake. Landlocked juveniles were found in the highest abundances in the pelagic habitats than littoral (Table 3). In contrast, hybrid abundance did not vary significantly by habitat type. Prior work has shown that when pelagic zooplankton resources become depleted, anadromous juveniles switch to feeding in the littoral zone, whereas landlocked alewives stay in the pelagic zone and feed on zooplankton even when the abundance of this resource is low (Jones et al., 2013; Schielke et al., 2011). This differential resource use may reduce competition between ecotypes and allow restoration of anadromous alewife in lakes with large extant populations of landlocked alewife. Important changes in the ecology and evolution of lake communities have happened as a result of differences between anadromous and landlocked alewife feeding traits (Boel et al., 2018; Broderson et al., 2015; Huss et al., 2014; Palkovacs & Post, 2008, 2009; Post et al., 2008; Walsh et al., 2012; Walsh & Post, 2012). The resource use patterns that evolve with secondary contact in Rogers Lake will likely have consequences for ecological dynamics as this restoration project proceeds.

## 5 CONCLUSIONS

Our results provide several important insights into the ecological and evolutionary dynamics during the initial stages of secondary contact. We show that a relatively small number of individuals, re‐introduced into historical habitat as part of a restoration effort, can reproduce successfully. We also show that more than a hundred generations of physical isolation and associated phenotypic evolution, including shifts in morphology and the timing of reproduction, can reduce—but not eliminate—the potential for hybridization. We found directional introgression of alleles from the re‐introduced anadromous fish into the larger resident landlocked population within 3 years of initial secondary contact. We found very limited movement of landlocked alleles into the anadromous population, which may be a sign that the maladaptive flow of alleles will have little effect on anadromous alewife restoration. Finally, we show that anadromous and landlocked alewife are found at different abundances in the littoral and pelagic habitat, indicating potential differences in resource use that may mediate competition between the ecotypes as the restoration of anadromous fish in Rogers Lake continues. Thus, understanding the traits of F1 hybrids is critical to understanding how the dynamics of secondary contact will play out. Importantly, such information on F1 hybrids can only be obtained if secondary contact is tracked from its onset.

## CONFLICT OF INTEREST

None declared.

## Data Availability

The merged VCF file, Reference sequence file, and called haplotypes for alewives are deposited on the Dryad Digital Repository https://doi.org/10.5061/dryad.70rxwdbt0.

## References

[eva12890-bib-0001] Anderson, E. C. , & Thompson, E. A. (2002). A model‐based method for identifying species hybrids using multilocus genetic data. Genetics, 160, 1217–1229. 11901135 10.1093/genetics/160.3.1217 PMC1462008

[eva12890-bib-0002] Araki, H. , Cooper, B. , & Blouin, M. S. (2007). Genetic effects of captive breeding cause a rapid, cumulative fitness decline in the wild. Science, 318, 100–103. 10.1126/science.1145621 17916734

[eva12890-bib-0003] Arnegard, M. E. , McGee, M. D. , Matthews, B. , Marchinko, K. B. , Conte, G. L. , Kabir, S. , … Schluter, D. (2014). Genetics of ecological divergence during speciation. Nature, 511, 307–311. 10.1038/nature13301 24909991 PMC4149549

[eva12890-bib-0004] Atlantic States Marine Fisheries Commission (2012). River herring benchmark stock assessment. Stock Assessment Report No. 12‐02 of the Atlantic States Marine Fisheries Commission Vol. 1 Washington, DC.

[eva12890-bib-0005] Baetscher, D. S. , Clemento, A. J. , Ng, T. C. , Anderson, E. C. , & Garza, J. C. (2018). Microhaplotypes provide increased power from short‐read DNA sequences for relationship inference. Molecular Ecology Resources, 18, 296–305. 10.1111/1755-0998.12737 29143457

[eva12890-bib-0006] Baetscher, D. S. , Hasselman, D. J. , Reid, K. , Palkovacs, E. P. , & Garza, J. C. (2017). Discovery and characterization of single nucleotide polymorphisms in two anadromous alosine fishes of conservation concern. Ecology and Evolution, 7, 6638–6648. 10.1002/ece3.3215 28904746 PMC5587496

[eva12890-bib-0007] Belkhir, K. , Borsa, P. , Chikhi, L. , Raufaste, N. , & Bonhomme, F. (1996–2004). GENETIX 4.05, logiciel sous Windows TM pour la génétique des populations. Montpellier, France: Laboratoire Génome, Populations, Interactions, CNRS UMR 5171, Université de Montpellier II.

[eva12890-bib-0008] Boel, M. , Brodersen, J. , Baktoft, H. , Koed, A. , & Post, D. M. (2018). Incidence and phenotypic variation in alewife alter the ontogenetic trajectory of young‐of‐the‐year largemouth bass. Oikos, 127, 1800–1811. 10.1111/oik.05556

[eva12890-bib-0009] Bravington, M. V. , Skaug, H. J. , & Anderson, E. C. (2016). Close‐kin mark‐recapture. Statistical Science, 31, 259–274. 10.1214/16-STS552

[eva12890-bib-0010] Brodersen, J. , Howeth, J. G. , & Post, D. M. (2015). Emergence of a novel prey life history promotes contemporary sympatric diversification in a top predator. Nature Communications, 6, 1–9. 10.1038/ncomms9115 26365323

[eva12890-bib-0011] Butlin, R. K. , & Smadja, C. M. (2018). Coupling, reinforcement, and speciation. The American Naturalist, 191, 155–172. 10.1086/695136 29351021

[eva12890-bib-0012] Campbell, N. R. , Harmon, S. A. , & Narum, S. R. (2015). Genotyping‐in‐Thousands by sequencing (GT‐seq): A cost effective SNP genotyping method based on custom amplicon sequencing. Molecular Ecology Resources, 15, 855–867. 10.1111/1755-0998.12357 25476721

[eva12890-bib-0013] Clemento, A. J. , Anderson, E. C. , Boughton, D. , Girman, D. , & Garza, J. C. (2009). Population genetic structure and ancestry of *Oncorhynchus mykiss* populations above and below dams in south‐central California. Conservation Genetics, 10, 1321–1337. 10.1007/s10592-008-9712-0

[eva12890-bib-0014] Closs, G. P. , Hicks, A. S. , & Jellyman, P. G. (2013). Life histories of closely related amphidromous and non‐migratory fish species: A trade‐off between egg size and fecundity. Freshwater Biology, 58, 1162–1177. 10.1111/fwb.12116

[eva12890-bib-0016] Crispo, E. , Moore, J. S. , Lee‐Yaw, J. A. , Gray, S. M. , & Haller, B. C. (2011). Broken barriers: Human‐induced changes to gene flow and introgression in animals: An examination of the ways in which humans increase genetic exchange among populations and species and the consequences for biodiversity. BioEssays, 33, 508–518. 10.1002/bies.201000154 21523794

[eva12890-bib-0017] Danecek, P. , Auton, A. , Abecasis, G. , Albers, C. A. , Banks, E. , DePristo, M. A. , … Durbin, R. (2011). The variant call format and VCFtools. Bioinformatics, 27, 2156–2158. 10.1093/bioinformatics/btr330 21653522 PMC3137218

[eva12890-bib-0018] Davis, J. P. , & Schultz, E. T. (2009). Temporal shifts in demography and life history of an anadromous alewife population in Connecticut. Marine and Coastal Fisheries, 1, 90–106. 10.1577/C08-003.1

[eva12890-bib-0019] Dion‐Côté, A. M. , Symonová, R. , Lamaze, F. C. , Pelikánová, Š. , Ráb, P. , & Bernatchez, L. (2017). Standing chromosomal variation in Lake Whitefish species pairs: The role of historical contingency and relevance for speciation. Molecular Ecology, 26, 178–192. 27545583 10.1111/mec.13816

[eva12890-bib-0020] Elliott, L. , & Russello, M. A. (2018). SNP panels for differentiating advanced‐generation hybrid classes in recently diverged stocks: A sensitivity analysis to inform monitoring of sockeye salmon re‐stocking programs. Fisheries Research, 208, 339–345. 10.1016/j.fishres.2018.09.001

[eva12890-bib-0021] Franssen, N. R. , Harris, J. , Clark, S. R. , Schaefer, J. F. , & Stewart, L. K. (2013). Shared and unique morphological responses of stream fishes to anthropogenic habitat alteration. Proceedings of the Royal Society of London B: Biological Sciences, 280, 20122715. 10.1098/rspb.2012.2715 PMC3574318 23235710

[eva12890-bib-0022] Gahagan, B. I. , Gherard, K. E. , & Schultz, E. T. (2010). Environmental and endogenous factors influencing emigration in juvenile anadromous alewives. Transactions of the American Fisheries Society, 139, 1069–1082. 10.1577/T09-128.1

[eva12890-bib-0023] Ganias, K. , Divino, J. N. , Gherard, K. E. , Davis, J. P. , Mouchlianitis, F. , & Schultz, E. T. (2015). A reappraisal of reproduction in anadromous alewives: Determinate versus indeterminate fecundity, batch size, and batch number. Transactions of the American Fisheries Society, 144, 1143–1158. 10.1080/00028487.2015.1073620

[eva12890-bib-0024] Garrick, R. C. , Benavides, E. , Russello, M. A. , Gibbs, J. P. , Poulakakis, N. , Dion, K. B. , … Caccone, A. (2012). Genetic rediscovery of an ‘extinct’ Galápagos giant tortoise species. Current Biology, 22, R10–R11. 10.1016/j.cub.2011.12.004 22240469

[eva12890-bib-0025] Garrick, R. C. , Benavides, E. , Russello, M. A. , Hyseni, C. , Edwards, D. L. , Gibbs, J. P. , … Caccone, A. (2014). Lineage fusion in Galápagos giant tortoises. Molecular Ecology, 23, 5276–5290. 10.1111/mec.12919 25223395

[eva12890-bib-0026] Garrison, E. , & Marth, G. (2012). Haplotype‐based variant detection from short‐read sequencing. arXiv:1207.3907v2, 9.

[eva12890-bib-0027] Gurnell, J. , Wauters, L. A. , Lurz, P. W. , & Tosi, G. (2004). Alien species and interspecific competition: Effects of introduced eastern grey squirrels on red squirrel population dynamics. Journal of Animal Ecology, 73, 26–35. 10.1111/j.1365-2656.2004.00791.x

[eva12890-bib-0028] Hamilton, J. A. , & Miller, J. M. (2016). Adaptive introgression as a resource for management and genetic conservation in a changing climate. Conservation Biology, 30, 3–41. 10.1111/cobi.12574 26096581

[eva12890-bib-0029] Hasselman, D. J. , Anderson, E. C. , Argo, E. E. , Bethoney, N. D. , Gephard, S. R. , Post, D. M. , … Palkovacs, E. P. (2015). Genetic stock composition of marine bycatch reveals disproportional impacts on depleted river herring genetic stocks. Canadian Journal of Fisheries and Aquatic Sciences, 73, 951–963.

[eva12890-bib-0030] Hasselman, D. J. , Argo, E. E. , McBride, M. C. , Bentzen, P. , Schultz, T. F. , Perez‐Umphrey, A. A. , & Palkovacs, E. P. (2014). Human disturbance causes the formation of a hybrid swarm between two naturally sympatric fish species. Molecular Ecology, 23, 1137–1152. 10.1111/mec.12674 24450302

[eva12890-bib-0031] Hasselman, D. J. , & Limburg, K. E. (2012). Alosine restoration in the 21st century: Challenging the status quo. Marine and Coastal Fisheries, 4, 174–187. 10.1080/19425120.2012.675968

[eva12890-bib-0032] Hedrick, P. W. (2013). Adaptive introgression in animals: Examples and comparison to new mutation and standing variation as sources of adaptive variation. Molecular Ecology, 22, 4606–4618. 10.1111/mec.12415 23906376

[eva12890-bib-0033] Huss, M. , Howeth, J. G. , Osterman, J. I. , & Post, D. M. (2014). Intraspecific phenotypic variation among alewife populations drives parallel shifts in bluegill. Proceedings of the Royal Society of London B: Biological Sciences, 281, 20140275. 10.1098/rspb.2014.0275 PMC4071535 24920478

[eva12890-bib-0034] Hutchings, J. A. , & Myers, R. A. (1985). Mating between anadromous and nonanadromous Atlantic salmon, *Salmo salar* . Canadian Journal of Zoology, 63, 2219–2221.

[eva12890-bib-0035] Hutchings, J. A. , & Myers, R. A. (1988). Mating success of alternative maturation phenotypes in male Atlantic salmon, *Salmo salar* . Oecologia, 75, 169–174. 10.1007/BF00378593 28310830

[eva12890-bib-0036] Jakobsson, M. , & Rosenberg, N. A. (2007). CLUMPP: A cluster matching and permutation program for dealing with label switching and multimodality in analysis of population structure. Bioinformatics, 23, 1801–1806. 10.1093/bioinformatics/btm233 17485429

[eva12890-bib-0037] Jombart, T. (2008). adegenet: A R package for the multivariate analysis of genetic markers. Bioinformatics, 24, 1403–1405. 10.1093/bioinformatics/btn129 18397895

[eva12890-bib-0038] Jones, A. W. , Palkovacs, E. P. , & Post, D. M. (2013). Recent parallel divergence in body shape and diet source of alewife life history forms. Evolutionary Ecology, 27, 1175–1187. 10.1007/s10682-013-9650-2

[eva12890-bib-0040] Jones, F. C. , Brown, C. , Pemberton, J. M. , & Braithwaite, V. A. (2006). Reproductive isolation in a threespine stickleback hybrid zone. Journal of Evolutionary Biology, 19, 1531–1544. 10.1111/j.1420-9101.2006.01122.x 16910983

[eva12890-bib-0041] Kidd, K. K. , Pakstis, A. J. , Speed, W. C. , Lagacé, R. , Chang, J. , Wootton, S. , … Kidd, J. R. (2014). Current sequencing technology makes microhaplotypes a powerful new type of genetic marker for forensics. Forensic Science International: Genetics, 12, 215–224. 10.1016/j.fsigen.2014.06.014 25038325

[eva12890-bib-0042] Kidd, K. K. , Pakstis, A. J. , Speed, W. C. , Lagace, R. , Chang, J. , Wootton, S. , & Ihuegbu, N. (2013). Microhaplotype loci are a powerful new type of forensic marker. Forensic Science International: Genetics Supplement Series, 4, e123–e124.

[eva12890-bib-0043] Kissil, G. W. (1974). Spawning of the anadromous alewife, *Alosa pseudoharengus*, in Bride Lake, Connecticut. Transactions of the American Fisheries Society, 103, 312–317. 10.1577/1548-8659(1974)103<312:SOTAAA>2.0.CO;2

[eva12890-bib-0044] Kraak, S. B. , & Bakker, T. C. (1998). Mutual mate choice in sticklebacks: Attractive males choose big females, which lay big eggs. Animal Behaviour, 56, 859–866. 10.1006/anbe.1998.0822 9790696

[eva12890-bib-0045] Lamichhaney, S. , Han, F. , Webster, M. T. , Andersson, L. , Grant, B. R. , & Grant, P. R. (2018). Rapid hybrid speciation in Darwin’s finches. Science, 359, 224–228. 10.1126/science.aao4593 29170277

[eva12890-bib-0046] Levine, J. M. , & HilleRisLambers, J. (2009). The importance of niches for the maintenance of species diversity. Nature, 461, 254–257. 10.1038/nature08251 19675568

[eva12890-bib-0047] Li, H. , & Durbin, R. (2009). Fast and accurate short read alignment with Burrows‐Wheeler transform. Bioinformatics, 25, 1754–1760. 10.1093/bioinformatics/btp324 19451168 PMC2705234

[eva12890-bib-0048] Li, H. , Handsaker, B. , Wysoker, A. , Fennell, T. , Ruan, J. , Homer, N. , … Durbin, R. (2009). The sequence alignment/map format and SAMtools. Bioinformatics, 25, 2078–2079. 10.1093/bioinformatics/btp352 19505943 PMC2723002

[eva12890-bib-0049] Littrell, K. A. , Ellis, D. , Gephard, S. R. , MacDonald, A. D. , Palkovacs, E. P. , Scranton, K. , & Post, D. M. (2018). Evaluating the potential for pre‐zygotic isolation and hybridization between landlocked and anadromous alewife (*Alosa pseudoharengus*) following secondary contact. Evolutionary Applications, 11, 1554–1566. 30344627 10.1111/eva.12645 PMC6183454

[eva12890-bib-0050] Loesch, J. G. (1987). Overview of life history aspects of anadromous alewife and blueback herring in freshwater habitats. American Fisheries Society Symposium, 1, 89–103.

[eva12890-bib-0052] Magoč, T. , & Salzberg, S. L. (2011). FLASH: Fast length adjustment of short reads to improve genome assemblies. Bioinformatics, 27, 2957–2963. 10.1093/bioinformatics/btr507 21903629 PMC3198573

[eva12890-bib-0053] Marjadi, M. N. , Roy, A. H. , Jordaan, A. , Gahagan, B. I. , Armstrong, M. P. , & Whiteley, A. R. (2018). Larger body size and earlier run timing increase alewife reproductive success in a whole lake experiment. Canadian Journal of Fisheries and Aquatic Sciences, 76(7), 1134–1146. 10.1139/cjfas-2017-0451

[eva12890-bib-0054] Mayfield, M. M. , & Levine, J. M. (2010). Opposing effects of competitive exclusion on the phylogenetic structure of communities. Ecology Letters, 13, 1085–1093. 10.1111/j.1461-0248.2010.01509.x 20576030

[eva12890-bib-0055] McKinney, G. J. , Seeb, J. E. , & Seeb, L. W. (2017). Managing mixed‐stock fisheries: Genotyping multi‐SNP haplotypes increases power for genetic stock identification. Canadian Journal of Fisheries and Aquatic Sciences, 74, 429–434. 10.1139/cjfas-2016-0443

[eva12890-bib-0056] Mech, S. G. , & Hallett, J. G. (2001). Evaluating the effectiveness of corridors: A genetic approach. Conservation Biology, 15, 467–474. 10.1046/j.1523-1739.2001.015002467.x

[eva12890-bib-0057] Meek, M. H. , & Larson, W. A. (2019). The future is now: Amplicon sequencing and sequence capture usher in the conservation genomics era. Molecular Ecology Resources, 19, 795–803. 10.1111/1755-0998.12998 30681776

[eva12890-bib-0059] Morissette, O. , Sirois, P. , Lester, N. P. , Wilson, C. C. , & Bernatchez, L. (2018). Supplementation stocking of Lake Trout (*Salvelinus namaycush*) in small boreal lakes: Ecotypes influence on growth and condition. PLoS ONE, 13, e0200599 10.1371/journal.pone.0200599 30001412 PMC6042763

[eva12890-bib-0060] Nielsen, E. E. , Bach, L. A. , & Kotlicki, P. (2006). HYBRIDLAB (version 1.0): A program for generating simulated hybrids from population samples. Molecular Ecology Notes, 6, 971–973. 10.1111/j.1471-8286.2006.01433.x

[eva12890-bib-0061] Nigro, A. A. , & Ney, J. J. (1982). Reproduction and early‐life accommodations of landlocked alewives to a southern range extension. Transactions of the American Fisheries Society, 111, 559–569. 10.1577/1548-8659(1982)111<559:RAEAOL>2.0.CO;2

[eva12890-bib-0063] Palkovacs, E. P. , Dion, K. B. , Post, D. M. , & Caccone, A. (2008). Independent evolutionary origins of landlocked alewife populations and rapid parallel evolution of phenotypic traits. Molecular Ecology, 17, 582–597. 10.1111/j.1365-294X.2007.03593.x 18179439

[eva12890-bib-0064] Palkovacs, E. P. , Hasselman, D. J. , Argo, E. E. , Gephard, S. R. , Limburg, K. E. , Post, D. M. , … Willis, T. V. (2014). Combining genetic and demographic information to prioritize conservation efforts for anadromous alewife and blueback herring. Evolutionary Applications, 7, 212–226. 10.1111/eva.12111 24567743 PMC3927884

[eva12890-bib-0065] Palkovacs, E. P. , Mandeville, E. G. , & Post, D. M. (2014). Contemporary trait change in a classic ecological experiment: Rapid decrease in alewife gill‐raker spacing following introduction to an inland lake. Freshwater Biology, 59, 1897–1901. 10.1111/fwb.12392

[eva12890-bib-0066] Palkovacs, E. P. , & Post, D. M. (2008). Eco‐evolutionary interactions between predators and prey: Can predator‐induced changes to prey communities feed back to shape predator foraging traits? Evolutionary Ecology Research, 10, 699–720.

[eva12890-bib-0067] Palkovacs, E. P. , & Post, D. M. (2009). Experimental evidence that phenotypic divergence in predators drives community divergence in prey. Ecology, 90, 300–305. 10.1890/08-1673.1 19323211

[eva12890-bib-0068] Park, S. D. E. (2001). The Excel microsatellite toolkit (version 3.1). Dublin, Ireland: Animal Genomics Laboratory, University College Dublin.

[eva12890-bib-0069] Pearse, D. E. , Miller, M. R. , Abadía‐Cardoso, A. , & Garza, J. C. (2014). Rapid parallel evolution of standing variation in a single, complex, genomic region is associated with life history in steelhead/rainbow trout. Proceedings of the Royal Society of London B: Biological Sciences, 281, 20140012. 10.1098/rspb.2014.0012 PMC3996610 24671976

[eva12890-bib-0070] Perry, W. L. , Feder, J. L. , Dwyer, G. , & Lodge, D. M. (2001). Hybrid zone dynamics and species replacement between *Orconectes* crayfishes in a northern Wisconsin Lake. Evolution, 55, 1153–1166. 10.1111/j.0014-3820.2001.tb00635.x 11475051

[eva12890-bib-0071] Peterson, B. K. , Weber, J. N. , Kay, E. H. , Fisher, H. S. , & Hoekstra, H. E. (2012). Double digest RADseq: an inexpensive method for de novo SNP discovery and genotyping in model and non-model species. PloS one, 7(5), e37135. 22675423 10.1371/journal.pone.0037135 PMC3365034

[eva12890-bib-0072] Post, D. M. , Palkovacs, E. P. , Schielke, E. G. , & Dodson, S. I. (2008). Intraspecific variation in a predator affects community structure and cascading trophic interactions. Ecology, 89, 2019–2032. 10.1890/07-1216.1 18705387

[eva12890-bib-0073] Pritchard, J. K. , Stephens, M. , & Donnelly, P. (2000). Inference of population structure using multilocus genotype data. Genetics, 155, 945–959. 10835412 10.1093/genetics/155.2.945 PMC1461096

[eva12890-bib-0074] R Core Development Team (2018). R: A language and environment for statistical computing. Vienna, Austria: R Core Development Team.

[eva12890-bib-0075] Reid, K. , Palkovacs, E. P. , Hasselman, D. J. , Baetscher, D. , Kibele, J. , Gahagan, B. , … Garza, J. C. (2018). Comprehensive evaluation of genetic population structure for anadromous river herring with single nucleotide polymorphism data. Fisheries Research, 206, 247–258. 10.1016/j.fishres.2018.04.014

[eva12890-bib-0076] Rhymer, J. M. , & Simberloff, D. (1996). Extinction by hybridization and introgression. Annual Review of Ecology and Systematics, 27, 83–109. 10.1146/annurev.ecolsys.27.1.83

[eva12890-bib-0077] Riester, M. , Stadler, P. F. , & Klemm, K. (2009). FRANz: Reconstruction of wild multi‐generation pedigrees. Bioinformatics, 25, 2134–2139. 10.1093/bioinformatics/btp064 19202194 PMC2722992

[eva12890-bib-0078] Rius, M. , & Darling, J. A. (2014). How important is intraspecific genetic admixture to the success of colonising populations? Trends in Ecology & Evolution, 29, 233–242. 10.1016/j.tree.2014.02.003 24636862

[eva12890-bib-0079] Rosenberg, N. A. (2004). DISTRUCT: A program for the graphical display of population structure. Molecular Ecology Notes, 4, 137–138. 10.1046/j.1471-8286.2003.00566.x

[eva12890-bib-0080] Rosset, J. , Roy, A. H. , Gahagan, B. I. , Whiteley, A. R. , Armstrong, M. P. , Sheppard, J. J. , & Jordaan, A. (2017). Temporal patterns of migration and spawning of river herring in coastal Massachusetts. Transactions of the American Fisheries Society, 146, 1101–1114. 10.1080/00028487.2017.1341851

[eva12890-bib-0082] Saunders, D. A. , Hobbs, R. J. , & Margules, C. R. (1991). Biological consequences of ecosystem fragmentation: A review. Conservation Biology, 5, 18–32. 10.1111/j.1523-1739.1991.tb00384.x

[eva12890-bib-0083] Schielke, E. G. , Palkovacs, E. P. , & Post, D. M. (2011). Eco‐evolutionary feedbacks drive niche differentiation in the alewife. Biological Theory, 6, 211–219. 10.1007/s13752-012-0031-9

[eva12890-bib-0084] Seehausen, O. , Takimoto, G. , Roy, D. , & Jokela, J. (2008). Speciation reversal and biodiversity dynamics with hybridization in changing environments. Molecular Ecology, 17, 30–44. 10.1111/j.1365-294X.2007.03529.x 18034800

[eva12890-bib-0085] Tewksbury, J. J. , Levey, D. J. , Haddad, N. M. , Sargent, S. , Orrock, J. L. , Weldon, A. , … Townsend, P. (2002). Corridors affect plants, animals, and their interactions in fragmented landscapes. Proceedings of the National Academy of Sciences of the United States of America, 99, 12923–12926. 10.1073/pnas.202242699 12239344 PMC130561

[eva12890-bib-0086] Todesco, M. , Pascual, M. A. , Owens, G. L. , Ostevik, K. L. , Moyers, B. T. , Hübner, S. , … Rieseberg, L. H. (2016). Hybridization and extinction. Evolutionary Applications, 9, 892–908. 10.1111/eva.12367 27468307 PMC4947151

[eva12890-bib-0087] Tulp, I. , Keller, M. , Navez, J. , Winter, H. V. , de Graaf, M. , & Baeyens, W. (2013). Connectivity between migrating and landlocked populations of a diadromous fish species investigated using otolith microchemistry. PLoS ONE, 8, e69796 10.1371/journal.pone.0069796 23922803 PMC3726755

[eva12890-bib-0088] Turbek, S. P. , Scordato, E. S. , & Safran, R. J. (2018). The role of seasonal migration in population divergence and reproductive isolation. Trends in Ecology & Evolution, 33, 164–175. 10.1016/j.tree.2017.11.008 29289354

[eva12890-bib-0089] Twining, C. W. , & Post, D. M. (2013). Cladoceran remains reveal presence of a keystone size‐selective planktivore. Journal of Paleolimnology, 49, 253–266. 10.1007/s10933-012-9672-8

[eva12890-bib-0090] Twining, C. W. , West, D. C. , & Post, D. M. (2013). Historical changes in nutrient inputs from humans and anadromous fishes in New England's coastal watersheds. Limnology and Oceanography, 58, 1286–1300. 10.4319/lo.2013.58.4.1286

[eva12890-bib-0091] Veale, A. J. , & Russello, M. A. (2016). Sockeye salmon repatriation leads to population re‐establishment and rapid introgression with native kokanee. Evolutionary Applications, 9, 1301–1311. 10.1111/eva.12430 27877207 PMC5108220

[eva12890-bib-0092] Velotta, J. P. , McCormick, S. D. , Jones, A. W. , & Schultz, E. T. (2018). Reduced swimming performance repeatedly evolves on loss of migration in landlocked populations of alewife. Physiological and Biochemical Zoology, 91, 814–825. 10.1086/696877 29381120

[eva12890-bib-0093] Velotta, J. P. , McCormick, S. D. , & Schultz, E. T. (2015). Trade‐offs in osmoregulation and parallel shifts in molecular function follow ecological transitions to freshwater in the Alewife. Evolution, 69, 2676–2688. 10.1111/evo.12774 26374626

[eva12890-bib-0094] Velotta, J. P. , Wegrzyn, J. L. , Ginzburg, S. , Kang, L. , Czesny, S. , O'Neill, R. J. , … Schultz, E. T. (2017). Transcriptomic imprints of adaptation to fresh water: Parallel evolution of osmoregulatory gene expression in the Alewife. Molecular Ecology, 26, 831–848. 10.1111/mec.13983 28012221

[eva12890-bib-0095] Walsh, M. R. , DeLong, J. P. , Hanley, T. C. , & Post, D. M. (2012). A cascade of evolutionary change alters consumer‐resource dynamics and ecosystem function. Proceedings of the Royal Society B: Biological Sciences, 279, 3184–3192. 10.1098/rspb.2012.0496 PMC3385726 22628469

[eva12890-bib-0096] Walsh, M. R. , & Post, D. M. (2012). The impact of intraspecific variation in a fish predator on the evolution of phenotypic plasticity and investment in sex in *Daphnia ambigua* . Journal of Evolutionary Biology, 25, 80–89. 10.1111/j.1420-9101.2011.02403.x 22022990

