# Supplementary material for: Restoration‐mediated secondary contact leads to introgression of alewife ecotypes separated by a colonial‐era dam
Source: Evol Appl. 2019 Nov 18;13(4):652–64. doi: 10.1111/eva.12890 (PMC7086056; doi:10.1111/eva.12890)
Supplement: Supplementary file 1 [file EVA-13-652-s001.pdf]

Supplementary Table S1 Microhaplotype primer panel for Gtseq and sequencing on an Illumina Miseq

	Locus	Primers	5'-3' (Primers with RNA primer sequence for Miseq added)	Dil/Enr	Notes
1	tag_id_10174	Aps_10174_AN_F	CGACAGGTTCAGAGTTCTACAGTCCGACGATCTTCACAATGCAGTACAACACAAG		
		Aps_10174_AN_R	GTGACTGGAGTTCAGACGTGTGCTCTTCCGATCTCACCGCTCTGAACTAAATGCT		
2	tag_id_3_1027	Aps_1027_R3_F	CGACAGGTTCAGAGTTCTACAGTCCGACGATCCTCGTTTCTTGCGGTTTC		
		Aps_1027_R3_R	GTGACTGGAGTTCAGACGTGTGCTCTTCCGATCTGTTACGAGGCACATTTGG		
3	tag_id_3_1042	Aps_1042_R3_F	CGACAGGTTCAGAGTTCTACAGTCCGACGATCAAAAAGACAGCAGATGCAAT		
		Aps_1042_R3_R	GTGACTGGAGTTCAGACGTGTGCTCTTCCGATCTAAGCATAAAACGGGCTATCT		
4	tag_id_3_1064	Aps_1064_R3_F	CGACAGGTTCAGAGTTCTACAGTCCGACGATCTGTTTCATCCTCTTAGCAGTT		
		Aps_1064_R3_R	GTGACTGGAGTTCAGACGTGTGCTCTTCCGATCTCAAACAAGGAATTTGACTCC		
5	tag_id_3_10857	Aps_10857_R3_F	CGACAGGTTCAGAGTTCTACAGTCCGACGATCGATAGCATCTCCACATCCTG		
		Aps_10857_R3_R	GTGACTGGAGTTCAGACGTGTGCTCTTCCGATCTTCTTGATCATTTCACCATCA		
6	tag_id_1186	Aps_1186_AO2_F	CGACAGGTTCAGAGTTCTACAGTCCGACGATCACAAGGACATGTCTGTTCTG	1:2	
		Aps_1186_AO2_R	GTGACTGGAGTTCAGACGTGTGCTCTTCCGATCTATTGTAAGTGGGCCAGGTTG	1:2	
7	tag_id_11930	Aps_11930_AN2_F	CGACAGGTTCAGAGTTCTACAGTCCGACGATCAGGGAGTGCTTGAAGTCTCG	1:2	
		Aps_11930_AN2_R	GTGACTGGAGTTCAGACGTGTGCTCTTCCGATCTTCTCCTGTTCCAGGTCCATT	1:2	
8	tag_id_12136	Aps_12136_AN_F	CGACAGGTTCAGAGTTCTACAGTCCGACGATCTCCAGCCAATCAGACAACAG	1:4	Removed
		Aps_12136_AN_R	GTGACTGGAGTTCAGACGTGTGCTCTTCCGATCTAGTACTCTGAAGCGGGGAGA	1:4	
9	tag_id_3_1251	Aps_1251_R3_F	CGACAGGTTCAGAGTTCTACAGTCCGACGATCCTTCAGTGGAAGGTGGAAT	1.5x	
		Aps_1251_R3_R	GTGACTGGAGTTCAGACGTGTGCTCTTCCGATCTTCATTGTTATATCAGGATAAATGTG	1.5x	
10	tag_id_1279	Aps_1279_AO2_F	CGACAGGTTCAGAGTTCTACAGTCCGACGATCTGCTGCCTTCAATATTTTCCA		
		Aps_1279_AO2_R	GTGACTGGAGTTCAGACGTGTGCTCTTCCGATCTCAGTGATGACCGATGCAGTT		
11	tag_id_13449	Aps_13449_AN2_F	CGACAGGTTCAGAGTTCTACAGTCCGACGATCAACCCCTGCAGTTAAAGCAA		
		Aps_13449_AN2_R	GTGACTGGAGTTCAGACGTGTGCTCTTCCGATCTAGCCTTGGCTCTGTGTTGTT		
12	tag_id_13755	Aps_13755_AN_F	CGACAGGTTCAGAGTTCTACAGTCCGACGATCATGTAGGCAGCCATCTTTGC	1:4	
		Aps_13755_AN_R	GTGACTGGAGTTCAGACGTGTGCTCTTCCGATCTTAGCCAAGTCAGGAGGAGGA	1:4	
13	tag_id_14470	Aps_14470_AN2_F	CGACAGGTTCAGAGTTCTACAGTCCGACGATCTGGCCAAAAAGCTCAATTTT		
		Aps_14470_AN2_R	GTGACTGGAGTTCAGACGTGTGCTCTTCCGATCTGATTCCACCGTTGTGTCCTT		
14	tag_id_1522	Aps_1522_F	CGACAGGTTCAGAGTTCTACAGTCCGACGATCGGAGAACTTGGTACTCGGGG	1:4	
		Aps_1522_R	GTGACTGGAGTTCAGACGTGTGCTCTTCCGATCTTTTTAACGCGGGTTCTGCTG	1:4	
15	tag_id_15263	Aps_15263_AN2_F	CGACAGGTTCAGAGTTCTACAGTCCGACGATCACAAGGCCGTTTTCTGTCTG		
		Aps_15263_AN2_R	GTGACTGGAGTTCAGACGTGTGCTCTTCCGATCTTGTTTTCTCCATTGTCAACATGA		
16	tag_id_15446	Aps_15446_AN2_F	CGACAGGTTCAGAGTTCTACAGTCCGACGATCCATTCTCTATCGATTTGACGCTTA		
		Aps_15446_AN2_R	GTGACTGGAGTTCAGACGTGTGCTCTTCCGATCTCGTCTGACGCAGTCAAGTG		
17	tag_id_16021	Aps_16021_AN_F	CGACAGGTTCAGAGTTCTACAGTCCGACGATCTTCCACACAATTATTAATGGCATA	1.5x	
		Aps_16021_AN_R	GTGACTGGAGTTCAGACGTGTGCTCTTCCGATCTTGTTGGGGATAATACTCCTGA	1.5x	
18	tag_id_3_1617	Aps_1617_R3_F	CGACAGGTTCAGAGTTCTACAGTCCGACGATCGGGGGTGTTTAAAGAAACTAA		
		Aps_1617_R3_R	GTGACTGGAGTTCAGACGTGTGCTCTTCCGATCTCATAGTTCTCGGCAATCAAG		
19	tag_id_16317	Aps_16317_AN2_F	CGACAGGTTCAGAGTTCTACAGTCCGACGATCGGGCTTTAGACAATGGCAGA		
		Aps_16317_AN2_R	GTGACTGGAGTTCAGACGTGTGCTCTTCCGATCTTCGAGCACATTCAGTACATGTTT		
20	tag_id_16387	Aps_16387_AN2_F	CGACAGGTTCAGAGTTCTACAGTCCGACGATCAACCTTCAGAGTTCGTCACC	1:2	
		Aps_16387_AN2_R	GTGACTGGAGTTCAGACGTGTGCTCTTCCGATCTTGCCAGAATGTGTGTGTGTG	1:2	
21	tag_id_16405	Aps_16405_AN2_F	CGACAGGTTCAGAGTTCTACAGTCCGACGATCCAGACTTCCACTGCCACTGA	1:2	
		Aps_16405_AN2_R	GTGACTGGAGTTCAGACGTGTGCTCTTCCGATCTGGACCAAGTTTCACTTCACCA	1:2	
22	tag_id_3_1666	Aps_1666_R3_F	CGACAGGTTCAGAGTTCTACAGTCCGACGATCTGTAAATTATGAACAATACACAACA		
		Aps_1666_R3_R	GTGACTGGAGTTCAGACGTGTGCTCTTCCGATCTCATGTTTCAACCCAGAATGT		
23	tag_id_16781	Aps_16781_AN_F	CGACAGGTTCAGAGTTCTACAGTCCGACGATCTCCGCTGTATTCCCACTGAT	1:2	
		Aps_16781_AN_R	GTGACTGGAGTTCAGACGTGTGCTCTTCCGATCTAAGCTCTCAGCTCGTTGACC	1:2	
24	tag_id_3_1682	Aps_1682_R3_F	CGACAGGTTCAGAGTTCTACAGTCCGACGATCGCATATTTGAAATCTTCCTCTC	1:2	
		Aps_1682_R3_R	GTGACTGGAGTTCAGACGTGTGCTCTTCCGATCTCATATGCAGCCTCAGACATA	1:2	
25	tag_id_3_1718	Aps_1718_R3_F	CGACAGGTTCAGAGTTCTACAGTCCGACGATCTATCGTGCATCCCTATTAGC	1:2	
		Aps_1718_R3_R	GTGACTGGAGTTCAGACGTGTGCTCTTCCGATCTAGGTTTGGTTGACAGCATAA	1:2	
26	tag_id_17490	Aps_17490_AN2_F	CGACAGGTTCAGAGTTCTACAGTCCGACGATCTCAATCAGCTATGTGCCCTCT	1:2	
		Aps_17490_AN2_R	GTGACTGGAGTTCAGACGTGTGCTCTTCCGATCTAAGGGGCTAATCACCATCCT	1:2	
27	tag_id_17512	Aps_17512_AN_F	CGACAGGTTCAGAGTTCTACAGTCCGACGATCTTGACCCAGCAAAGAGAGAAA		
		Aps_17512_AN_R	GTGACTGGAGTTCAGACGTGTGCTCTTCCGATCTTCCAGTGCACTTACATTCCAA		
28	tag_id_17566	Aps_17566_AN_F	CGACAGGTTCAGAGTTCTACAGTCCGACGATCTCAAGACACAGGAGAGGGTTTT	1:2	
		Aps_17566_AN_R	GTGACTGGAGTTCAGACGTGTGCTCTTCCGATCTCACACCTCAGCTTTCCCTCT	1:2	
29	tag_id_3_1784	Aps_1784_R3_F	CGACAGGTTCAGAGTTCTACAGTCCGACGATCTGCATACCTGGTTATGAATC		
		Aps_1784_R3_R	GTGACTGGAGTTCAGACGTGTGCTCTTCCGATCTCAGCCTAACAGACTCCTCAG		
30	tag_id_188	Aps_188_AN_F	CGACAGGTTCAGAGTTCTACAGTCCGACGATCTCTTTCCATAGCCTACTCTACCC		
		Aps_188_AN_R	GTGACTGGAGTTCAGACGTGTGCTCTTCCGATCTCGTTTTTGCCGTCTTTCTGT		
31	tag_id_1889	Aps_1889_AN2_F	CGACAGGTTCAGAGTTCTACAGTCCGACGATCTTCGGCTGCAGACATGTTAT	1:2	
		Aps_1889_AN2_R	GTGACTGGAGTTCAGACGTGTGCTCTTCCGATCTCTTCTGTCTGGGTGATGAACTG	1:2	
32	tag_id_3_1903	Aps_1903_R3_F	CGACAGGTTCAGAGTTCTACAGTCCGACGATCTACAATCTGACAGCATGGAG		
		Aps_1903_R3_R	GTGACTGGAGTTCAGACGTGTGCTCTTCCGATCTGCAAAGCAATGAAAAGAATG		

33	tag_id_3_2021	Aps_2021_R3_F	CGACAGGTTCAGAGTTCTACAGTCCGACGATCGAGTTTAATGTGGCCAACTC	1:2	
		Aps_2021_R3_R	GTGACTGGAGTTCAGACGTGTGCTCTTCCGATCTTTGTGCATTGTTGTTTATGG	1:2	
34	tag_id_2075	Aps_2075_F	CGACAGGTTCAGAGTTCTACAGTCCGACGATCAACACCAAATATACTAGATCACCTGT	1:2	
		Aps_2075_R	GTGACTGGAGTTCAGACGTGTGCTCTTCCGATCTCGGCGGATATTACACATGGC	1:2	
35	tag_id_3_2100	Aps_2100_R3_F	CGACAGGTTCAGAGTTCTACAGTCCGACGATCTCTAGGATTCTCGTCTTTACC	1:2	
		Aps_2100_R3_R	GTGACTGGAGTTCAGACGTGTGCTCTTCCGATCTTGTGCTGTTGTTACTGGTGT	1:2	
36	tag_id_2148	Aps_2148_AN2_F	CGACAGGTTCAGAGTTCTACAGTCCGACGATCGGGGGAGTGGAAGAGTTACC		
		Aps_2148_AN2_R	GTGACTGGAGTTCAGACGTGTGCTCTTCCGATCTTGAAGAGAAAGAAGCCCAAA		
37	tag_id_3_2167	Aps_2167_R3_F	CGACAGGTTCAGAGTTCTACAGTCCGACGATCGGACATAAAAAGTGGCAGTG	1:2	
		Aps_2167_R3_R	GTGACTGGAGTTCAGACGTGTGCTCTTCCGATCTAATTTCACAGGAGGTCACAG	1:2	
38	tag_id_3_219	Aps_219_R3_F	CGACAGGTTCAGAGTTCTACAGTCCGACGATCTGAGCTTTGTTTGAATTGTG	1.5x	
		Aps_219_R3_R	GTGACTGGAGTTCAGACGTGTGCTCTTCCGATCTGATTTGGTTGTTTGCTTTTG	1.5x	
39	tag_id_2195	Aps_2195_AN2_F	CGACAGGTTCAGAGTTCTACAGTCCGACGATCTCTTGTTTATTCAGAGCGTCACA	1:2	
		Aps_2195_AN2_R	GTGACTGGAGTTCAGACGTGTGCTCTTCCGATCTACTAGCGAAAATCGCTGCAT	1:2	
40	tag_id_2302	Aps_2302_F	CGACAGGTTCAGAGTTCTACAGTCCGACGATCAATCTGACCCAACCCTGCC		
		Aps_2302_R	GTGACTGGAGTTCAGACGTGTGCTCTTCCGATCTTGCATGATGTTATTTTTGAAAACGG		
41	tag_id_3_2345	Aps_2345_R3_F	CGACAGGTTCAGAGTTCTACAGTCCGACGATCAACATTAGCATCTCAAGTGG	1:2	
		Aps_2345_R3_R	GTGACTGGAGTTCAGACGTGTGCTCTTCCGATCTAAGACCTGGCAGTCTATGG	1:2	
42	tag_id_3_2358	Aps_2358_R3_F	CGACAGGTTCAGAGTTCTACAGTCCGACGATCGTGCTACGTTTCTTTTACGG		
		Aps_2358_R3_R	GTGACTGGAGTTCAGACGTGTGCTCTTCCGATCTGCTTCCATATCGAACTCAAG		
43	tag_id_2384	Aps_2384_AN2_F	CGACAGGTTCAGAGTTCTACAGTCCGACGATCGGCAGCTTGTTTTGGTTCAT		
		Aps_2384_AN2_R	GTGACTGGAGTTCAGACGTGTGCTCTTCCGATCTTATGAAACGGCAGTGAGCAG		
44	tag_id_3_2639	Aps_2639_R3_F	CGACAGGTTCAGAGTTCTACAGTCCGACGATCCAGATAACGTTTCGACCTTG		
		Aps_2639_R3_R	GTGACTGGAGTTCAGACGTGTGCTCTTCCGATCTCCAATAAGTGCCCAAAAATA		
45	tag_id_264	Aps_264_AN_F	CGACAGGTTCAGAGTTCTACAGTCCGACGATCGCTACGCCTCTCAGGACAGA	1:2	
		Aps_264_AN_R	GTGACTGGAGTTCAGACGTGTGCTCTTCCGATCTCCTTCTCCACGTTCCCTTATC	1:2	
46	tag_id_3_2646	Aps_2646_R3_F	CGACAGGTTCAGAGTTCTACAGTCCGACGATCGATCTCCTTATTTGGGCAAT		
		Aps_2646_R3_R	GTGACTGGAGTTCAGACGTGTGCTCTTCCGATCTTTGTTTGACATTGAGAATGC		
47	tag_id_3_2704	Aps_2704_R3_F	CGACAGGTTCAGAGTTCTACAGTCCGACGATCCAATTCTGAGGGAACTGATG	1:2	
		Aps_2704_R3_R	GTGACTGGAGTTCAGACGTGTGCTCTTCCGATCTAATTCTTACACATGGCACCT	1:2	
48	tag_id_3_2822	Aps_2822_R3_F	CGACAGGTTCAGAGTTCTACAGTCCGACGATCGGCATGACCATCACTTTATC		
		Aps_2822_R3_R	GTGACTGGAGTTCAGACGTGTGCTCTTCCGATCTGGAAAACCTGTGATGTATGG		
49	tag_id_3050	Aps_3050_AN2_F	CGACAGGTTCAGAGTTCTACAGTCCGACGATCACCCAAAACCAAAACATCCA		Removed
		Aps_3050_AN2_R	GTGACTGGAGTTCAGACGTGTGCTCTTCCGATCTCCAAGTTGTGATCATTTGCAG		
50	tag_id_3_3229	Aps_3229_R3_F	CGACAGGTTCAGAGTTCTACAGTCCGACGATCTTGTTATGTGTCCTTCCACA		
		Aps_3229_R3_R	GTGACTGGAGTTCAGACGTGTGCTCTTCCGATCTCATGCAGAACTGAGACACAT		
51	tag_id_3_3243	Aps_3243_R3_F	CGACAGGTTCAGAGTTCTACAGTCCGACGATCTCTATGGGGAAAATCTCTGA		
		Aps_3243_R3_R	GTGACTGGAGTTCAGACGTGTGCTCTTCCGATCTTCACAGTGCTGGGTATGTAA		
52	tag_id_3296	Aps_3296_AN_F	CGACAGGTTCAGAGTTCTACAGTCCGACGATCCCGTAGGGCCTAGGATGAGT		
		Aps_3296_AN_R	GTGACTGGAGTTCAGACGTGTGCTCTTCCGATCTCCTCGGTTTTTGCTTTTTCA		
53	tag_id_3_336	Aps_336_R3_F	CGACAGGTTCAGAGTTCTACAGTCCGACGATCACGCAGCTTATCTTCATTTT		
		Aps_336_R3_R	GTGACTGGAGTTCAGACGTGTGCTCTTCCGATCTTTCAAGATTAATTAGAGCTAGGC		
54	tag_id_3385	Aps_3385_AN_F	CGACAGGTTCAGAGTTCTACAGTCCGACGATCCTGAATGGATGAATGAACGA		
		Aps_3385_AN_R	GTGACTGGAGTTCAGACGTGTGCTCTTCCGATCTTCCAGACACACATCCTTCCA		
55	tag_id_3462	Aps_3462_AN_F	CGACAGGTTCAGAGTTCTACAGTCCGACGATCAGGCTAACCGTGTAGACCTGA	1:2	
		Aps_3462_AN_R	GTGACTGGAGTTCAGACGTGTGCTCTTCCGATCTGCATGGTAAACACCGCAGTA	1:2	
56	tag_id_3583	Aps_3583_AN2_F	CGACAGGTTCAGAGTTCTACAGTCCGACGATCGCCCAGCTCTGTGCTTAAAA		
		Aps_3583_AN2_R	GTGACTGGAGTTCAGACGTGTGCTCTTCCGATCTAATCTGGGGCAATCAGTCC		
57	tag_id_3_3609	Aps_3609_R3_F	CGACAGGTTCAGAGTTCTACAGTCCGACGATCGAGGCGTGACTGCTATAGAT		
		Aps_3609_R3_R	GTGACTGGAGTTCAGACGTGTGCTCTTCCGATCTGGAAAGAAGAAAGCGCTAA		
58	tag_id_3651	Aps_3651_AO_F	CGACAGGTTCAGAGTTCTACAGTCCGACGATCCGTGGTCCATTTCCTGGTT	1:2	
		Aps_3651_AO_R	GTGACTGGAGTTCAGACGTGTGCTCTTCCGATCTGCTACCTCTTAACATTGCCTCA	1:2	
59	tag_id_3694	Aps_3694_AN_F	CGACAGGTTCAGAGTTCTACAGTCCGACGATCCAGGGGCCAGCTGATTATTA		
		Aps_3694_AN_R	GTGACTGGAGTTCAGACGTGTGCTCTTCCGATCTCCAAGCCCCTTGGAAATC		
60	tag_id_3_3734	Aps_3734_R3_F	CGACAGGTTCAGAGTTCTACAGTCCGACGATCCGAGAAGGACAAGGAGAAG	1:2	
		Aps_3734_R3_R	GTGACTGGAGTTCAGACGTGTGCTCTTCCGATCTATTTCAGAAAACGAGGATGA	1:2	
61	tag_id_3798	Aps_3798_AN2_F	CGACAGGTTCAGAGTTCTACAGTCCGACGATCTGGATTCCACCCACTGTTTT	1:2	
		Aps_3798_AN2_R	GTGACTGGAGTTCAGACGTGTGCTCTTCCGATCTTTCGTCCAAAGACACGACTG	1:2	
62	tag_id_3859	Aps_3859_AN2_F	CGACAGGTTCAGAGTTCTACAGTCCGACGATCTTCTAATGGGCTGTTTTAGTG		
		Aps_3859_AN2_R	GTGACTGGAGTTCAGACGTGTGCTCTTCCGATCTCGCCGTGAGGATAACTGAAT		
63	tag_id_3_393	Aps_393_R3_F	CGACAGGTTCAGAGTTCTACAGTCCGACGATCGAGATTTTTCCCCTCTGC	1:2	
		Aps_393_R3_R	GTGACTGGAGTTCAGACGTGTGCTCTTCCGATCTGTCACATTCTGTTGCTGTTG	1:2	
64	tag_id_3_3940	Aps_3940_R3_F	CGACAGGTTCAGAGTTCTACAGTCCGACGATCGCACACTGACTCAGACACAC	1:2	
		Aps_3940_R3_R	GTGACTGGAGTTCAGACGTGTGCTCTTCCGATCTGCATCTTGTTTTCATGCTCT	1:2	
65	tag_id_3_4166	Aps_4166_R3_F	CGACAGGTTCAGAGTTCTACAGTCCGACGATCTTCTCTGGGAATCTTACCTT		
		Aps_4166_R3_R	GTGACTGGAGTTCAGACGTGTGCTCTTCCGATCTTGTAGCTCAGTCAAAGCAAA		
66	tag_id_3_4243	Aps_4243_R3_F	CGACAGGTTCAGAGTTCTACAGTCCGACGATCAATTCCCACAGGTATCCAA		

		Aps_4243_R3_R	GTGACTGGAGTTCAGACGTGTGCTCTTCCGATCTGAAGACAAAACAGTGGGCTA	
67	tag_id_4446	Aps_4446_AN_F	CGACAGGTTCAGAGTTCTACAGTCCGACGATCAGAACGGCTTGCCTTTGTC	
		Aps_4446_AN_R	GTGACTGGAGTTCAGACGTGTGCTCTTCCGATCTTCGTGCAATGACATCAACAA	
68	tag_id_3_4539	Aps_4539_R3_F	CGACAGGTTCAGAGTTCTACAGTCCGACGATCCTGTTAGGGCTGTATTTTGG	1:2
		Aps_4539_R3_R	GTGACTGGAGTTCAGACGTGTGCTCTTCCGATCTTTAATAGCCACACCCCTAAG	1:2
69	tag_id_4705	Aps_4705_AN_F	CGACAGGTTCAGAGTTCTACAGTCCGACGATCTGCGCAAAATAATTCAAGTTGT	
		Aps_4705_AN_R	GTGACTGGAGTTCAGACGTGTGCTCTTCCGATCTGCTCTTTGGAGCGGTAAGTG	
70	tag_id_478	Aps_478_AN2_F	CGACAGGTTCAGAGTTCTACAGTCCGACGATCTTCAAGGGTAACGCAAAGATT	
		Aps_478_AN2_R	GTGACTGGAGTTCAGACGTGTGCTCTTCCGATCTGCAAAAAGCCAACATCTATGA	
71	tag_id_3_481	Aps_481_R3_F	CGACAGGTTCAGAGTTCTACAGTCCGACGATCATCTCAGGTGTTTGTCCAGT	
		Aps_481_R3_R	GTGACTGGAGTTCAGACGTGTGCTCTTCCGATCTGGGGCTTTTGATCATCTTA	
72	tag_id_3_4984	Aps_4984_R3_F	CGACAGGTTCAGAGTTCTACAGTCCGACGATCACCTCATTTGGATGATCTCT	
		Aps_4984_R3_R	GTGACTGGAGTTCAGACGTGTGCTCTTCCGATCTCAGAACTTAATCCACCCAAG	
73	tag_id_3_504	Aps_504_R3_F	CGACAGGTTCAGAGTTCTACAGTCCGACGATCGGACCACCTCTACATCATTG	
		Aps_504_R3_R	GTGACTGGAGTTCAGACGTGTGCTCTTCCGATCTTGTGATATCATTACACGCAGT	
74	tag_id_3_5143	Aps_5143_R3_F	CGACAGGTTCAGAGTTCTACAGTCCGACGATCCAAGCACATGTTTAACTCCA	1.5x
		Aps_5143_R3_R	GTGACTGGAGTTCAGACGTGTGCTCTTCCGATCTAACGTATCCATAACACAGTGG	1.5x
75	tag_id_5174	Aps_5174_F	CGACAGGTTCAGAGTTCTACAGTCCGACGATCTTGCACGATTTTTCCTGTAGAAG	
		Aps_5174_R	GTGACTGGAGTTCAGACGTGTGCTCTTCCGATCTACCGGAATTTTTCTTTAAGCGCA	
76	tag_id_5207	Aps_5207_AN_F	CGACAGGTTCAGAGTTCTACAGTCCGACGATCCCTGGAGGCCATCTTGTATT	
		Aps_5207_AN_R	GTGACTGGAGTTCAGACGTGTGCTCTTCCGATCTACCTCGTCCACCAGTGTTGT	
77	tag_id_5366	Aps_5366_F	CGACAGGTTCAGAGTTCTACAGTCCGACGATCTTCTCATCCACTGCCATGCA	1:2
		Aps_5366_R	GTGACTGGAGTTCAGACGTGTGCTCTTCCGATCTTCTTGCACTGACAAGAGGGG	1:2
78	tag_id_5474	Aps_5474_AN2_F	CGACAGGTTCAGAGTTCTACAGTCCGACGATCTAACACAGCCTCAGCAATCG	1:2
		Aps_5474_AN2_R	GTGACTGGAGTTCAGACGTGTGCTCTTCCGATCTGTAGGCAGAGCCAGGTATGG	1:2
79	tag_id_3_5502	Aps_5502_R3_F	CGACAGGTTCAGAGTTCTACAGTCCGACGATCGAAACAGAGTTCCCGTCAT	
		Aps_5502_R3_R	GTGACTGGAGTTCAGACGTGTGCTCTTCCGATCTCAGGGAAAACTGGGATACTT	
80	tag_id_5669	Aps_5669_AO_F	CGACAGGTTCAGAGTTCTACAGTCCGACGATCGCCATCACCGTAGAATGAGG	1:2
		Aps_5669_AO_R	GTGACTGGAGTTCAGACGTGTGCTCTTCCGATCTCAGTTTTCCTCCACGTCACA	1:2
81	tag_id_3_5833	Aps_5833_R3_F	CGACAGGTTCAGAGTTCTACAGTCCGACGATCGAAAAGCACTTGTCTGCTCT	
		Aps_5833_R3_R	GTGACTGGAGTTCAGACGTGTGCTCTTCCGATCTCCCGTAACATGGTATAATGG	
82	tag_id_5945	Aps_5945_AN_F	CGACAGGTTCAGAGTTCTACAGTCCGACGATCGGTTCCAAAGGAAGGAAGGA	
		Aps_5945_AN_R	GTGACTGGAGTTCAGACGTGTGCTCTTCCGATCTGCAATTTCCCTTTAGTGATGG	
83	tag_id_3_6135	Aps_6135_R3_F	CGACAGGTTCAGAGTTCTACAGTCCGACGATCGCTTTAGGTTCAGCTCTGG	1:2
		Aps_6135_R3_R	GTGACTGGAGTTCAGACGTGTGCTCTTCCGATCTTGATGAACAGGAAGTGCAT	1:2
84	tag_id_3_6183	Aps_6183_R3_F	CGACAGGTTCAGAGTTCTACAGTCCGACGATCCTGTGTATAAGGGATGCTCA	1:2
		Aps_6183_R3_R	GTGACTGGAGTTCAGACGTGTGCTCTTCCGATCTGAACCCGGACCCGAAAGT	1:2
85	tag_id_6448	Aps_6448_AN_F	CGACAGGTTCAGAGTTCTACAGTCCGACGATCGACATGCCCCCAAAGTGTAG	
		Aps_6448_AN_R	GTGACTGGAGTTCAGACGTGTGCTCTTCCGATCTTGTGTGAGCTTGTTTTCAAAGG	
86	tag_id_3_647	Aps_647_R3_F	CGACAGGTTCAGAGTTCTACAGTCCGACGATCCTCCACTAATTGCTGACCAT	
		Aps_647_R3_R	GTGACTGGAGTTCAGACGTGTGCTCTTCCGATCTCCTGATAGACTGGACTTGTCA	
87	tag_id_6592	Aps_6592_AN_F	CGACAGGTTCAGAGTTCTACAGTCCGACGATCAAAATGATCTGATATAGGACATGAGC	
		Aps_6592_AN_R	GTGACTGGAGTTCAGACGTGTGCTCTTCCGATCTTGAATAGGCCTCCAAAACAAA	
88	tag_id_3_6676	Aps_6676_R3_F	CGACAGGTTCAGAGTTCTACAGTCCGACGATCGTTTACCAGGATACTCAGAGGT	
		Aps_6676_R3_R	GTGACTGGAGTTCAGACGTGTGCTCTTCCGATCTACAGGGAACAGGATGAATTA	
89	tag_id_3_6775	Aps_6775_R3_F	CGACAGGTTCAGAGTTCTACAGTCCGACGATCCTGTTTTTCAAAAGGCTCAG	
		Aps_6775_R3_R	GTGACTGGAGTTCAGACGTGTGCTCTTCCGATCTAGACATAGAGAAATCAAGAGAGG	
90	tag_id_678	Aps_678_AN_F	CGACAGGTTCAGAGTTCTACAGTCCGACGATCATGCTTTCTGCAGCATGTGA	1:2
		Aps_678_AN_R	GTGACTGGAGTTCAGACGTGTGCTCTTCCGATCTGTAGAAACCGTGCACCCGTA	1:2
91	tag_id_6887	Aps_6887_AB_F	CGACAGGTTCAGAGTTCTACAGTCCGACGATCCCTGCCTTGGTTAGAACAGC	1:2
		Aps_6887_AB_R	GTGACTGGAGTTCAGACGTGTGCTCTTCCGATCTCGTTCGTGAGCCAATAAAGC	1:2
92	tag_id_3_707	Aps_707_R3_F	CGACAGGTTCAGAGTTCTACAGTCCGACGATCGTTGATCCGAAACAGAAATC	1.5x
		Aps_707_R3_R	GTGACTGGAGTTCAGACGTGTGCTCTTCCGATCTTGTTTATGTGTGAACTGAATGA	1.5x
93	tag_id_3_7088	Aps_7088_R3_F	CGACAGGTTCAGAGTTCTACAGTCCGACGATCCCCATGTAAACATACCGTAGA	
		Aps_7088_R3_R	GTGACTGGAGTTCAGACGTGTGCTCTTCCGATCTTGTGTATCCCACAACACCT	
94	tag_id_3_7165	Aps_7165_R3_F	CGACAGGTTCAGAGTTCTACAGTCCGACGATCAATTCTGTTTCAAAGCCAAG	
		Aps_7165_R3_R	GTGACTGGAGTTCAGACGTGTGCTCTTCCGATCTAAATAAAATGCCATGTCACC	
95	tag_id_7285	Aps_7285_AO2_F	CGACAGGTTCAGAGTTCTACAGTCCGACGATCCCTCGCTTCCTGTTTTGACA	
		Aps_7285_AO2_R	GTGACTGGAGTTCAGACGTGTGCTCTTCCGATCTGTCACACAGGGGCAAACATC	
96	tag_id_3_7300	Aps_7300_R3_F	CGACAGGTTCAGAGTTCTACAGTCCGACGATCCAACTAAAATGCAAACACACA	1:2
		Aps_7300_R3_R	GTGACTGGAGTTCAGACGTGTGCTCTTCCGATCTGTTGCAGAACTGTAGCCTTC	1:2
97	tag_id_7605	Aps_7605_AN_F	CGACAGGTTCAGAGTTCTACAGTCCGACGATCCCTTGAATTGTTGTGGAACG	
		Aps_7605_AN_R	GTGACTGGAGTTCAGACGTGTGCTCTTCCGATCTCCGACAGCTATTAGGGACTGA	
98	tag_id_7637	Aps_7637_AN_F	CGACAGGTTCAGAGTTCTACAGTCCGACGATCGCATGTCCTGTCCTGGTTCT	1:2
		Aps_7637_AN_R	GTGACTGGAGTTCAGACGTGTGCTCTTCCGATCTACTCACAGCTCTGCCGAATG	1:2
99	tag_id_3_7860	Aps_7860_R3_F	CGACAGGTTCAGAGTTCTACAGTCCGACGATCTAGCATTACCATGATGACCA	1:2
		Aps_7860_R3_R	GTGACTGGAGTTCAGACGTGTGCTCTTCCGATCTATTTAGGCAGTGTGGAGCTA	1:2

100	tag_id_3_7947	Aps_7947_R3_F	CGACAGGTTCAGAGTTCTACAGTCCGACGATCCCCTTAGAGAATGTGAAGACC	1:2	
		Aps_7947_R3_R	GTGACTGGAGTTCAGACGTGTGCTCTTCCGATCTGTTGGTTCTGGTGTCAGTG	1:2	
101	tag_id_3_8117	Aps_8117_R3_F	CGACAGGTTCAGAGTTCTACAGTCCGACGATCGTATGCTCACCGCTGCTC	1:2	
		Aps_8117_R3_R	GTGACTGGAGTTCAGACGTGTGCTCTTCCGATCTGCTCTCTCGTGTTGTCTTCT	1:2	
102	tag_id_3_8138	Aps_8138_R3_F	CGACAGGTTCAGAGTTCTACAGTCCGACGATCACCTTGCCTCATAAATGACT		
		Aps_8138_R3_R	GTGACTGGAGTTCAGACGTGTGCTCTTCCGATCTTACCGTAGAGTTCCAGTTCC		
103	tag_id_3_872	Aps_872_R3_F	CGACAGGTTCAGAGTTCTACAGTCCGACGATCCCCAAAGAAGACATTCAGAT		
		Aps_872_R3_R	GTGACTGGAGTTCAGACGTGTGCTCTTCCGATCTTATTAAGCCGGCATAGGGTA		
104	tag_id_8754	Aps_8754_AN2_F	CGACAGGTTCAGAGTTCTACAGTCCGACGATCTGTGATTATTCTGCCAGTGCT	1:2	
		Aps_8754_AN2_R	GTGACTGGAGTTCAGACGTGTGCTCTTCCGATCTCCGGAGCAACAGACAAGC	1:2	
105	tag_id_8966	Aps_8966_AN_F	CGACAGGTTCAGAGTTCTACAGTCCGACGATCCTACAAGGCCTGTGGGAAAC	1:2	
		Aps_8966_AN_R	GTGACTGGAGTTCAGACGTGTGCTCTTCCGATCTGCTACAGAATGACGCACTCC	1:2	
106	tag_id_9014	Aps_9014_AO_F	CGACAGGTTCAGAGTTCTACAGTCCGACGATCACACCTCCCACCCTGTGTAA	1:2	
		Aps_9014_AO_R	GTGACTGGAGTTCAGACGTGTGCTCTTCCGATCTGATGGACCATGCTGCTTCTA	1:2	
107	tag_id_3_9267	Aps_9267_R3_F	CGACAGGTTCAGAGTTCTACAGTCCGACGATCTGGTTTGGTTGAAAACTTGT	1.5x	
		Aps_9267_R3_R	GTGACTGGAGTTCAGACGTGTGCTCTTCCGATCTACCAACAGAACCATTTGAAG	1.5x	
108	tag_id_3_9448	Aps_9448_R3_F	CGACAGGTTCAGAGTTCTACAGTCCGACGATCGCTTGCACTCCTAGAACATT		Removed
		Aps_9448_R3_R	GTGACTGGAGTTCAGACGTGTGCTCTTCCGATCTGTGAGGTCAATCTCTGCAAT		
109	tag_id_3_9449	Aps_9449_R3_F	CGACAGGTTCAGAGTTCTACAGTCCGACGATCGTCATGGACAAAAACATCAA		
		Aps_9449_R3_R	GTGACTGGAGTTCAGACGTGTGCTCTTCCGATCTGCCTTCATTTTGGAGGTAG		
110	tag_id_3_945	Aps_945_R3_F	CGACAGGTTCAGAGTTCTACAGTCCGACGATCGCGGCTTTGTTTTCTTCT		
		Aps_945_R3_R	GTGACTGGAGTTCAGACGTGTGCTCTTCCGATCTAACATGTGGCCAACTTTCT		
111	tag_id_9493	Aps_9493_AN_F	CGACAGGTTCAGAGTTCTACAGTCCGACGATCAAGCCGGCCTTGCAG		
		Aps_9493_AN_R	GTGACTGGAGTTCAGACGTGTGCTCTTCCGATCTGCGCCGAGAGCACCT		
112	tag_id_9542	Aps_9542_AN_F	CGACAGGTTCAGAGTTCTACAGTCCGACGATCGGGGGTGATTATGGGATTCT	1:2	
		Aps_9542_AN_R	GTGACTGGAGTTCAGACGTGTGCTCTTCCGATCTTGGTGTTAACATACCACAGAGAGA	1:2	
113	tag_id_958	Aps_958_AN_F	CGACAGGTTCAGAGTTCTACAGTCCGACGATCACAAACGTCTCGGACCACTC	1:2	
		Aps_958_AN_R	GTGACTGGAGTTCAGACGTGTGCTCTTCCGATCTGATCTGTGCAGCCATTTGAG	1:2	
114	tag_id_9584	Aps_9584_AN2_F	CGACAGGTTCAGAGTTCTACAGTCCGACGATCACTTGCAATTAAGCCCCTCA	1:4	
		Aps_9584_AN2_R	GTGACTGGAGTTCAGACGTGTGCTCTTCCGATCTCACAAGAGGGGTATGGCAGA	1:4	
115	tag_id_3_990	Aps_990_R3_F	CGACAGGTTCAGAGTTCTACAGTCCGACGATCGCAAGGAGACCAACAAAGTA		
		Aps_990_R3_R	GTGACTGGAGTTCAGACGTGTGCTCTTCCGATCTTCACAGTGGCCAATAAGATT		
116	tag_id_3_996	Aps_996_R3_F	CGACAGGTTCAGAGTTCTACAGTCCGACGATCAAAACAATAATGATAGACACAGG	1.5x	
		Aps_996_R3_R	GTGACTGGAGTTCAGACGTGTGCTCTTCCGATCTAGCAAAACCTGGAATATTTG	1.5x	
117	tag_id_3_9982	Aps_9982_R3_F	CGACAGGTTCAGAGTTCTACAGTCCGACGATCTCCCTCAAGTCTGTCCTAAC		
		Aps_9982_R3_R	GTGACTGGAGTTCAGACGTGTGCTCTTCCGATCTGCATCTGTACCCGTCAATTA		

Supplementary Table S2 Matrix of posterior assignments with newhybrids based on simulations for pure and hybrid offspring

NewHybrids	ANAD	A_BC	F1	F2	L_BC	LAND
**ANAD**	**1.00**					
**ANAD_BC**	0.03	**0.94**		0.03		
**F1**			**0.98**	0.01	0.01	
**F2**		0.02	0.02	**0.94**	0.02	
**LAND_BC**				0.01	**0.99**	
**LAND**						**1.00**

Supplementary Table S3 Posterior probability assignment and parentage of juveniles for 2017

		Structure		NewHybrids								FRANz											
Group	Individual	ANAD	LAND	ANAD	LAND	F1	F2	A_BC	L_BC	Assignment	Habitat	Parent1	Sex	Stocking date	Length	LOD	Post Prob	Parent2	Sex	Stocking date	Length	LOD	Post Prob
Juvenile	YOY_AP019414	0.30	0.70	0.00	0.00	0.00	0.01	0.00	0.99	LAND_BC	P												
Juvenile	YOY_AP019415	0.00	1.00	0.00	1.00	0.00	0.00	0.00	0.00	LAND	P												
Juvenile	YOY_AP019416	0.00	1.00	0.00	1.00	0.00	0.00	0.00	0.00	LAND	P												
Juvenile	YOY_AP019417	0.00	1.00	0.00	1.00	0.00	0.00	0.00	0.00	LAND	P												
Juvenile	YOY_AP019418	0.00	1.00	0.00	1.00	0.00	0.00	0.00	0.00	LAND	P												
Juvenile	YOY_AP019419	0.00	1.00	0.00	1.00	0.00	0.00	0.00	0.00	LAND	P												
Juvenile	YOY_AP019420	0.00	1.00	0.00	1.00	0.00	0.00	0.00	0.00	LAND	P												
Juvenile	YOY_AP019421	0.00	1.00	0.00	1.00	0.00	0.00	0.00	0.00	LAND	P												
Juvenile	YOY_AP019422	1.00	0.00	1.00	0.00	0.00	0.00	0.00	0.00	ANAD	P	BvAP018414	M	4/19/17	266	4.65E+01	1	BvAP017369	F	3/30/17	294	5.03E+01	1
Juvenile	YOY_AP019423	0.00	1.00	0.00	1.00	0.00	0.00	0.00	0.00	LAND	P												
Juvenile	YOY_AP019424	0.00	1.00	0.00	1.00	0.00	0.00	0.00	0.00	LAND	P												
Juvenile	YOY_AP019425	0.00	1.00	0.00	1.00	0.00	0.00	0.00	0.00	LAND	P												
Juvenile	YOY_AP019426	0.00	1.00	0.00	1.00	0.00	0.00	0.00	0.00	LAND	P												
Juvenile	YOY_AP019427	0.00	1.00	0.00	1.00	0.00	0.00	0.00	0.00	LAND	P												
Juvenile	YOY_AP019428	0.00	1.00	0.00	1.00	0.00	0.00	0.00	0.00	LAND	P												
Juvenile	YOY_AP019429	0.00	1.00	0.00	1.00	0.00	0.00	0.00	0.00	LAND	P												
Juvenile	YOY_AP019430	0.00	1.00	0.00	1.00	0.00	0.00	0.00	0.00	LAND	P												
Juvenile	YOY_AP019431	0.00	1.00	0.00	1.00	0.00	0.00	0.00	0.00	LAND	P												
Juvenile	YOY_AP019432	0.00	1.00	0.00	1.00	0.00	0.00	0.00	0.00	LAND	P												
Juvenile	YOY_AP019433	0.00	1.00	0.00	1.00	0.00	0.00	0.00	0.00	LAND	P												
Juvenile	YOY_AP019434	1.00	0.00	1.00	0.00	0.00	0.00	0.00	0.00	ANAD	P	BvAP017921	M	4/12/17	245	4.06E+01	1	BvAP017564	F	3/30/17	283	4.38E+01	1
Juvenile	YOY_AP019435	0.00	1.00	0.00	1.00	0.00	0.00	0.00	0.00	LAND	P												
Juvenile	YOY_AP019436	0.00	1.00	0.00	1.00	0.00	0.00	0.00	0.00	LAND	P												
Juvenile	YOY_AP019437	0.00	1.00	0.00	1.00	0.00	0.00	0.00	0.00	LAND	P												
Juvenile	YOY_AP019438	0.00	1.00	0.00	1.00	0.00	0.00	0.00	0.00	LAND	P												
Juvenile	YOY_AP019439	0.00	1.00	0.00	1.00	0.00	0.00	0.00	0.00	LAND	P												
Juvenile	YOY_AP019440	0.00	1.00	0.00	1.00	0.00	0.00	0.00	0.00	LAND	P												
Juvenile	YOY_AP019441	0.00	1.00	0.00	1.00	0.00	0.00	0.00	0.00	LAND	P												
Juvenile	YOY_AP019442	0.00	1.00	0.00	1.00	0.00	0.00	0.00	0.00	LAND	P												
Juvenile	YOY_AP019443	0.00	1.00	0.00	1.00	0.00	0.00	0.00	0.00	LAND	P												
Juvenile	YOY_AP019444	0.00	1.00	0.00	1.00	0.00	0.00	0.00	0.00	LAND	P												
Juvenile	YOY_AP019445	0.00	1.00	0.00	1.00	0.00	0.00	0.00	0.00	LAND	P												
Juvenile	YOY_AP019446	0.00	1.00	0.00	1.00	0.00	0.00	0.00	0.00	LAND	P												
Juvenile	YOY_AP019447	0.00	1.00	0.00	1.00	0.00	0.00	0.00	0.00	LAND	P												
Juvenile	YOY_AP019448	0.00	1.00	0.00	1.00	0.00	0.00	0.00	0.00	LAND	P												
Juvenile	YOY_AP019449	0.18	0.82	0.00	0.00	0.00	0.00	0.00	1.00	LAND_BC	P												
Juvenile	YOY_AP019451	1.00	0.00	1.00	0.00	0.00	0.00	0.00	0.00	ANAD	P	BvAP019142	M	4/12/17	266	4.45E+01	1	BvAP018323	F	4/19/17	261	4.96E+01	1
Juvenile	YOY_AP019452	0.00	1.00	0.00	1.00	0.00	0.00	0.00	0.00	LAND	P												
Juvenile	YOY_AP019453	0.00	1.00	0.00	1.00	0.00	0.00	0.00	0.00	LAND	P												
Juvenile	YOY_AP019454	0.00	1.00	0.00	1.00	0.00	0.00	0.00	0.00	LAND	P												
Juvenile	YOY_AP019455	0.00	1.00	0.00	1.00	0.00	0.00	0.00	0.00	LAND	P												
Juvenile	YOY_AP019456	0.00	1.00	0.00	1.00	0.00	0.00	0.00	0.00	LAND	P												
Juvenile	YOY_AP019457	0.00	1.00	0.00	1.00	0.00	0.00	0.00	0.00	LAND	P												
Juvenile	YOY_AP019458	0.00	1.00	0.00	1.00	0.00	0.00	0.00	0.00	LAND	P												
Juvenile	YOY_AP019459	0.00	1.00	0.00	1.00	0.00	0.00	0.00	0.00	LAND	P												
Juvenile	YOY_AP019460	0.00	1.00	0.00	1.00	0.00	0.00	0.00	0.00	LAND	P												
Juvenile	YOY_AP019461	0.00	1.00	0.00	1.00	0.00	0.00	0.00	0.00	LAND	P												
Juvenile	YOY_AP019462	0.00	1.00	0.00	1.00	0.00	0.00	0.00	0.00	LAND	P												
Juvenile	YOY_AP019463	0.00	1.00	0.00	1.00	0.00	0.00	0.00	0.00	LAND	P												
Juvenile	YOY_AP019464	0.00	1.00	0.00	1.00	0.00	0.00	0.00	0.00	LAND	P												
Juvenile	YOY_AP019465	0.00	1.00	0.00	1.00	0.00	0.00	0.00	0.00	LAND	P												
Juvenile	YOY_AP019466	0.00	1.00	0.00	1.00	0.00	0.00	0.00	0.00	LAND	P												
Juvenile	YOY_AP019467	0.00	1.00	0.00	1.00	0.00	0.00	0.00	0.00	LAND	P												
Juvenile	YOY_AP019468	0.00	1.00	0.00	1.00	0.00	0.00	0.00	0.00	LAND	P												
Juvenile	YOY_AP019469	1.00	0.00	1.00	0.00	0.00	0.00	0.00	0.00	ANAD	P	BvAP019017	M	4/12/17	255	4.98E+01	1	BvAP018601	F	4/12/17	263	3.69E+01	1
Juvenile	YOY_AP019470	0.00	1.00	0.00	1.00	0.00	0.00	0.00	0.00	LAND	P												
Juvenile	YOY_AP019471	0.00	1.00	0.00	1.00	0.00	0.00	0.00	0.00	LAND	P												
Juvenile	YOY_AP019472	0.00	1.00	0.00	1.00	0.00	0.00	0.00	0.00	LAND	P												
Juvenile	YOY_AP019473	0.00	1.00	0.00	1.00	0.00	0.00	0.00	0.00	LAND	P												
Juvenile	YOY_AP019474	0.00	1.00	0.00	1.00	0.00	0.00	0.00	0.00	LAND	P												
Juvenile	YOY_AP019475	0.00	1.00	0.00	1.00	0.00	0.00	0.00	0.00	LAND	P												
Juvenile	YOY_AP019476	0.00	1.00	0.00	1.00	0.00	0.00	0.00	0.00	LAND	P												
Juvenile	YOY_AP019477	0.00	1.00	0.00	1.00	0.00	0.00	0.00	0.00	LAND	P												
Juvenile	YOY_AP019478	0.00	1.00	0.00	1.00	0.00	0.00	0.00	0.00	LAND	P												
Juvenile	YOY_AP019479	0.00	1.00	0.00	1.00	0.00	0.00	0.00	0.00	LAND	P												
Juvenile	YOY_AP019481	1.00	0.00	1.00	0.00	0.00	0.00	0.00	0.00	ANAD	P	BvAP018631	M	4/12/17	256	4.72E+01	1	BvAP016916	F	3/30/17	277	5.47E+01	1
Juvenile	YOY_AP019482	0.00	1.00	0.00	1.00	0.00	0.00	0.00	0.00	LAND	P												
Juvenile	YOY_AP019483	0.00	1.00	0.00	1.00	0.00	0.00	0.00	0.00	LAND	P												
Juvenile	YOY_AP019484	0.00	1.00	0.00	1.00	0.00	0.00	0.00	0.00	LAND	P												
Juvenile	YOY_AP019485	0.00	1.00	0.00	1.00	0.00	0.00	0.00	0.00	LAND	P												
Juvenile	YOY_AP019486	0.00	1.00	0.00	1.00	0.00	0.00	0.00	0.00	LAND	P												
Juvenile	YOY_AP019487	0.00	1.00	0.00	1.00	0.00	0.00	0.00	0.00	LAND	P												
Juvenile	YOY_AP019488	0.00	1.00	0.00	1.00	0.00	0.00	0.00	0.00	LAND	P												
Juvenile	YOY_AP019489	0.00	1.00	0.00	1.00	0.00	0.00	0.00	0.00	LAND	P												
Juvenile	YOY_AP019490	0.00	1.00	0.00	1.00	0.00	0.00	0.00	0.00	LAND	P												
Juvenile	YOY_AP019491	0.00	1.00	0.00	1.00	0.00	0.00	0.00	0.00	LAND	P												
Juvenile	YOY_AP019492	0.00	1.00	0.00	1.00	0.00	0.00	0.00	0.00	LAND	P												
Juvenile	YOY_AP019493	0.00	1.00	0.00	1.00	0.00	0.00	0.00	0.00	LAND	P												
Juvenile	YOY_AP019494	0.00	1.00	0.00	1.00	0.00	0.00	0.00	0.00	LAND	P												
Juvenile	YOY_AP019495	0.00	1.00	0.00	1.00	0.00	0.00	0.00	0.00	LAND	P												
Juvenile	YOY_AP019496	0.00	1.00	0.00	1.00	0.00	0.00	0.00	0.00	LAND	P												
Juvenile	YOY_AP019497	0.00	1.00	0.00	1.00	0.00	0.00	0.00	0.00	LAND	P												
Juvenile	YOY_AP019498	0.00	1.00	0.00	1.00	0.00	0.00	0.00	0.00	LAND	P												
Juvenile	YOY_AP019499	0.00	1.00	0.00	1.00	0.00	0.00	0.00	0.00	LAND	P												
Juvenile	YOY_AP019500	0.00	1.00	0.00	1.00	0.00	0.00	0.00	0.00	LAND	P												
Juvenile	YOY_AP019501	0.00	1.00	0.00	1.00	0.00	0.00	0.00	0.00	LAND	P												
Juvenile	YOY_AP019502	0.00	1.00	0.00	1.00	0.00	0.00	0.00	0.00	LAND	P												
Juvenile	YOY_AP019503	0.00	1.00	0.00	1.00	0.00	0.00	0.00	0.00	LAND	P												
Juvenile	YOY_AP019504	0.00	1.00	0.00	1.00	0.00	0.00	0.00	0.00	LAND	P												
Juvenile	YOY_AP019505	0.00	1.00	0.00	1.00	0.00	0.00	0.00	0.00	LAND	P												
Juvenile	YOY_AP019506	0.00	1.00	0.00	1.00	0.00	0.00	0.00	0.00	LAND	P												
Juvenile	YOY_AP019507	0.00	1.00	0.00	1.00	0.00	0.00	0.00	0.00	LAND	P												
Juvenile	YOY_AP019508	0.00	1.00	0.00	1.00	0.00	0.00	0.00	0.00	LAND	P												
Juvenile	YOY_AP019509	1.00	0.00	1.00	0.00	0.00	0.00	0.00	0.00	ANAD	P	BvAP018689	M	4/12/17	252	4.29E+01	1	BvAP017834	F	4/12/17	315	5.38E+01	1
Juvenile	YOY_AP019510	0.00	1.00	0.00	1.00	0.00	0.00	0.00	0.00	LAND	P												
Juvenile	YOY_AP019511	0.00	1.00	0.00	1.00	0.00	0.00	0.00	0.00	LAND	P												
Juvenile	YOY_AP019512	0.00	1.00	0.00	1.00	0.00	0.00	0.00	0.00	LAND	P												
Juvenile	YOY_AP019513	0.00	1.00	0.00	1.00	0.00	0.00	0.00	0.00	LAND	P												

Juvenile	YOY_AP019514	0.00	1.00	0.00	1.00	0.00	0.00	0.00	0.00	LAND	P												
Juvenile	YOY_AP019515	0.00	1.00	0.00	1.00	0.00	0.00	0.00	0.00	LAND	P												
Juvenile	YOY_AP019516	0.00	1.00	0.00	1.00	0.00	0.00	0.00	0.00	LAND	P												
Juvenile	YOY_AP019517	0.00	1.00	0.00	1.00	0.00	0.00	0.00	0.00	LAND	P												
Juvenile	YOY_AP019518	0.00	1.00	0.00	1.00	0.00	0.00	0.00	0.00	LAND	P												
Juvenile	YOY_AP020446	0.55	0.45	0.00	0.00	0.28	0.72	0.00	0.00	F1	P	BvAP016916	F	3/30/17	277	5.36E+01	1						
Juvenile	YOY_AP019523	0.00	1.00	0.00	1.00	0.00	0.00	0.00	0.00	LAND	P												
Juvenile	YOY_AP019524	0.00	1.00	0.00	1.00	0.00	0.00	0.00	0.00	LAND	P												
Juvenile	YOY_AP019525	1.00	0.00	1.00	0.00	0.00	0.00	0.00	0.00	ANAD	P	BvAP019004	F	4/12/17	274	4.18E+01	1	BvAP017996	M	4/12/17	252	5.00E+01	1
Juvenile	YOY_AP019526	0.20	0.80	0.00	0.00	0.00	0.00	0.00	1.00	LAND_BC	P												
Juvenile	YOY_AP019527	1.00	0.00	1.00	0.00	0.00	0.00	0.00	0.00	ANAD	P	BvAP017369	F	3/30/17	294	5.27E+01	1	BvAP016955	M	3/30/17	277	5.02E+01	1
Juvenile	YOY_AP019528	0.00	1.00	0.00	1.00	0.00	0.00	0.00	0.00	LAND	P												
Juvenile	YOY_AP019529	0.00	1.00	0.00	1.00	0.00	0.00	0.00	0.00	LAND	P												
Juvenile	YOY_AP020494	0.49	0.52	0.00	0.00	0.38	0.59	0.00	0.03	F1	P	BvAP016715	M	3/30/17	252	2.83E+01	1						
Juvenile	YOY_AP019531	0.00	1.00	0.00	1.00	0.00	0.00	0.00	0.00	LAND	P												
Juvenile	YOY_AP019532	0.00	1.00	0.00	1.00	0.00	0.00	0.00	0.00	LAND	P												
Juvenile	YOY_AP019533	0.00	1.00	0.00	1.00	0.00	0.00	0.00	0.00	LAND	P												
Juvenile	YOY_AP019534	0.00	1.00	0.00	1.00	0.00	0.00	0.00	0.00	LAND	P												
Juvenile	YOY_AP019535	0.00	1.00	0.00	1.00	0.00	0.00	0.00	0.00	LAND	P												
Juvenile	YOY_AP019536	0.00	1.00	0.00	1.00	0.00	0.00	0.00	0.00	LAND	P												
Juvenile	YOY_AP019537	0.00	1.00	0.00	1.00	0.00	0.00	0.00	0.00	LAND	P												
Juvenile	YOY_AP019538	0.00	1.00	0.00	1.00	0.00	0.00	0.00	0.00	LAND	P												
Juvenile	YOY_AP019539	0.00	1.00	0.00	1.00	0.00	0.00	0.00	0.00	LAND	P												
Juvenile	YOY_AP019540	0.00	1.00	0.00	1.00	0.00	0.00	0.00	0.00	LAND	P												
Juvenile	YOY_AP019541	0.00	1.00	0.00	1.00	0.00	0.00	0.00	0.00	LAND	P												
Juvenile	YOY_AP019542	0.00	1.00	0.00	1.00	0.00	0.00	0.00	0.00	LAND	P												
Juvenile	YOY_AP019543	0.00	1.00	0.00	1.00	0.00	0.00	0.00	0.00	LAND	P												
Juvenile	YOY_AP019544	1.00	0.00	1.00	0.00	0.00	0.00	0.00	0.00	ANAD	P	BvAP018606	M	4/12/17	256	4.59E+01	1	BvAP018270	F	4/19/17	280	3.79E+01	1
Juvenile	YOY_AP019545	0.00	1.00	0.00	1.00	0.00	0.00	0.00	0.00	LAND	P												
Juvenile	YOY_AP020508	0.59	0.41	0.00	0.00	1.00	0.00	0.00	0.00	F1	P	BvAP016916	F	3/30/17	277	4.63E+01	1						
Juvenile	YOY_AP019547	0.00	1.00	0.00	1.00	0.00	0.00	0.00	0.00	LAND	P												
Juvenile	YOY_AP019548	0.00	1.00	0.00	1.00	0.00	0.00	0.00	0.00	LAND	P												
Juvenile	YOY_AP019549	0.00	1.00	0.00	1.00	0.00	0.00	0.00	0.00	LAND	P												
Juvenile	YOY_AP019550	0.00	1.00	0.00	1.00	0.00	0.00	0.00	0.00	LAND	P												
Juvenile	YOY_AP019551	0.00	1.00	0.00	1.00	0.00	0.00	0.00	0.00	LAND	P												
Juvenile	YOY_AP019552	0.00	1.00	0.00	1.00	0.00	0.00	0.00	0.00	LAND	P												
Juvenile	YOY_AP019553	0.00	1.00	0.00	1.00	0.00	0.00	0.00	0.00	LAND	P												
Juvenile	YOY_AP019554	0.29	0.71	0.00	0.00	0.00	0.01	0.00	0.99	LAND_BC	P												
Juvenile	YOY_AP019555	0.00	1.00	0.00	1.00	0.00	0.00	0.00	0.00	LAND	P												
Juvenile	YOY_AP019556	0.00	1.00	0.00	1.00	0.00	0.00	0.00	0.00	LAND	P												
Juvenile	YOY_AP019557	0.00	1.00	0.00	1.00	0.00	0.00	0.00	0.00	LAND	P												
Juvenile	YOY_AP019558	0.00	1.00	0.00	1.00	0.00	0.00	0.00	0.00	LAND	P												
Juvenile	YOY_AP019559	0.00	1.00	0.00	1.00	0.00	0.00	0.00	0.00	LAND	P												
Juvenile	YOY_AP019560	0.00	1.00	0.00	1.00	0.00	0.00	0.00	0.00	LAND	P												
Juvenile	YOY_AP019561	0.00	1.00	0.00	1.00	0.00	0.00	0.00	0.00	LAND	P												
Juvenile	YOY_AP019562	0.00	1.00	0.00	1.00	0.00	0.00	0.00	0.00	LAND	P												
Juvenile	YOY_AP019563	0.00	1.00	0.00	1.00	0.00	0.00	0.00	0.00	LAND	P												
Juvenile	YOY_AP019564	0.00	1.00	0.00	1.00	0.00	0.00	0.00	0.00	LAND	P												
Juvenile	YOY_AP019565	1.00	0.00	1.00	0.00	0.00	0.00	0.00	0.00	ANAD	P	BvAP019303	?	4/12/17	269	3.56E+01	1	BvAP019021	?	4/12/17	268	4.52E+01	1
Juvenile	YOY_AP019566	0.00	1.00	0.00	1.00	0.00	0.00	0.00	0.00	LAND	P												
Juvenile	YOY_AP019567	1.00	0.00	1.00	0.00	0.00	0.00	0.00	0.00	ANAD	P	BvAP019010	F	4/12/17	253	3.63E+01	1	BvAP018689	M	4/12/17	252	3.48E+01	1
Juvenile	YOY_AP019568	0.00	1.00	0.00	1.00	0.00	0.00	0.00	0.00	LAND	P												
Juvenile	YOY_AP019569	0.00	1.00	0.00	1.00	0.00	0.00	0.00	0.00	LAND	P												
Juvenile	YOY_AP019570	0.00	1.00	0.00	1.00	0.00	0.00	0.00	0.00	LAND	P												
Juvenile	YOY_AP019571	0.00	1.00	0.00	1.00	0.00	0.00	0.00	0.00	LAND	P												
Juvenile	YOY_AP019572	0.00	1.00	0.00	1.00	0.00	0.00	0.00	0.00	LAND	P												
Juvenile	YOY_AP019573	0.00	1.00	0.00	1.00	0.00	0.00	0.00	0.00	LAND	P												
Juvenile	YOY_AP019574	0.00	1.00	0.00	1.00	0.00	0.00	0.00	0.00	LAND	P												
Juvenile	YOY_AP019575	0.24	0.76	0.00	0.00	0.00	0.43	0.00	0.57	LAND_BC	P												
Juvenile	YOY_AP019577	0.00	1.00	0.00	1.00	0.00	0.00	0.00	0.00	LAND	P												
Juvenile	YOY_AP019578	0.00	1.00	0.00	1.00	0.00	0.00	0.00	0.00	LAND	P												
Juvenile	YOY_AP019579	0.00	1.00	0.00	1.00	0.00	0.00	0.00	0.00	LAND	P												
Juvenile	YOY_AP019580	0.23	0.77	0.00	0.00	0.00	0.00	0.00	1.00	LAND_BC	P												
Juvenile	YOY_AP019581	0.00	1.00	0.00	1.00	0.00	0.00	0.00	0.00	LAND	P												
Juvenile	YOY_AP019582	0.00	1.00	0.00	1.00	0.00	0.00	0.00	0.00	LAND	P												
Juvenile	YOY_AP019583	0.00	1.00	0.00	1.00	0.00	0.00	0.00	0.00	LAND	P												
Juvenile	YOY_AP019584	0.00	1.00	0.00	1.00	0.00	0.00	0.00	0.00	LAND	P												
Juvenile	YOY_AP019585	0.00	1.00	0.00	1.00	0.00	0.00	0.00	0.00	LAND	P												
Juvenile	YOY_AP019586	0.00	1.00	0.00	1.00	0.00	0.00	0.00	0.00	LAND	P												
Juvenile	YOY_AP019587	0.00	1.00	0.00	1.00	0.00	0.00	0.00	0.00	LAND	P												
Juvenile	YOY_AP019588	0.00	1.00	0.00	1.00	0.00	0.00	0.00	0.00	LAND	P												
Juvenile	YOY_AP019589	0.00	1.00	0.00	1.00	0.00	0.00	0.00	0.00	LAND	P												
Juvenile	YOY_AP019590	0.00	1.00	0.00	1.00	0.00	0.00	0.00	0.00	LAND	P												
Juvenile	YOY_AP019591	0.00	1.00	0.00	1.00	0.00	0.00	0.00	0.00	LAND	P												
Juvenile	YOY_AP019592	0.00	1.00	0.00	1.00	0.00	0.00	0.00	0.00	LAND	P												
Juvenile	YOY_AP019593	0.00	1.00	0.00	1.00	0.00	0.00	0.00	0.00	LAND	P												
Juvenile	YOY_AP019594	0.00	1.00	0.00	1.00	0.00	0.00	0.00	0.00	LAND	P												
Juvenile	YOY_AP019595	0.00	1.00	0.00	1.00	0.00	0.00	0.00	0.00	LAND	P												
Juvenile	YOY_AP019596	0.00	1.00	0.00	1.00	0.00	0.00	0.00	0.00	LAND	P												
Juvenile	YOY_AP019597	1.00	0.00	1.00	0.00	0.00	0.00	0.00	0.00	ANAD	P	BvAP017589	F	3/30/17	277	3.57E+01	1	BvAP017121	M	3/30/17	259	4.87E+01	1
Juvenile	YOY_AP019598	0.00	1.00	0.00	1.00	0.00	0.00	0.00	0.00	LAND	P												
Juvenile	YOY_AP019599	0.00	1.00	0.00	1.00	0.00	0.00	0.00	0.00	LAND	P												
Juvenile	YOY_AP019600	0.00	1.00	0.00	1.00	0.00	0.00	0.00	0.00	LAND	P												
Juvenile	YOY_AP019601	0.00	1.00	0.00	1.00	0.00	0.00	0.00	0.00	LAND	P												
Juvenile	YOY_AP019602	0.00	1.00	0.00	1.00	0.00	0.00	0.00	0.00	LAND	P												
Juvenile	YOY_AP019603	0.00	1.00	0.00	1.00	0.00	0.00	0.00	0.00	LAND	P												
Juvenile	YOY_AP019604	0.00	1.00	0.00	1.00	0.00	0.00	0.00	0.00	LAND	P												
Juvenile	YOY_AP019675	0.00	1.00	0.00	1.00	0.00	0.00	0.00	0.00	LAND	P												
Juvenile	YOY_AP019676	0.00	1.00	0.00	1.00	0.00	0.00	0.00	0.00	LAND	P												
Juvenile	YOY_AP019677	0.00	1.00	0.00	1.00	0.00	0.00	0.00	0.00	LAND	P												
Juvenile	YOY_AP019678	0.00	1.00	0.00	1.00	0.00	0.00	0.00	0.00	LAND	P												
Juvenile	YOY_AP019679	1.00	0.00	1.00	0.00	0.00	0.00	0.00	0.00	ANAD	P	BvAP017401	F	3/30/17	261	4.02E+01	1	BvAP017008	M	3/30/17	276	3.65E+01	1
Juvenile	YOY_AP019680	0.52	0.48	0.00	0.00	0.52	0.48	0.00	0.00	F1	P	BvAP018655	F	4/12/17	264	3.15E+01	1						
Juvenile	YOY_AP019681	0.00	1.00	0.00	1.00	0.00	0.00	0.00	0.00	LAND	P												
Juvenile	YOY_AP019682	0.00	1.00	0.00	1.00	0.00	0.00	0.00	0.00	LAND	P												
Juvenile	YOY_AP019683	0.00	1.00	0.00	1.00	0.00	0.00	0.00	0.00	LAND	P												
Juvenile	YOY_AP019684	0.00	1.00	0.00	1.00	0.00	0.00	0.00	0.00	LAND	P												
Juvenile	YOY_AP019685	0.00	1.00	0.00	1.00	0.00	0.00	0.00	0.00	LAND	P												
Juvenile	YOY_AP019686	0.00	1.00	0.00	1.00	0.00	0.00	0.00	0.00	LAND	P												
Juvenile	YOY_AP019687	0.00	1.00	0.00	1.00	0.00	0.00	0.00	0.00	LAND	P												
Juvenile	YOY_AP019688	0.00	1.00	0.00	1.00	0.00	0.00	0.00	0.00	LAND	P												

Juvenile	YOY_AP019689	0.00	1.00	0.00	1.00	0.00	0.00	0.00	0.00	LAND	P												
Juvenile	YOY_AP019690	0.00	1.00	0.00	1.00	0.00	0.00	0.00	0.00	LAND	P												
Juvenile	YOY_AP019691	0.00	1.00	0.00	1.00	0.00	0.00	0.00	0.00	LAND	P												
Juvenile	YOY_AP019692	0.27	0.73	0.00	0.00	0.00	0.01	0.00	0.99	LAND_BC	P												
Juvenile	YOY_AP019693	0.00	1.00	0.00	1.00	0.00	0.00	0.00	0.00	LAND	P												
Juvenile	YOY_AP019694	0.00	1.00	0.00	1.00	0.00	0.00	0.00	0.00	LAND	P												
Juvenile	YOY_AP019695	0.00	1.00	0.00	1.00	0.00	0.00	0.00	0.00	LAND	P												
Juvenile	YOY_AP019696	0.00	1.00	0.00	1.00	0.00	0.00	0.00	0.00	LAND	P												
Juvenile	YOY_AP019697	0.00	1.00	0.00	1.00	0.00	0.00	0.00	0.00	LAND	P												
Juvenile	YOY_AP019698	0.00	1.00	0.00	1.00	0.00	0.00	0.00	0.00	LAND	P												
Juvenile	YOY_AP019699	0.00	1.00	0.00	1.00	0.00	0.00	0.00	0.00	LAND	P												
Juvenile	YOY_AP019700	0.00	1.00	0.00	1.00	0.00	0.00	0.00	0.00	LAND	P												
Juvenile	YOY_AP019701	0.00	1.00	0.00	1.00	0.00	0.00	0.00	0.00	LAND	P												
Juvenile	YOY_AP019702	0.00	1.00	0.00	1.00	0.00	0.00	0.00	0.00	LAND	P												
Juvenile	YOY_AP019703	0.00	1.00	0.00	1.00	0.00	0.00	0.00	0.00	LAND	P												
Juvenile	YOY_AP019704	0.00	1.00	0.00	1.00	0.00	0.00	0.00	0.00	LAND	P												
Juvenile	YOY_AP019705	0.00	1.00	0.00	1.00	0.00	0.00	0.00	0.00	LAND	P												
Juvenile	YOY_AP019706	0.00	1.00	0.00	1.00	0.00	0.00	0.00	0.00	LAND	P												
Juvenile	YOY_AP019707	0.00	1.00	0.00	1.00	0.00	0.00	0.00	0.00	LAND	P												
Juvenile	YOY_AP019708	0.44	0.56	0.00	0.00	1.00	0.00	0.00	0.00	F1	P	BvAP017729	F	4/12/17	267	3.05E+01	1						
Juvenile	YOY_AP019709	0.00	1.00	0.00	1.00	0.00	0.00	0.00	0.00	LAND	P												
Juvenile	YOY_AP019710	0.00	1.00	0.00	1.00	0.00	0.00	0.00	0.00	LAND	P												
Juvenile	YOY_AP019711	0.00	1.00	0.00	1.00	0.00	0.00	0.00	0.00	LAND	P												
Juvenile	YOY_AP019712	0.00	1.00	0.00	1.00	0.00	0.00	0.00	0.00	LAND	P												
Juvenile	YOY_AP019713	0.00	1.00	0.00	1.00	0.00	0.00	0.00	0.00	LAND	P												
Juvenile	YOY_AP019714	0.46	0.54	0.00	0.00	1.00	0.00	0.00	0.00	F1	P	BvAP018665	M	4/12/17	246	1.79E+01	1						
Juvenile	YOY_AP019715	0.00	1.00	0.00	1.00	0.00	0.00	0.00	0.00	LAND	P												
Juvenile	YOY_AP019716	0.00	1.00	0.00	1.00	0.00	0.00	0.00	0.00	LAND	P												
Juvenile	YOY_AP019717	0.00	1.00	0.00	1.00	0.00	0.00	0.00	0.00	LAND	P												
Juvenile	YOY_AP019718	0.00	1.00	0.00	1.00	0.00	0.00	0.00	0.00	LAND	P												
Juvenile	YOY_AP019719	0.00	1.00	0.00	1.00	0.00	0.00	0.00	0.00	LAND	P												
Juvenile	YOY_AP019720	0.00	1.00	0.00	1.00	0.00	0.00	0.00	0.00	LAND	P												
Juvenile	YOY_AP019721	0.00	1.00	0.00	1.00	0.00	0.00	0.00	0.00	LAND	P												
Juvenile	YOY_AP019722	0.00	1.00	0.00	1.00	0.00	0.00	0.00	0.00	LAND	P												
Juvenile	YOY_AP019723	0.00	1.00	0.00	1.00	0.00	0.00	0.00	0.00	LAND	P												
Juvenile	YOY_AP019724	0.00	1.00	0.00	1.00	0.00	0.00	0.00	0.00	LAND	P												
Juvenile	YOY_AP019725	0.00	1.00	0.00	1.00	0.00	0.00	0.00	0.00	LAND	P												
Juvenile	YOY_AP019726	0.00	1.00	0.00	1.00	0.00	0.00	0.00	0.00	LAND	P												
Juvenile	YOY_AP019727	0.00	1.00	0.00	1.00	0.00	0.00	0.00	0.00	LAND	P												
Juvenile	YOY_AP019728	0.00	1.00	0.00	1.00	0.00	0.00	0.00	0.00	LAND	P												
Juvenile	YOY_AP019729	0.00	1.00	0.00	1.00	0.00	0.00	0.00	0.00	LAND	P												
Juvenile	YOY_AP019730	0.24	0.76	0.00	0.00	0.00	0.00	0.00	1.00	LAND_BC	P												
Juvenile	YOY_AP019731	0.00	1.00	0.00	1.00	0.00	0.00	0.00	0.00	LAND	P												
Juvenile	YOY_AP019732	0.00	1.00	0.00	1.00	0.00	0.00	0.00	0.00	LAND	P												
Juvenile	YOY_AP019733	0.00	1.00	0.00	1.00	0.00	0.00	0.00	0.00	LAND	P												
Juvenile	YOY_AP019734	0.00	1.00	0.00	1.00	0.00	0.00	0.00	0.00	LAND	P												
Juvenile	YOY_AP019735	1.00	0.00	1.00	0.00	0.00	0.00	0.00	0.00	ANAD	P	BvAP019310	M	4/12/17	264	4.49E+01	1	BvAP018684	F	4/12/17	263	3.66E+01	1
Juvenile	YOY_AP019736	0.00	1.00	0.00	1.00	0.00	0.00	0.00	0.00	LAND	P												
Juvenile	YOY_AP019737	0.00	1.00	0.00	1.00	0.00	0.00	0.00	0.00	LAND	P												
Juvenile	YOY_AP019739	0.00	1.00	0.00	1.00	0.00	0.00	0.00	0.00	LAND	P												
Juvenile	YOY_AP019740	0.00	1.00	0.00	1.00	0.00	0.00	0.00	0.00	LAND	P												
Juvenile	YOY_AP019741	0.00	1.00	0.00	1.00	0.00	0.00	0.00	0.00	LAND	P												
Juvenile	YOY_AP019742	0.00	1.00	0.00	1.00	0.00	0.00	0.00	0.00	LAND	P												
Juvenile	YOY_AP019743	0.00	1.00	0.00	1.00	0.00	0.00	0.00	0.00	LAND	P												
Juvenile	YOY_AP019744	0.00	1.00	0.00	1.00	0.00	0.00	0.00	0.00	LAND	P												
Juvenile	YOY_AP019745	0.00	1.00	0.00	1.00	0.00	0.00	0.00	0.00	LAND	P												
Juvenile	YOY_AP019746	0.00	1.00	0.00	1.00	0.00	0.00	0.00	0.00	LAND	P												
Juvenile	YOY_AP019747	0.00	1.00	0.00	1.00	0.00	0.00	0.00	0.00	LAND	P												
Juvenile	YOY_AP019748	0.00	1.00	0.00	1.00	0.00	0.00	0.00	0.00	LAND	P												
Juvenile	YOY_AP019749	0.00	1.00	0.00	1.00	0.00	0.00	0.00	0.00	LAND	P												
Juvenile	YOY_AP019750	0.00	1.00	0.00	1.00	0.00	0.00	0.00	0.00	LAND	P												
Juvenile	YOY_AP019751	0.00	1.00	0.00	1.00	0.00	0.00	0.00	0.00	LAND	P												
Juvenile	YOY_AP019752	0.00	1.00	0.00	1.00	0.00	0.00	0.00	0.00	LAND	P												
Juvenile	YOY_AP019753	0.00	1.00	0.00	1.00	0.00	0.00	0.00	0.00	LAND	P												
Juvenile	YOY_AP019754	0.00	1.00	0.00	1.00	0.00	0.00	0.00	0.00	LAND	P												
Juvenile	YOY_AP019755	0.00	1.00	0.00	1.00	0.00	0.00	0.00	0.00	LAND	P												
Juvenile	YOY_AP019756	0.00	1.00	0.00	1.00	0.00	0.00	0.00	0.00	LAND	P												
Juvenile	YOY_AP019757	1.00	0.00	1.00	0.00	0.00	0.00	0.00	0.00	ANAD	P	BvAP018677	M	4/12/17	269	3.51E+01	1	?	?	?	?	?	?
Juvenile	YOY_AP019758	0.00	1.00	0.00	1.00	0.00	0.00	0.00	0.00	LAND	P												
Juvenile	YOY_AP019759	0.00	1.00	0.00	1.00	0.00	0.00	0.00	0.00	LAND	P												
Juvenile	YOY_AP019760	0.00	1.00	0.00	1.00	0.00	0.00	0.00	0.00	LAND	P												
Juvenile	YOY_AP019965	0.41	0.59	0.00	0.00	0.24	0.75	0.00	0.01	F1	L	BvAP017955	M	4/12/17	254	2.48E+01	1						
Juvenile	YOY_AP019762	0.00	1.00	0.00	1.00	0.00	0.00	0.00	0.00	LAND	P												
Juvenile	YOY_AP019763	0.00	1.00	0.00	1.00	0.00	0.00	0.00	0.00	LAND	P												
Juvenile	YOY_AP019764	0.00	1.00	0.00	1.00	0.00	0.00	0.00	0.00	LAND	P												
Juvenile	YOY_AP019765	0.00	1.00	0.00	1.00	0.00	0.00	0.00	0.00	LAND	P												
Juvenile	YOY_AP019766	0.02	0.98	0.00	1.00	0.00	0.00	0.00	0.00	LAND	P												
Juvenile	YOY_AP019767	0.00	1.00	0.00	1.00	0.00	0.00	0.00	0.00	LAND	P												
Juvenile	YOY_AP019769	0.00	1.00	0.00	1.00	0.00	0.00	0.00	0.00	LAND	P												
Juvenile	YOY_AP019770	0.19	0.81	0.00	0.00	0.00	0.00	0.00	1.00	LAND_BC	P												
Juvenile	YOY_AP019771	0.00	1.00	0.00	1.00	0.00	0.00	0.00	0.00	LAND	P												
Juvenile	YOY_AP019772	0.00	1.00	0.00	1.00	0.00	0.00	0.00	0.00	LAND	P												
Juvenile	YOY_AP019773	0.00	1.00	0.00	1.00	0.00	0.00	0.00	0.00	LAND	P												
Juvenile	YOY_AP019774	0.00	1.00	0.00	1.00	0.00	0.00	0.00	0.00	LAND	P												
Juvenile	YOY_AP019775	0.00	1.00	0.00	1.00	0.00	0.00	0.00	0.00	LAND	P												
Juvenile	YOY_AP019776	0.00	1.00	0.00	1.00	0.00	0.00	0.00	0.00	LAND	P												
Juvenile	YOY_AP019777	0.00	1.00	0.00	1.00	0.00	0.00	0.00	0.00	LAND	P												
Juvenile	YOY_AP019778	0.00	1.00	0.00	1.00	0.00	0.00	0.00	0.00	LAND	P												
Juvenile	YOY_AP019779	0.00	1.00	0.00	1.00	0.00	0.00	0.00	0.00	LAND	P												
Juvenile	YOY_AP019780	0.00	1.00	0.00	1.00	0.00	0.00	0.00	0.00	LAND	P												
Juvenile	YOY_AP019781	0.00	1.00	0.00	1.00	0.00	0.00	0.00	0.00	LAND	P												
Juvenile	YOY_AP019782	0.00	1.00	0.00	1.00	0.00	0.00	0.00	0.00	LAND	P												
Juvenile	YOY_AP019783	0.00	1.00	0.00	1.00	0.00	0.00	0.00	0.00	LAND	P												
Juvenile	YOY_AP019784	0.00	1.00	0.00	1.00	0.00	0.00	0.00	0.00	LAND	P												
Juvenile	YOY_AP019785	0.00	1.00	0.00	1.00	0.00	0.00	0.00	0.00	LAND	P												
Juvenile	YOY_AP019786	0.00	1.00	0.00	1.00	0.00	0.00	0.00	0.00	LAND	P												
Juvenile	YOY_AP019787	0.00	1.00	0.00	1.00	0.00	0.00	0.00	0.00	LAND	P												
Juvenile	YOY_AP019788	0.00	1.00	0.00	1.00	0.00	0.00	0.00	0.00	LAND	P												
Juvenile	YOY_AP019789	0.00	1.00	0.00	1.00	0.00	0.00	0.00	0.00	LAND	P												
Juvenile	YOY_AP019790	0.00	1.00	0.00	1.00	0.00	0.00	0.00	0.00	LAND	P												
Juvenile	YOY_AP019791	0.00	1.00	0.00	1.00	0.00	0.00	0.00	0.00	LAND	P												

Juvenile	YOY_AP019792	0.00	1.00	0.00	1.00	0.00	0.00	0.00	0.00	LAND	P												
Juvenile	YOY_AP019793	0.00	1.00	0.00	1.00	0.00	0.00	0.00	0.00	LAND	P												
Juvenile	YOY_AP019794	0.00	1.00	0.00	1.00	0.00	0.00	0.00	0.00	LAND	P												
Juvenile	YOY_AP019795	0.00	1.00	0.00	1.00	0.00	0.00	0.00	0.00	LAND	P												
Juvenile	YOY_AP019796	0.00	1.00	0.00	1.00	0.00	0.00	0.00	0.00	LAND	P												
Juvenile	YOY_AP019797	0.00	1.00	0.00	1.00	0.00	0.00	0.00	0.00	LAND	P												
Juvenile	YOY_AP019798	0.00	1.00	0.00	1.00	0.00	0.00	0.00	0.00	LAND	P												
Juvenile	YOY_AP019799	0.00	1.00	0.00	1.00	0.00	0.00	0.00	0.00	LAND	P												
Juvenile	YOY_AP019800	0.00	1.00	0.00	1.00	0.00	0.00	0.00	0.00	LAND	P												
Juvenile	YOY_AP019801	0.00	1.00	0.00	1.00	0.00	0.00	0.00	0.00	LAND	P												
Juvenile	YOY_AP019802	0.00	1.00	0.00	1.00	0.00	0.00	0.00	0.00	LAND	P												
Juvenile	YOY_AP019804	0.00	1.00	0.00	1.00	0.00	0.00	0.00	0.00	LAND	P												
Juvenile	YOY_AP019805	0.00	1.00	0.00	1.00	0.00	0.00	0.00	0.00	LAND	P												
Juvenile	YOY_AP019806	0.21	0.80	0.00	0.00	0.00	0.00	0.00	1.00	LAND_BC	P												
Juvenile	YOY_AP019807	0.00	1.00	0.00	1.00	0.00	0.00	0.00	0.00	LAND	P												
Juvenile	YOY_AP019808	0.00	1.00	0.00	1.00	0.00	0.00	0.00	0.00	LAND	P												
Juvenile	YOY_AP019809	0.00	1.00	0.00	1.00	0.00	0.00	0.00	0.00	LAND	P												
Juvenile	YOY_AP019810	0.00	1.00	0.00	1.00	0.00	0.00	0.00	0.00	LAND	P												
Juvenile	YOY_AP019812	0.00	1.00	0.00	1.00	0.00	0.00	0.00	0.00	LAND	P												
Juvenile	YOY_AP019813	0.00	1.00	0.00	1.00	0.00	0.00	0.00	0.00	LAND	P												
Juvenile	YOY_AP019814	0.00	1.00	0.00	1.00	0.00	0.00	0.00	0.00	LAND	P												
Juvenile	YOY_AP019815	0.00	1.00	0.00	1.00	0.00	0.00	0.00	0.00	LAND	P												
Juvenile	YOY_AP019816	0.00	1.00	0.00	1.00	0.00	0.00	0.00	0.00	LAND	P												
Juvenile	YOY_AP019817	0.00	1.00	0.00	1.00	0.00	0.00	0.00	0.00	LAND	P												
Juvenile	YOY_AP019818	0.00	1.00	0.00	1.00	0.00	0.00	0.00	0.00	LAND	P												
Juvenile	YOY_AP019820	0.00	1.00	0.00	1.00	0.00	0.00	0.00	0.00	LAND	P												
Juvenile	YOY_AP019821	0.00	1.00	0.00	1.00	0.00	0.00	0.00	0.00	LAND	P												
Juvenile	YOY_AP019822	0.00	1.00	0.00	1.00	0.00	0.00	0.00	0.00	LAND	P												
Juvenile	YOY_AP019823	0.00	1.00	0.00	1.00	0.00	0.00	0.00	0.00	LAND	P												
Juvenile	YOY_AP019824	0.00	1.00	0.00	1.00	0.00	0.00	0.00	0.00	LAND	P												
Juvenile	YOY_AP019825	0.00	1.00	0.00	1.00	0.00	0.00	0.00	0.00	LAND	P												
Juvenile	YOY_AP019826	0.00	1.00	0.00	1.00	0.00	0.00	0.00	0.00	LAND	P												
Juvenile	YOY_AP019828	0.00	1.00	0.00	1.00	0.00	0.00	0.00	0.00	LAND	P												
Juvenile	YOY_AP019829	0.00	1.00	0.00	1.00	0.00	0.00	0.00	0.00	LAND	P												
Juvenile	YOY_AP019830	0.00	1.00	0.00	1.00	0.00	0.00	0.00	0.00	LAND	P												
Juvenile	YOY_AP019831	0.00	1.00	0.00	1.00	0.00	0.00	0.00	0.00	LAND	P												
Juvenile	YOY_AP019832	0.00	1.00	0.00	1.00	0.00	0.00	0.00	0.00	LAND	P												
Juvenile	YOY_AP019833	0.00	1.00	0.00	1.00	0.00	0.00	0.00	0.00	LAND	P												
Juvenile	YOY_AP019834	0.00	1.00	0.00	1.00	0.00	0.00	0.00	0.00	LAND	P												
Juvenile	YOY_AP019836	0.00	1.00	0.00	1.00	0.00	0.00	0.00	0.00	LAND	P												
Juvenile	YOY_AP019837	0.00	1.00	0.00	1.00	0.00	0.00	0.00	0.00	LAND	P												
Juvenile	YOY_AP019838	0.00	1.00	0.00	1.00	0.00	0.00	0.00	0.00	LAND	P												
Juvenile	YOY_AP019839	0.00	1.00	0.00	1.00	0.00	0.00	0.00	0.00	LAND	P												
Juvenile	YOY_AP019840	0.00	1.00	0.00	1.00	0.00	0.00	0.00	0.00	LAND	P												
Juvenile	YOY_AP019841	0.00	1.00	0.00	1.00	0.00	0.00	0.00	0.00	LAND	P												
Juvenile	YOY_AP019842	0.00	1.00	0.00	1.00	0.00	0.00	0.00	0.00	LAND	P												
Juvenile	YOY_AP019844	0.00	1.00	0.00	1.00	0.00	0.00	0.00	0.00	LAND	P												
Juvenile	YOY_AP019845	0.00	1.00	0.00	1.00	0.00	0.00	0.00	0.00	LAND	P												
Juvenile	YOY_AP019846	0.00	1.00	0.00	1.00	0.00	0.00	0.00	0.00	LAND	P												
Juvenile	YOY_AP019847	0.00	1.00	0.00	1.00	0.00	0.00	0.00	0.00	LAND	P												
Juvenile	YOY_AP019848	0.00	1.00	0.00	1.00	0.00	0.00	0.00	0.00	LAND	P												
Juvenile	YOY_AP019849	0.00	1.00	0.00	1.00	0.00	0.00	0.00	0.00	LAND	P												
Juvenile	YOY_AP019850	0.00	1.00	0.00	1.00	0.00	0.00	0.00	0.00	LAND	P												
Juvenile	YOY_AP019852	0.00	1.00	0.00	1.00	0.00	0.00	0.00	0.00	LAND	P												
Juvenile	YOY_AP019853	0.00	1.00	0.00	1.00	0.00	0.00	0.00	0.00	LAND	P												
Juvenile	YOY_AP019854	0.00	1.00	0.00	1.00	0.00	0.00	0.00	0.00	LAND	P												
Juvenile	YOY_AP019855	0.00	1.00	0.00	1.00	0.00	0.00	0.00	0.00	LAND	P												
Juvenile	YOY_AP019856	0.00	1.00	0.00	1.00	0.00	0.00	0.00	0.00	LAND	P												
Juvenile	YOY_AP019857	0.00	1.00	0.00	1.00	0.00	0.00	0.00	0.00	LAND	P												
Juvenile	YOY_AP019858	1.00	0.00	1.00	0.00	0.00	0.00	0.00	0.00	ANAD	P	BvAP019340	F	4/19/17	322	3.46E+01	1	BvAP017278	M	3/30/17	264	5.19E+01	1
Juvenile	YOY_AP019860	0.00	1.00	0.00	1.00	0.00	0.00	0.00	0.00	LAND	P												
Juvenile	YOY_AP019861	0.00	1.00	0.00	1.00	0.00	0.00	0.00	0.00	LAND	P												
Juvenile	YOY_AP019862	0.00	1.00	0.00	1.00	0.00	0.00	0.00	0.00	LAND	P												
Juvenile	YOY_AP019863	0.00	1.00	0.00	1.00	0.00	0.00	0.00	0.00	LAND	P												
Juvenile	YOY_AP019865	0.00	1.00	0.00	1.00	0.00	0.00	0.00	0.00	LAND	P												
Juvenile	YOY_AP019866	0.00	1.00	0.00	1.00	0.00	0.00	0.00	0.00	LAND	P												
Juvenile	YOY_AP019868	0.00	1.00	0.00	1.00	0.00	0.00	0.00	0.00	LAND	P												
Juvenile	YOY_AP019869	0.00	1.00	0.00	1.00	0.00	0.00	0.00	0.00	LAND	P												
Juvenile	YOY_AP019870	0.00	1.00	0.00	1.00	0.00	0.00	0.00	0.00	LAND	P												
Juvenile	YOY_AP019871	0.00	1.00	0.00	1.00	0.00	0.00	0.00	0.00	LAND	P												
Juvenile	YOY_AP019872	0.00	1.00	0.00	1.00	0.00	0.00	0.00	0.00	LAND	P												
Juvenile	YOY_AP019873	0.00	1.00	0.00	1.00	0.00	0.00	0.00	0.00	LAND	P												
Juvenile	YOY_AP019874	0.00	1.00	0.00	1.00	0.00	0.00	0.00	0.00	LAND	P												
Juvenile	YOY_AP019876	0.00	1.00	0.00	1.00	0.00	0.00	0.00	0.00	LAND	P												
Juvenile	YOY_AP019877	0.00	1.00	0.00	1.00	0.00	0.00	0.00	0.00	LAND	P												
Juvenile	YOY_AP019878	0.00	1.00	0.00	1.00	0.00	0.00	0.00	0.00	LAND	P												
Juvenile	YOY_AP019879	0.00	1.00	0.00	1.00	0.00	0.00	0.00	0.00	LAND	P												
Juvenile	YOY_AP019880	0.00	1.00	0.00	1.00	0.00	0.00	0.00	0.00	LAND	P												
Juvenile	YOY_AP019881	0.00	1.00	0.00	1.00	0.00	0.00	0.00	0.00	LAND	P												
Juvenile	YOY_AP019882	0.00	1.00	0.00	1.00	0.00	0.00	0.00	0.00	LAND	P												
Juvenile	YOY_AP019883	0.00	1.00	0.00	1.00	0.00	0.00	0.00	0.00	LAND	P												
Juvenile	YOY_AP019884	0.00	1.00	0.00	1.00	0.00	0.00	0.00	0.00	LAND	P												
Juvenile	YOY_AP019885	0.00	1.00	0.00	1.00	0.00	0.00	0.00	0.00	LAND	P												
Juvenile	YOY_AP019886	0.00	1.00	0.00	1.00	0.00	0.00	0.00	0.00	LAND	P												
Juvenile	YOY_AP019887	0.00	1.00	0.00	1.00	0.00	0.00	0.00	0.00	LAND	P												
Juvenile	YOY_AP019888	0.31	0.69	0.00	0.00	0.00	0.01	0.00	0.99	LAND_BC	P												
Juvenile	YOY_AP019890	0.00	1.00	0.00	1.00	0.00	0.00	0.00	0.00	LAND	P												
Juvenile	YOY_AP019892	0.00	1.00	0.00	1.00	0.00	0.00	0.00	0.00	LAND	P												
Juvenile	YOY_AP019893	0.00	1.00	0.00	1.00	0.00	0.00	0.00	0.00	LAND	P												
Juvenile	YOY_AP019894	0.00	1.00	0.00	1.00	0.00	0.00	0.00	0.00	LAND	P												
Juvenile	YOY_AP019895	0.00	1.00	0.00	1.00	0.00	0.00	0.00	0.00	LAND	P												
Juvenile	YOY_AP019896	1.00	0.00	1.00	0.00	0.00	0.00	0.00	0.00	ANAD	P	BvAP019310	M	4/12/17	264	4.83E+01	1	BvAP018601	F	4/12/17	263	3.23E+01	1
Juvenile	YOY_AP019897	0.00	1.00	0.00	1.00	0.00	0.00	0.00	0.00	LAND	P												
Juvenile	YOY_AP019898	0.00	1.00	0.00	1.00	0.00	0.00	0.00	0.00	LAND	P												
Juvenile	YOY_AP019899	0.00	1.00	0.00	1.00	0.00	0.00	0.00	0.00	LAND	P												
Juvenile	YOY_AP019900	0.00	1.00	0.00	1.00	0.00	0.00	0.00	0.00	LAND	P												
Juvenile	YOY_AP019901	0.00	1.00	0.00	1.00	0.00	0.00	0.00	0.00	LAND	P												
Juvenile	YOY_AP019902	0.00	1.00	0.00	1.00	0.00	0.00	0.00	0.00	LAND	P												
Juvenile	YOY_AP019903	0.00	1.00	0.00	1.00	0.00	0.00	0.00	0.00	LAND	P												
Juvenile	YOY_AP019904	0.00	1.00	0.00	1.00	0.00	0.00	0.00	0.00	LAND	P												
Juvenile	YOY_AP019905	0.00	1.00	0.00	1.00	0.00	0.00	0.00	0.00	LAND	P												

Juvenile	YOY_AP019906	0.00	1.00	0.00	1.00	0.00	0.00	0.00	0.00	LAND	P												
Juvenile	YOY_AP019907	0.00	1.00	0.00	1.00	0.00	0.00	0.00	0.00	LAND	P												
Juvenile	YOY_AP019908	0.00	1.00	0.00	1.00	0.00	0.00	0.00	0.00	LAND	P												
Juvenile	YOY_AP019909	0.00	1.00	0.00	1.00	0.00	0.00	0.00	0.00	LAND	P												
Juvenile	YOY_AP019910	0.00	1.00	0.00	1.00	0.00	0.00	0.00	0.00	LAND	P												
Juvenile	YOY_AP019911	0.00	1.00	0.00	1.00	0.00	0.00	0.00	0.00	LAND	P												
Juvenile	YOY_AP019926	1.00	0.00	1.00	0.00	0.00	0.00	0.00	0.00	ANAD	P	BvAP019029	M	4/12/17	262	4.01E+01	1	BvAP018636	F	4/12/17	281	3.77E+01	1
Juvenile	YOY_AP019913	0.00	1.00	0.00	1.00	0.00	0.00	0.00	0.00	LAND	P												
Juvenile	YOY_AP019914	0.00	1.00	0.00	1.00	0.00	0.00	0.00	0.00	LAND	P												
Juvenile	YOY_AP019915	0.00	1.00	0.00	1.00	0.00	0.00	0.00	0.00	LAND	P												
Juvenile	YOY_AP019916	0.00	1.00	0.00	1.00	0.00	0.00	0.00	0.00	LAND	P												
Juvenile	YOY_AP019917	0.00	1.00	0.00	1.00	0.00	0.00	0.00	0.00	LAND	P												
Juvenile	YOY_AP019918	0.00	1.00	0.00	1.00	0.00	0.00	0.00	0.00	LAND	P												
Juvenile	YOY_AP019919	0.00	1.00	0.00	1.00	0.00	0.00	0.00	0.00	LAND	P												
Juvenile	YOY_AP019920	0.22	0.78	0.00	0.00	0.00	0.00	0.00	1.00	LAND_BC	P												
Juvenile	YOY_AP019921	0.00	1.00	0.00	1.00	0.00	0.00	0.00	0.00	LAND	P												
Juvenile	YOY_AP019922	0.00	1.00	0.00	1.00	0.00	0.00	0.00	0.00	LAND	P												
Juvenile	YOY_AP019923	0.00	1.00	0.00	1.00	0.00	0.00	0.00	0.00	LAND	P												
Juvenile	YOY_AP020010	0.54	0.47	0.00	0.00	1.00	0.00	0.00	0.00	F1	P	BvAP018689	M	4/12/17	252	1.20E+01	0.9833						
Juvenile	YOY_AP019925	0.00	1.00	0.00	1.00	0.00	0.00	0.00	0.00	LAND	P												
Juvenile	YOY_AP019932	1.00	0.00	1.00	0.00	0.00	0.00	0.00	0.00	ANAD	P	BvAP018602	F	4/12/17	274	4.13E+01	1	BvAP018417	M	4/19/17	270	6.01E+01	1
Juvenile	YOY_AP019927	0.00	1.00	0.00	1.00	0.00	0.00	0.00	0.00	LAND	P												
Juvenile	YOY_AP019928	0.00	1.00	0.00	1.00	0.00	0.00	0.00	0.00	LAND	P												
Juvenile	YOY_AP019929	0.00	1.00	0.00	1.00	0.00	0.00	0.00	0.00	LAND	P												
Juvenile	YOY_AP019930	0.00	1.00	0.00	1.00	0.00	0.00	0.00	0.00	LAND	P												
Juvenile	YOY_AP019931	0.00	1.00	0.00	1.00	0.00	0.00	0.00	0.00	LAND	P												
Juvenile	YOY_AP019953	1.00	0.00	1.00	0.00	0.00	0.00	0.00	0.00	ANAD	L	BvAP017952	M	4/12/17	261	5.11E+01	1	BvAP017879	F	4/12/17	266	5.11E+01	1
Juvenile	YOY_AP019933	0.00	1.00	0.00	1.00	0.00	0.00	0.00	0.00	LAND	P												
Juvenile	YOY_AP019934	0.00	1.00	0.00	1.00	0.00	0.00	0.00	0.00	LAND	P												
Juvenile	YOY_AP019935	0.00	1.00	0.00	1.00	0.00	0.00	0.00	0.00	LAND	P												
Juvenile	YOY_AP019936	0.00	1.00	0.00	1.00	0.00	0.00	0.00	0.00	LAND	P												
Juvenile	YOY_AP019937	0.00	1.00	0.00	1.00	0.00	0.00	0.00	0.00	LAND	P												
Juvenile	YOY_AP019938	0.00	1.00	0.00	1.00	0.00	0.00	0.00	0.00	LAND	P												
Juvenile	YOY_AP019939	0.00	1.00	0.00	1.00	0.00	0.00	0.00	0.00	LAND	P												
Juvenile	YOY_AP019940	0.00	1.00	0.00	1.00	0.00	0.00	0.00	0.00	LAND	P												
Juvenile	YOY_AP019941	0.00	1.00	0.00	1.00	0.00	0.00	0.00	0.00	LAND	P												
Juvenile	YOY_AP019942	0.00	1.00	0.00	1.00	0.00	0.00	0.00	0.00	LAND	P												
Juvenile	YOY_AP019943	0.00	1.00	0.00	1.00	0.00	0.00	0.00	0.00	LAND	P												
Juvenile	YOY_AP019944	0.00	1.00	0.00	1.00	0.00	0.00	0.00	0.00	LAND	P												
Juvenile	YOY_AP019945	0.00	1.00	0.00	1.00	0.00	0.00	0.00	0.00	LAND	P												
Juvenile	YOY_AP019946	0.00	1.00	0.00	1.00	0.00	0.00	0.00	0.00	LAND	P												
Juvenile	YOY_AP019947	0.00	1.00	0.00	1.00	0.00	0.00	0.00	0.00	LAND	P												
Juvenile	YOY_AP019948	0.00	1.00	0.00	1.00	0.00	0.00	0.00	0.00	LAND	P												
Juvenile	YOY_AP019949	0.00	1.00	0.00	1.00	0.00	0.00	0.00	0.00	LAND	P												
Juvenile	YOY_AP019950	0.00	1.00	0.00	1.00	0.00	0.00	0.00	0.00	LAND	P												
Juvenile	YOY_AP019951	0.00	1.00	0.00	1.00	0.00	0.00	0.00	0.00	LAND	P												
Juvenile	YOY_AP019952	1.00	0.00	1.00	0.00	0.00	0.00	0.00	0.00	ANAD	L	BvAP018432	F	4/19/17	259	3.38E+01	1	BvAP016924	M	3/30/17	257	3.61E+01	1
Juvenile	YOY_AP019957	1.00	0.00	1.00	0.00	0.00	0.00	0.00	0.00	ANAD	L	BvAP019256	F	4/12/17	282	4.48E+01	1	BvAP018803	M	4/12/17	263	3.48E+01	1
Juvenile	YOY_AP019961	0.99	0.01	1.00	0.00	0.00	0.00	0.00	0.00	ANAD	L	BvAP018825	M	4/12/17	247	3.56E+01	1	BvAP017990	F	4/12/17	259	3.44E+01	1
Juvenile	YOY_AP019955	1.00	0.00	1.00	0.00	0.00	0.00	0.00	0.00	ANAD	L	BvAP017189	M	3/30/17	286	3.54E+01	1	BvAP016961	F	3/30/17	266	5.26E+01	1
Juvenile	YOY_AP019956	0.00	1.00	0.00	1.00	0.00	0.00	0.00	0.00	LAND	L												
Juvenile	YOY_AP019962	1.00	0.00	1.00	0.00	0.00	0.00	0.00	0.00	ANAD	L	BvAP019019	M	4/12/17	266	3.77E+01	1	BvAP018570	F	4/12/17	272	6.94E+01	1
Juvenile	YOY_AP019958	1.00	0.00	1.00	0.00	0.00	0.00	0.00	0.00	ANAD	L	BvAP017950	F	4/12/17	275	3.29E+01	1	BvAP017603	M	3/30/17	265	3.26E+01	1
Juvenile	YOY_AP019959	0.00	1.00	0.00	1.00	0.00	0.00	0.00	0.00	LAND	L												
Juvenile	YOY_AP019960	1.00	0.00	1.00	0.00	0.00	0.00	0.00	0.00	ANAD	L	BvAP019265	M	4/12/17	252	4.84E+01	1	BvAP017096	F	3/30/17	274	6.61E+01	1
Juvenile	YOY_AP019967	1.00	0.00	1.00	0.00	0.00	0.00	0.00	0.00	ANAD	L	BvAP018646	M	4/12/17	254	3.14E+01	1	BvAP018570	F	4/12/17	272	5.60E+01	1
Juvenile	YOY_AP019975	1.00	0.00	1.00	0.00	0.00	0.00	0.00	0.00	ANAD	L	BvAP018803	M	4/12/17	263	4.37E+01	1	BvAP017997	F	4/12/17	269	5.09E+01	1
Juvenile	YOY_AP019963	0.28	0.72	0.00	0.00	0.00	0.00	0.00	1.00	LAND_BC	L												
Juvenile	YOY_AP019964	0.00	1.00	0.00	1.00	0.00	0.00	0.00	0.00	LAND	L												
Juvenile	YOY_AP020181	0.56	0.44	0.00	0.00	0.02	0.97	0.01	0.00	F1	P	BvAP018640	M	4/12/17	248	1.98E+01	1						
Juvenile	YOY_AP019966	0.00	1.00	0.00	1.00	0.00	0.00	0.00	0.00	LAND	L												
Juvenile	YOY_AP020053	1.00	0.00	1.00	0.00	0.00	0.00	0.00	0.00	ANAD	P	BvAP019033	F	4/12/17	288	4.25E+01	1	BvAP017792	M	4/12/17	268	4.39E+01	1
Juvenile	YOY_AP019968	1.00	0.00	1.00	0.00	0.00	0.00	0.00	0.00	ANAD	L	BvAP019325	M	4/12/17	266	3.78E+01	1	BvAP017564	F	3/30/17	283	4.76E+01	1
Juvenile	YOY_AP019969	0.00	1.00	0.00	1.00	0.00	0.00	0.00	0.00	LAND	L												
Juvenile	YOY_AP019971	0.00	1.00	0.00	1.00	0.00	0.00	0.00	0.00	LAND	L												
Juvenile	YOY_AP020192	0.99	0.01	1.00	0.00	0.00	0.00	0.00	0.00	ANAD	P	BvAP018760	F	4/12/17	261	4.87E+01	1	BvAP018392	M	4/19/17	263	3.82E+01	1
Juvenile	YOY_AP019973	0.00	1.00	0.00	1.00	0.00	0.00	0.00	0.00	LAND	L												
Juvenile	YOY_AP019974	1.00	0.00	1.00	0.00	0.00	0.00	0.00	0.00	ANAD	L	BvAP017284	F	3/30/17	279	4.89E+01	1	BvAP016697	M	3/30/17	248	2.38E+01	1
Juvenile	YOY_AP020358	1.00	0.00	1.00	0.00	0.00	0.00	0.00	0.00	ANAD	P	BvAP019017	M	4/12/17	255	3.83E+01	1	BvAP018100	F	4/19/17	263	3.71E+01	1
Juvenile	YOY_AP019976	0.00	1.00	0.00	1.00	0.00	0.00	0.00	0.00	LAND	L												
Juvenile	YOY_AP019977	0.16	0.84	0.00	0.00	0.00	0.00	0.00	1.00	LAND_BC	L												
Juvenile	YOY_AP019978	0.00	1.00	0.00	1.00	0.00	0.00	0.00	0.00	LAND	L												
Juvenile	YOY_AP019979	0.00	1.00	0.00	1.00	0.00	0.00	0.00	0.00	LAND	L												
Juvenile	YOY_AP019980	1.00	0.00	1.00	0.00	0.00	0.00	0.00	0.00	ANAD	L	BvAP018601	F	4/12/17	263	3.54E+01	1	BvAP017707	M	3/30/17	279	6.09E+01	1
Juvenile	YOY_AP020366	1.00	0.00	1.00	0.00	0.00	0.00	0.00	0.00	ANAD	P	BvAP018964	M	4/12/17	256	4.87E+01	1	BvAP017834	F	4/12/17	315	3.30E+01	1
Juvenile	YOY_AP019982	0.00	1.00	0.00	1.00	0.00	0.00	0.00	0.00	LAND	L												
Juvenile	YOY_AP019983	0.00	1.00	0.00	1.00	0.00	0.00	0.00	0.00	LAND	L												
Juvenile	YOY_AP019984	0.00	1.00	0.00	1.00	0.00	0.00	0.00	0.00	LAND	L												
Juvenile	YOY_AP019985	0.00	1.00	0.00	1.00	0.00	0.00	0.00	0.00	LAND	L												
Juvenile	YOY_AP019986	0.00	1.00	0.00	1.00	0.00	0.00	0.00	0.00	LAND	L												
Juvenile	YOY_AP019987	0.00	1.00	0.00	1.00	0.00	0.00	0.00	0.00	LAND	L												
Juvenile	YOY_AP019988	0.00	1.00	0.00	1.00	0.00	0.00	0.00	0.00	LAND	L												
Juvenile	YOY_AP019989	0.00	1.00	0.00	1.00	0.00	0.00	0.00	0.00	LAND	L												
Juvenile	YOY_AP019990	0.00	1.00	0.00	1.00	0.00	0.00	0.00	0.00	LAND	L												
Juvenile	YOY_AP019991	0.00	1.00	0.00	1.00	0.00	0.00	0.00	0.00	LAND	L												
Juvenile	YOY_AP019992	0.00	1.00	0.00	1.00	0.00	0.00	0.00	0.00	LAND	L												
Juvenile	YOY_AP019993	0.00	1.00	0.00	1.00	0.00	0.00	0.00	0.00	LAND	L												
Juvenile	YOY_AP019994	0.00	1.00	0.00	1.00	0.00	0.00	0.00	0.00	LAND	L												
Juvenile	YOY_AP019995	0.00	1.00	0.00	1.00	0.00	0.00	0.00	0.00	LAND	L												
Juvenile	YOY_AP019996	0.00	1.00	0.00	1.00	0.00	0.00	0.00	0.00	LAND	L												
Juvenile	YOY_AP019997	0.00	1.00	0.00	1.00	0.00	0.00	0.00	0.00	LAND	L												
Juvenile	YOY_AP019998	0.00	1.00	0.00	1.00	0.00	0.00	0.00	0.00	LAND	P												
Juvenile	YOY_AP020457	1.00	0.00	1.00	0.00	0.00	0.00	0.00	0.00	ANAD	P	BvAP018606	M	4/12/17	256	5.04E+01	1	BvAP018052	F	4/19/17	286	4.29E+01	1
Juvenile	YOY_AP020000	0.00	1.00	0.00	1.00	0.00	0.00	0.00	0.00	LAND	P												
Juvenile	YOY_AP020001	0.00	1.00	0.00	1.00	0.00	0.00	0.00	0.00	LAND	P												
Juvenile	YOY_AP020002	0.00	1.00	0.00	1.00	0.00	0.00	0.00	0.00	LAND	P												
Juvenile	YOY_AP020003	0.00	1.00	0.00	1.00	0.00	0.00	0.00	0.00	LAND	P												
Juvenile	YOY_AP020004	0.00	1.00	0.00	1.00	0.00	0.00	0.00	0.00	LAND	P												
Juvenile	YOY_AP020005	0.27	0.73	0.00	0.00	0.00	0.00	0.00	1.00	LAND_BC	P												
Juvenile	YOY_AP020006	0.00	1.00	0.00	1.00	0.00	0.00	0.00	0.00	LAND	P												
Juvenile	YOY_AP020008	0.00	1.00	0.00	1.00	0.00	0.00	0.00	0.00	LAND	P												

Juvenile	YOY_AP020009	0.00	1.00	0.00	1.00	0.00	0.00	0.00	0.00	LAND	P												
Juvenile	YOY_AP020353	0.50	0.50	0.00	0.00	0.19	0.81	0.00	0.00	F1	P	BvAP017886	F	4/12/17	275	2.13E+01	1						
Juvenile	YOY_AP020011	0.00	1.00	0.00	1.00	0.00	0.00	0.00	0.00	LAND	P												
Juvenile	YOY_AP020012	0.00	1.00	0.00	1.00	0.00	0.00	0.00	0.00	LAND	P												
Juvenile	YOY_AP020013	0.00	1.00	0.00	1.00	0.00	0.00	0.00	0.00	LAND	P												
Juvenile	YOY_AP020014	0.00	1.00	0.00	1.00	0.00	0.00	0.00	0.00	LAND	P												
Juvenile	YOY_AP020015	0.00	1.00	0.00	1.00	0.00	0.00	0.00	0.00	LAND	P												
Juvenile	YOY_AP020016	0.00	1.00	0.00	1.00	0.00	0.00	0.00	0.00	LAND	P												
Juvenile	YOY_AP020017	0.00	1.00	0.00	1.00	0.00	0.00	0.00	0.00	LAND	P												
Juvenile	YOY_AP020018	0.00	1.00	0.00	1.00	0.00	0.00	0.00	0.00	LAND	P												
Juvenile	YOY_AP020019	0.00	1.00	0.00	1.00	0.00	0.00	0.00	0.00	LAND	P												
Juvenile	YOY_AP020020	0.00	1.00	0.00	1.00	0.00	0.00	0.00	0.00	LAND	P												
Juvenile	YOY_AP020021	0.00	1.00	0.00	1.00	0.00	0.00	0.00	0.00	LAND	P												
Juvenile	YOY_AP020022	0.00	1.00	0.00	1.00	0.00	0.00	0.00	0.00	LAND	P												
Juvenile	YOY_AP020401	0.53	0.47	0.00	0.00	0.84	0.16	0.00	0.00	F1	P	BvAP018620	M	4/12/17	258	3.16E+01	1						
Juvenile	YOY_AP020024	0.00	1.00	0.00	1.00	0.00	0.00	0.00	0.00	LAND	P												
Juvenile	YOY_AP020025	0.00	1.00	0.00	1.00	0.00	0.00	0.00	0.00	LAND	P												
Juvenile	YOY_AP020026	0.00	1.00	0.00	1.00	0.00	0.00	0.00	0.00	LAND	P												
Juvenile	YOY_AP020027	0.00	1.00	0.00	1.00	0.00	0.00	0.00	0.00	LAND	P												
Juvenile	YOY_AP020028	0.00	1.00	0.00	1.00	0.00	0.00	0.00	0.00	LAND	P												
Juvenile	YOY_AP020029	0.00	1.00	0.00	1.00	0.00	0.00	0.00	0.00	LAND	P												
Juvenile	YOY_AP020030	0.00	1.00	0.00	1.00	0.00	0.00	0.00	0.00	LAND	P												
Juvenile	YOY_AP020031	0.00	1.00	0.00	1.00	0.00	0.00	0.00	0.00	LAND	P												
Juvenile	YOY_AP020032	0.00	1.00	0.00	1.00	0.00	0.00	0.00	0.00	LAND	P												
Juvenile	YOY_AP020033	0.00	1.00	0.00	1.00	0.00	0.00	0.00	0.00	LAND	P												
Juvenile	YOY_AP020034	0.00	1.00	0.00	1.00	0.00	0.00	0.00	0.00	LAND	P												
Juvenile	YOY_AP020035	0.00	1.00	0.00	1.00	0.00	0.00	0.00	0.00	LAND	P												
Juvenile	YOY_AP020036	0.00	1.00	0.00	1.00	0.00	0.00	0.00	0.00	LAND	P												
Juvenile	YOY_AP020037	0.00	1.00	0.00	1.00	0.00	0.00	0.00	0.00	LAND	P												
Juvenile	YOY_AP020038	0.00	1.00	0.00	1.00	0.00	0.00	0.00	0.00	LAND	P												
Juvenile	YOY_AP020039	0.00	1.00	0.00	1.00	0.00	0.00	0.00	0.00	LAND	P												
Juvenile	YOY_AP020040	0.00	1.00	0.00	1.00	0.00	0.00	0.00	0.00	LAND	P												
Juvenile	YOY_AP020041	0.20	0.80	0.00	0.00	0.00	0.00	0.00	1.00	LAND_BC	P												
Juvenile	YOY_AP020042	0.00	1.00	0.00	1.00	0.00	0.00	0.00	0.00	LAND	P												
Juvenile	YOY_AP020043	0.00	1.00	0.00	1.00	0.00	0.00	0.00	0.00	LAND	P												
Juvenile	YOY_AP020044	0.00	1.00	0.00	1.00	0.00	0.00	0.00	0.00	LAND	P												
Juvenile	YOY_AP020045	0.00	1.00	0.00	1.00	0.00	0.00	0.00	0.00	LAND	P												
Juvenile	YOY_AP020046	0.00	1.00	0.00	1.00	0.00	0.00	0.00	0.00	LAND	P												
Juvenile	YOY_AP020047	0.00	1.00	0.00	1.00	0.00	0.00	0.00	0.00	LAND	P												
Juvenile	YOY_AP020048	0.00	1.00	0.00	1.00	0.00	0.00	0.00	0.00	LAND	P												
Juvenile	YOY_AP020049	0.00	1.00	0.00	1.00	0.00	0.00	0.00	0.00	LAND	P												
Juvenile	YOY_AP020050	0.00	1.00	0.00	1.00	0.00	0.00	0.00	0.00	LAND	P												
Juvenile	YOY_AP020051	0.00	1.00	0.00	1.00	0.00	0.00	0.00	0.00	LAND	P												
Juvenile	YOY_AP020052	0.00	1.00	0.00	1.00	0.00	0.00	0.00	0.00	LAND	P												
Juvenile	YOY_AP020507	1.00	0.00	1.00	0.00	0.00	0.00	0.00	0.00	ANAD	P	BvAP018816	F	4/12/17	278	3.73E+01	1	BvAP018629	M	4/12/17	245	4.17E+01	1
Juvenile	YOY_AP020054	0.00	1.00	0.00	1.00	0.00	0.00	0.00	0.00	LAND	P												
Juvenile	YOY_AP020055	0.00	1.00	0.00	1.00	0.00	0.00	0.00	0.00	LAND	P												
Juvenile	YOY_AP020056	0.00	1.00	0.00	1.00	0.00	0.00	0.00	0.00	LAND	P												
Juvenile	YOY_AP020057	0.00	1.00	0.00	1.00	0.00	0.00	0.00	0.00	LAND	P												
Juvenile	YOY_AP020058	0.00	1.00	0.00	1.00	0.00	0.00	0.00	0.00	LAND	P												
Juvenile	YOY_AP020059	0.00	1.00	0.00	1.00	0.00	0.00	0.00	0.00	LAND	P												
Juvenile	YOY_AP020060	0.00	1.00	0.00	1.00	0.00	0.00	0.00	0.00	LAND	P												
Juvenile	YOY_AP020061	0.00	1.00	0.00	1.00	0.00	0.00	0.00	0.00	LAND	P												
Juvenile	YOY_AP020062	0.00	1.00	0.00	1.00	0.00	0.00	0.00	0.00	LAND	P												
Juvenile	YOY_AP020063	0.00	1.00	0.00	1.00	0.00	0.00	0.00	0.00	LAND	P												
Juvenile	YOY_AP020064	0.00	1.00	0.00	1.00	0.00	0.00	0.00	0.00	LAND	P												
Juvenile	YOY_AP020065	0.00	1.00	0.00	1.00	0.00	0.00	0.00	0.00	LAND	P												
Juvenile	YOY_AP020067	0.00	1.00	0.00	1.00	0.00	0.00	0.00	0.00	LAND	P												
Juvenile	YOY_AP020068	0.00	1.00	0.00	1.00	0.00	0.00	0.00	0.00	LAND	P												
Juvenile	YOY_AP020069	0.00	1.00	0.00	1.00	0.00	0.00	0.00	0.00	LAND	P												
Juvenile	YOY_AP020070	0.00	1.00	0.00	1.00	0.00	0.00	0.00	0.00	LAND	P												
Juvenile	YOY_AP020071	0.00	1.00	0.00	1.00	0.00	0.00	0.00	0.00	LAND	P												
Juvenile	YOY_AP020072	0.00	1.00	0.00	1.00	0.00	0.00	0.00	0.00	LAND	P												
Juvenile	YOY_AP020073	0.00	1.00	0.00	1.00	0.00	0.00	0.00	0.00	LAND	P												
Juvenile	YOY_AP020074	0.00	1.00	0.00	1.00	0.00	0.00	0.00	0.00	LAND	P												
Juvenile	YOY_AP020075	0.00	1.00	0.00	1.00	0.00	0.00	0.00	0.00	LAND	P												
Juvenile	YOY_AP020076	0.00	1.00	0.00	1.00	0.00	0.00	0.00	0.00	LAND	P												
Juvenile	YOY_AP020077	0.00	1.00	0.00	1.00	0.00	0.00	0.00	0.00	LAND	P												
Juvenile	YOY_AP020078	0.00	1.00	0.00	1.00	0.00	0.00	0.00	0.00	LAND	P												
Juvenile	YOY_AP020079	0.00	1.00	0.00	1.00	0.00	0.00	0.00	0.00	LAND	P												
Juvenile	YOY_AP020080	0.00	1.00	0.00	1.00	0.00	0.00	0.00	0.00	LAND	P												
Juvenile	YOY_AP020081	0.00	1.00	0.00	1.00	0.00	0.00	0.00	0.00	LAND	P												
Juvenile	YOY_AP020082	0.00	1.00	0.00	1.00	0.00	0.00	0.00	0.00	LAND	P												
Juvenile	YOY_AP020083	0.00	1.00	0.00	1.00	0.00	0.00	0.00	0.00	LAND	P												
Juvenile	YOY_AP020084	0.00	1.00	0.00	1.00	0.00	0.00	0.00	0.00	LAND	P												
Juvenile	YOY_AP020085	0.00	1.00	0.00	1.00	0.00	0.00	0.00	0.00	LAND	P												
Juvenile	YOY_AP020086	0.00	1.00	0.00	1.00	0.00	0.00	0.00	0.00	LAND	P												
Juvenile	YOY_AP020087	0.00	1.00	0.00	1.00	0.00	0.00	0.00	0.00	LAND	P												
Juvenile	YOY_AP020088	0.00	1.00	0.00	1.00	0.00	0.00	0.00	0.00	LAND	P												
Juvenile	YOY_AP020089	0.00	1.00	0.00	1.00	0.00	0.00	0.00	0.00	LAND	P												
Juvenile	YOY_AP020090	0.00	1.00	0.00	1.00	0.00	0.00	0.00	0.00	LAND	P												
Juvenile	YOY_AP020091	0.00	1.00	0.00	1.00	0.00	0.00	0.00	0.00	LAND	P												
Juvenile	YOY_AP020092	0.00	1.00	0.00	1.00	0.00	0.00	0.00	0.00	LAND	P												
Juvenile	YOY_AP020093	0.00	1.00	0.00	1.00	0.00	0.00	0.00	0.00	LAND	P												
Juvenile	YOY_AP020094	0.00	1.00	0.00	1.00	0.00	0.00	0.00	0.00	LAND	P												
Juvenile	YOY_AP020095	0.00	1.00	0.00	1.00	0.00	0.00	0.00	0.00	LAND	P												
Juvenile	YOY_AP020096	0.00	1.00	0.00	1.00	0.00	0.00	0.00	0.00	LAND	P												
Juvenile	YOY_AP020097	0.00	1.00	0.00	1.00	0.00	0.00	0.00	0.00	LAND	P												
Juvenile	YOY_AP020098	0.21	0.79	0.00	0.00	0.00	0.00	0.00	1.00	LAND_BC	P												
Juvenile	YOY_AP020099	0.00	1.00	0.00	1.00	0.00	0.00	0.00	0.00	LAND	P												
Juvenile	YOY_AP020100	0.00	1.00	0.00	1.00	0.00	0.00	0.00	0.00	LAND	P												
Juvenile	YOY_AP020101	0.00	1.00	0.00	1.00	0.00	0.00	0.00	0.00	LAND	P												
Juvenile	YOY_AP020102	0.00	1.00	0.00	1.00	0.00	0.00	0.00	0.00	LAND	P												
Juvenile	YOY_AP020103	0.00	1.00	0.00	1.00	0.00	0.00	0.00	0.00	LAND	P												
Juvenile	YOY_AP020104	0.00	1.00	0.00	1.00	0.00	0.00	0.00	0.00	LAND	P												
Juvenile	YOY_AP020105	0.00	1.00	0.00	1.00	0.00	0.00	0.00	0.00	LAND	P												
Juvenile	YOY_AP020106	0.00	1.00	0.00	1.00	0.00	0.00	0.00	0.00	LAND	P												
Juvenile	YOY_AP020107	0.00	1.00	0.00	1.00	0.00	0.00	0.00	0.00	LAND	P												
Juvenile	YOY_AP020108	0.00	1.00	0.00	1.00	0.00	0.00	0.00	0.00	LAND	P												
Juvenile	YOY_AP020109	0.00	1.00	0.00	1.00	0.00	0.00	0.00	0.00	LAND	P												
Juvenile	YOY_AP020110	0.00	1.00	0.00	1.00	0.00	0.00	0.00	0.00	LAND	P												

Juvenile	YOY_AP020111	0.00	1.00	0.00	1.00	0.00	0.00	0.00	0.00	LAND	P												
Juvenile	YOY_AP020112	0.00	1.00	0.00	1.00	0.00	0.00	0.00	0.00	LAND	P												
Juvenile	YOY_AP020113	0.00	1.00	0.00	1.00	0.00	0.00	0.00	0.00	LAND	P												
Juvenile	YOY_AP020114	0.00	1.00	0.00	1.00	0.00	0.00	0.00	0.00	LAND	P												
Juvenile	YOY_AP020115	0.00	1.00	0.00	1.00	0.00	0.00	0.00	0.00	LAND	P												
Juvenile	YOY_AP020116	0.00	1.00	0.00	1.00	0.00	0.00	0.00	0.00	LAND	P												
Juvenile	YOY_AP020117	0.00	1.00	0.00	1.00	0.00	0.00	0.00	0.00	LAND	P												
Juvenile	YOY_AP020118	0.00	1.00	0.00	1.00	0.00	0.00	0.00	0.00	LAND	P												
Juvenile	YOY_AP020119	0.00	1.00	0.00	1.00	0.00	0.00	0.00	0.00	LAND	P												
Juvenile	YOY_AP020120	0.00	1.00	0.00	1.00	0.00	0.00	0.00	0.00	LAND	P												
Juvenile	YOY_AP020121	0.00	1.00	0.00	1.00	0.00	0.00	0.00	0.00	LAND	P												
Juvenile	YOY_AP020122	0.00	1.00	0.00	1.00	0.00	0.00	0.00	0.00	LAND	P												
Juvenile	YOY_AP020123	0.00	1.00	0.00	1.00	0.00	0.00	0.00	0.00	LAND	P												
Juvenile	YOY_AP020124	0.00	1.00	0.00	1.00	0.00	0.00	0.00	0.00	LAND	P												
Juvenile	YOY_AP020125	0.00	1.00	0.00	1.00	0.00	0.00	0.00	0.00	LAND	P												
Juvenile	YOY_AP020126	0.00	1.00	0.00	1.00	0.00	0.00	0.00	0.00	LAND	P												
Juvenile	YOY_AP020127	0.00	1.00	0.00	1.00	0.00	0.00	0.00	0.00	LAND	P												
Juvenile	YOY_AP020128	0.30	0.71	0.00	0.00	0.00	0.00	0.00	1.00	LAND_BC	P												
Juvenile	YOY_AP020129	0.00	1.00	0.00	1.00	0.00	0.00	0.00	0.00	LAND	P												
Juvenile	YOY_AP020130	0.00	1.00	0.00	1.00	0.00	0.00	0.00	0.00	LAND	P												
Juvenile	YOY_AP020131	0.00	1.00	0.00	1.00	0.00	0.00	0.00	0.00	LAND	P												
Juvenile	YOY_AP020132	0.00	1.00	0.00	1.00	0.00	0.00	0.00	0.00	LAND	P												
Juvenile	YOY_AP020133	0.00	1.00	0.00	1.00	0.00	0.00	0.00	0.00	LAND	P												
Juvenile	YOY_AP020134	0.00	1.00	0.00	1.00	0.00	0.00	0.00	0.00	LAND	P												
Juvenile	YOY_AP020135	0.00	1.00	0.00	1.00	0.00	0.00	0.00	0.00	LAND	P												
Juvenile	YOY_AP020136	0.00	1.00	0.00	1.00	0.00	0.00	0.00	0.00	LAND	P												
Juvenile	YOY_AP020137	0.00	1.00	0.00	1.00	0.00	0.00	0.00	0.00	LAND	P												
Juvenile	YOY_AP020138	0.00	1.00	0.00	1.00	0.00	0.00	0.00	0.00	LAND	P												
Juvenile	YOY_AP020139	0.00	1.00	0.00	1.00	0.00	0.00	0.00	0.00	LAND	P												
Juvenile	YOY_AP020140	0.00	1.00	0.00	1.00	0.00	0.00	0.00	0.00	LAND	P												
Juvenile	YOY_AP020141	0.00	1.00	0.00	1.00	0.00	0.00	0.00	0.00	LAND	P												
Juvenile	YOY_AP020142	0.00	1.00	0.00	1.00	0.00	0.00	0.00	0.00	LAND	P												
Juvenile	YOY_AP020143	0.00	1.00	0.00	1.00	0.00	0.00	0.00	0.00	LAND	P												
Juvenile	YOY_AP020144	0.00	1.00	0.00	1.00	0.00	0.00	0.00	0.00	LAND	P												
Juvenile	YOY_AP020145	0.00	1.00	0.00	1.00	0.00	0.00	0.00	0.00	LAND	P												
Juvenile	YOY_AP020146	0.00	1.00	0.00	1.00	0.00	0.00	0.00	0.00	LAND	P												
Juvenile	YOY_AP020147	0.00	1.00	0.00	1.00	0.00	0.00	0.00	0.00	LAND	P												
Juvenile	YOY_AP020148	0.00	1.00	0.00	1.00	0.00	0.00	0.00	0.00	LAND	P												
Juvenile	YOY_AP020149	0.00	1.00	0.00	1.00	0.00	0.00	0.00	0.00	LAND	P												
Juvenile	YOY_AP020150	0.00	1.00	0.00	1.00	0.00	0.00	0.00	0.00	LAND	P												
Juvenile	YOY_AP020151	0.00	1.00	0.00	1.00	0.00	0.00	0.00	0.00	LAND	P												
Juvenile	YOY_AP020153	0.00	1.00	0.00	1.00	0.00	0.00	0.00	0.00	LAND	P												
Juvenile	YOY_AP020154	0.00	1.00	0.00	1.00	0.00	0.00	0.00	0.00	LAND	P												
Juvenile	YOY_AP020155	0.00	1.00	0.00	1.00	0.00	0.00	0.00	0.00	LAND	P												
Juvenile	YOY_AP020156	0.00	1.00	0.00	1.00	0.00	0.00	0.00	0.00	LAND	P												
Juvenile	YOY_AP020157	0.00	1.00	0.00	1.00	0.00	0.00	0.00	0.00	LAND	P												
Juvenile	YOY_AP020158	0.00	1.00	0.00	1.00	0.00	0.00	0.00	0.00	LAND	P												
Juvenile	YOY_AP020159	0.00	1.00	0.00	1.00	0.00	0.00	0.00	0.00	LAND	P												
Juvenile	YOY_AP020160	0.00	1.00	0.00	1.00	0.00	0.00	0.00	0.00	LAND	P												
Juvenile	YOY_AP020161	0.00	1.00	0.00	1.00	0.00	0.00	0.00	0.00	LAND	P												
Juvenile	YOY_AP020162	0.00	1.00	0.00	1.00	0.00	0.00	0.00	0.00	LAND	P												
Juvenile	YOY_AP020163	0.00	1.00	0.00	1.00	0.00	0.00	0.00	0.00	LAND	P												
Juvenile	YOY_AP020164	0.00	1.00	0.00	1.00	0.00	0.00	0.00	0.00	LAND	P												
Juvenile	YOY_AP020165	0.00	1.00	0.00	1.00	0.00	0.00	0.00	0.00	LAND	P												
Juvenile	YOY_AP020166	0.00	1.00	0.00	1.00	0.00	0.00	0.00	0.00	LAND	P												
Juvenile	YOY_AP020167	0.00	1.00	0.00	1.00	0.00	0.00	0.00	0.00	LAND	P												
Juvenile	YOY_AP020168	0.00	1.00	0.00	1.00	0.00	0.00	0.00	0.00	LAND	P												
Juvenile	YOY_AP020169	0.00	1.00	0.00	1.00	0.00	0.00	0.00	0.00	LAND	P												
Juvenile	YOY_AP020170	0.00	1.00	0.00	1.00	0.00	0.00	0.00	0.00	LAND	P												
Juvenile	YOY_AP020171	0.00	1.00	0.00	1.00	0.00	0.00	0.00	0.00	LAND	P												
Juvenile	YOY_AP020172	0.00	1.00	0.00	1.00	0.00	0.00	0.00	0.00	LAND	P												
Juvenile	YOY_AP020173	0.00	1.00	0.00	1.00	0.00	0.00	0.00	0.00	LAND	P												
Juvenile	YOY_AP020174	0.00	1.00	0.00	1.00	0.00	0.00	0.00	0.00	LAND	P												
Juvenile	YOY_AP020175	0.00	1.00	0.00	1.00	0.00	0.00	0.00	0.00	LAND	P												
Juvenile	YOY_AP020176	0.00	1.00	0.00	1.00	0.00	0.00	0.00	0.00	LAND	P												
Juvenile	YOY_AP020177	0.00	1.00	0.00	1.00	0.00	0.00	0.00	0.00	LAND	P												
Juvenile	YOY_AP020178	0.00	1.00	0.00	1.00	0.00	0.00	0.00	0.00	LAND	P												
Juvenile	YOY_AP020179	0.18	0.82	0.00	0.00	0.00	0.00	0.00	1.00	LAND_BC	P												
Juvenile	YOY_AP020180	0.00	1.00	0.00	1.00	0.00	0.00	0.00	0.00	LAND	P												
Juvenile	YOY_AP020429	0.48	0.52	0.00	0.00	0.29	0.71	0.00	0.00	F1	P	BvAP019241	F	4/12/17	282	2.46E+01	1						
Juvenile	YOY_AP020182	0.00	1.00	0.00	1.00	0.00	0.00	0.00	0.00	LAND	P												
Juvenile	YOY_AP020183	0.00	1.00	0.00	1.00	0.00	0.00	0.00	0.00	LAND	P												
Juvenile	YOY_AP020184	0.00	1.00	0.00	1.00	0.00	0.00	0.00	0.00	LAND	P												
Juvenile	YOY_AP020185	0.00	1.00	0.00	1.00	0.00	0.00	0.00	0.00	LAND	P												
Juvenile	YOY_AP020186	0.00	1.00	0.00	1.00	0.00	0.00	0.00	0.00	LAND	P												
Juvenile	YOY_AP020187	0.00	1.00	0.00	1.00	0.00	0.00	0.00	0.00	LAND	P												
Juvenile	YOY_AP020188	0.00	1.00	0.00	1.00	0.00	0.00	0.00	0.00	LAND	P												
Juvenile	YOY_AP020189	0.00	1.00	0.00	1.00	0.00	0.00	0.00	0.00	LAND	P												
Juvenile	YOY_AP020190	0.00	1.00	0.00	1.00	0.00	0.00	0.00	0.00	LAND	P												
Juvenile	YOY_AP020191	1.00	0.00	1.00	0.00	0.00	0.00	0.00	0.00	ANAD	P	BvAP018677	M	4/12/17	269	3.76E+01	1	?	?	?	?	?	1
Juvenile	YOY_AP020527	1.00	0.00	1.00	0.00	0.00	0.00	0.00	0.00	ANAD	P	BvAP019060	F	4/12/17	269	3.72E+01	1	BvAP017951	M	4/12/17	272	4.15E+01	1
Juvenile	YOY_AP020193	0.00	1.00	0.00	1.00	0.00	0.00	0.00	0.00	LAND	P												
Juvenile	YOY_AP020194	0.00	1.00	0.00	1.00	0.00	0.00	0.00	0.00	LAND	P												
Juvenile	YOY_AP020195	0.00	1.00	0.00	1.00	0.00	0.00	0.00	0.00	LAND	P												
Juvenile	YOY_AP020196	0.00	1.00	0.00	1.00	0.00	0.00	0.00	0.00	LAND	P												
Juvenile	YOY_AP020197	0.00	1.00	0.00	1.00	0.00	0.00	0.00	0.00	LAND	P												
Juvenile	YOY_AP020198	0.00	1.00	0.00	1.00	0.00	0.00	0.00	0.00	LAND	P												
Juvenile	YOY_AP020199	0.99	0.01	1.00	0.00	0.00	0.00	0.00	0.00	ANAD	P	BvAP017870	F	4/12/17	256	3.85E+01	1	BvAP017670	M	3/30/17	264	4.12E+01	1
Juvenile	YOY_AP020200	0.00	1.00	0.00	1.00	0.00	0.00	0.00	0.00	LAND	P												
Juvenile	YOY_AP020201	0.00	1.00	0.00	1.00	0.00	0.00	0.00	0.00	LAND	P												
Juvenile	YOY_AP020202	0.00	1.00	0.00	1.00	0.00	0.00	0.00	0.00	LAND	P												
Juvenile	YOY_AP020203	0.28	0.72	0.00	0.00	0.00	0.01	0.00	0.99	LAND_BC	P												
Juvenile	YOY_AP020205	0.00	1.00	0.00	1.00	0.00	0.00	0.00	0.00	LAND	P												
Juvenile	YOY_AP020206	0.00	1.00	0.00	1.00	0.00	0.00	0.00	0.00	LAND	P												
Juvenile	YOY_AP020207	0.00	1.00	0.00	1.00	0.00	0.00	0.00	0.00	LAND	P												
Juvenile	YOY_AP020208	0.00	1.00	0.00	1.00	0.00	0.00	0.00	0.00	LAND	P												
Juvenile	YOY_AP020209	0.00	1.00	0.00	1.00	0.00	0.00	0.00	0.00	LAND	P												
Juvenile	YOY_AP020210	0.00	1.00	0.00	1.00	0.00	0.00	0.00	0.00	LAND	P												
Juvenile	YOY_AP020211	0.00	1.00	0.00	1.00	0.00	0.00	0.00	0.00	LAND	P												
Juvenile	YOY_AP020212	0.00	1.00	0.00	1.00	0.00	0.00	0.00	0.00	LAND	P												
Juvenile	YOY_AP020213	0.00	1.00	0.00	1.00	0.00	0.00	0.00	0.00	LAND	P												

Juvenile	YOY_AP020214	0.00	1.00	0.00	1.00	0.00	0.00	0.00	0.00	LAND	P												
Juvenile	YOY_AP020215	0.00	1.00	0.00	1.00	0.00	0.00	0.00	0.00	LAND	P												
Juvenile	YOY_AP020216	0.00	1.00	0.00	1.00	0.00	0.00	0.00	0.00	LAND	P												
Juvenile	YOY_AP020217	0.00	1.00	0.00	1.00	0.00	0.00	0.00	0.00	LAND	P												
Juvenile	YOY_AP020218	0.00	1.00	0.00	1.00	0.00	0.00	0.00	0.00	LAND	P												
Juvenile	YOY_AP020219	0.00	1.00	0.00	1.00	0.00	0.00	0.00	0.00	LAND	P												
Juvenile	YOY_AP020220	0.00	1.00	0.00	1.00	0.00	0.00	0.00	0.00	LAND	P												
Juvenile	YOY_AP020221	0.00	1.00	0.00	1.00	0.00	0.00	0.00	0.00	LAND	P												
Juvenile	YOY_AP020222	0.00	1.00	0.00	1.00	0.00	0.00	0.00	0.00	LAND	P												
Juvenile	YOY_AP020223	0.00	1.00	0.00	1.00	0.00	0.00	0.00	0.00	LAND	P												
Juvenile	YOY_AP020224	0.00	1.00	0.00	1.00	0.00	0.00	0.00	0.00	LAND	P												
Juvenile	YOY_AP020225	0.00	1.00	0.00	1.00	0.00	0.00	0.00	0.00	LAND	P												
Juvenile	YOY_AP020226	0.00	1.00	0.00	1.00	0.00	0.00	0.00	0.00	LAND	P												
Juvenile	YOY_AP020227	0.00	1.00	0.00	1.00	0.00	0.00	0.00	0.00	LAND	P												
Juvenile	YOY_AP020228	0.00	1.00	0.00	1.00	0.00	0.00	0.00	0.00	LAND	P												
Juvenile	YOY_AP020229	0.00	1.00	0.00	1.00	0.00	0.00	0.00	0.00	LAND	P												
Juvenile	YOY_AP020230	0.00	1.00	0.00	1.00	0.00	0.00	0.00	0.00	LAND	P												
Juvenile	YOY_AP020231	0.00	1.00	0.00	1.00	0.00	0.00	0.00	0.00	LAND	P												
Juvenile	YOY_AP020232	0.00	1.00	0.00	1.00	0.00	0.00	0.00	0.00	LAND	P												
Juvenile	YOY_AP020233	0.00	1.00	0.00	1.00	0.00	0.00	0.00	0.00	LAND	P												
Juvenile	YOY_AP020234	0.00	1.00	0.00	1.00	0.00	0.00	0.00	0.00	LAND	P												
Juvenile	YOY_AP020235	0.00	1.00	0.00	1.00	0.00	0.00	0.00	0.00	LAND	P												
Juvenile	YOY_AP020236	0.26	0.74	0.00	0.00	0.00	0.00	0.00	1.00	LAND_BC	P												
Juvenile	YOY_AP020237	0.00	1.00	0.00	1.00	0.00	0.00	0.00	0.00	LAND	P												
Juvenile	YOY_AP020238	0.00	1.00	0.00	1.00	0.00	0.00	0.00	0.00	LAND	P												
Juvenile	YOY_AP020239	0.00	1.00	0.00	1.00	0.00	0.00	0.00	0.00	LAND	P												
Juvenile	YOY_AP020240	0.00	1.00	0.00	1.00	0.00	0.00	0.00	0.00	LAND	P												
Juvenile	YOY_AP020241	0.00	1.00	0.00	1.00	0.00	0.00	0.00	0.00	LAND	P												
Juvenile	YOY_AP020242	0.00	1.00	0.00	1.00	0.00	0.00	0.00	0.00	LAND	P												
Juvenile	YOY_AP020243	0.00	1.00	0.00	1.00	0.00	0.00	0.00	0.00	LAND	P												
Juvenile	YOY_AP020244	0.00	1.00	0.00	1.00	0.00	0.00	0.00	0.00	LAND	P												
Juvenile	YOY_AP020245	0.00	1.00	0.00	1.00	0.00	0.00	0.00	0.00	LAND	P												
Juvenile	YOY_AP020246	0.00	1.00	0.00	1.00	0.00	0.00	0.00	0.00	LAND	P												
Juvenile	YOY_AP020247	0.00	1.00	0.00	1.00	0.00	0.00	0.00	0.00	LAND	P												
Juvenile	YOY_AP020248	0.00	1.00	0.00	1.00	0.00	0.00	0.00	0.00	LAND	P												
Juvenile	YOY_AP020249	0.00	1.00	0.00	1.00	0.00	0.00	0.00	0.00	LAND	P												
Juvenile	YOY_AP020250	0.00	1.00	0.00	1.00	0.00	0.00	0.00	0.00	LAND	P												
Juvenile	YOY_AP020251	0.00	1.00	0.00	1.00	0.00	0.00	0.00	0.00	LAND	P												
Juvenile	YOY_AP020252	0.00	1.00	0.00	1.00	0.00	0.00	0.00	0.00	LAND	P												
Juvenile	YOY_AP020253	0.00	1.00	0.00	1.00	0.00	0.00	0.00	0.00	LAND	P												
Juvenile	YOY_AP020254	0.00	1.00	0.00	1.00	0.00	0.00	0.00	0.00	LAND	P												
Juvenile	YOY_AP020255	0.00	1.00	0.00	1.00	0.00	0.00	0.00	0.00	LAND	P												
Juvenile	YOY_AP020256	0.00	1.00	0.00	1.00	0.00	0.00	0.00	0.00	LAND	P												
Juvenile	YOY_AP020257	0.00	1.00	0.00	1.00	0.00	0.00	0.00	0.00	LAND	P												
Juvenile	YOY_AP020258	0.00	1.00	0.00	1.00	0.00	0.00	0.00	0.00	LAND	P												
Juvenile	YOY_AP020259	0.00	1.00	0.00	1.00	0.00	0.00	0.00	0.00	LAND	P												
Juvenile	YOY_AP020570	1.00	0.00	1.00	0.00	0.00	0.00	0.00	0.00	ANAD	P	BvAP019278	M	4/12/17	281	3.00E+01	1	BvAP018628	F	4/12/17	281	4.21E+01	1
Juvenile	YOY_AP020526	0.65	0.35	0.00	0.00	0.00	0.98	0.02	0.00	F1	P	BvAP018570	F	4/12/17	272	3.18E+01	1						
Juvenile	YOY_AP020262	0.00	1.00	0.00	1.00	0.00	0.00	0.00	0.00	LAND	P												
Juvenile	YOY_AP020263	0.00	1.00	0.00	1.00	0.00	0.00	0.00	0.00	LAND	P												
Juvenile	YOY_AP020264	0.00	1.00	0.00	1.00	0.00	0.00	0.00	0.00	LAND	P												
Juvenile	YOY_AP020265	0.00	1.00	0.00	1.00	0.00	0.00	0.00	0.00	LAND	P												
Juvenile	YOY_AP020266	0.00	1.00	0.00	1.00	0.00	0.00	0.00	0.00	LAND	P												
Juvenile	YOY_AP020267	0.00	1.00	0.00	1.00	0.00	0.00	0.00	0.00	LAND	P												
Juvenile	YOY_AP020271	0.00	1.00	0.00	1.00	0.00	0.00	0.00	0.00	LAND	P												
Juvenile	YOY_AP020272	0.00	1.00	0.00	1.00	0.00	0.00	0.00	0.00	LAND	P												
Juvenile	YOY_AP020273	0.00	1.00	0.00	1.00	0.00	0.00	0.00	0.00	LAND	P												
Juvenile	YOY_AP020274	0.00	1.00	0.00	1.00	0.00	0.00	0.00	0.00	LAND	P												
Juvenile	YOY_AP020275	0.00	1.00	0.00	1.00	0.00	0.00	0.00	0.00	LAND	P												
Juvenile	YOY_AP020277	0.00	1.00	0.00	1.00	0.00	0.00	0.00	0.00	LAND	P												
Juvenile	YOY_AP020278	0.00	1.00	0.00	1.00	0.00	0.00	0.00	0.00	LAND	P												
Juvenile	YOY_AP020279	0.00	1.00	0.00	1.00	0.00	0.00	0.00	0.00	LAND	P												
Juvenile	YOY_AP020280	0.00	1.00	0.00	1.00	0.00	0.00	0.00	0.00	LAND	P												
Juvenile	YOY_AP020281	0.00	1.00	0.00	1.00	0.00	0.00	0.00	0.00	LAND	P												
Juvenile	YOY_AP020282	1.00	0.00	1.00	0.00	0.00	0.00	0.00	0.00	ANAD	P	BvAP017463	F	3/30/17	269	4.38E+01	1	BvAP017366	M	3/30/17	282	4.99E+01	1
Juvenile	YOY_AP020283	0.00	1.00	0.00	1.00	0.00	0.00	0.00	0.00	LAND	P												
Juvenile	YOY_AP020284	0.00	1.00	0.00	1.00	0.00	0.00	0.00	0.00	LAND	P												
Juvenile	YOY_AP020285	0.00	1.00	0.00	1.00	0.00	0.00	0.00	0.00	LAND	P												
Juvenile	YOY_AP020633	1.00	0.00	1.00	0.00	0.00	0.00	0.00	0.00	ANAD	P	BvAP019014	M	4/12/17	235	4.46E+01	1	BvAP017879	F	4/12/17	266	5.56E+01	1
Juvenile	YOY_AP020287	0.00	1.00	0.00	1.00	0.00	0.00	0.00	0.00	LAND	P												
Juvenile	YOY_AP020288	0.00	1.00	0.00	1.00	0.00	0.00	0.00	0.00	LAND	P												
Juvenile	YOY_AP020289	0.00	1.00	0.00	1.00	0.00	0.00	0.00	0.00	LAND	P												
Juvenile	YOY_AP020290	1.00	0.00	1.00	0.00	0.00	0.00	0.00	0.00	ANAD	P	BvAP019404	F	4/19/17	266	2.48E+01	1	BvAP016899	M	3/30/17	259	3.65E+01	1
Juvenile	YOY_AP020291	0.00	1.00	0.00	1.00	0.00	0.00	0.00	0.00	LAND	P												
Juvenile	YOY_AP020292	0.00	1.00	0.00	1.00	0.00	0.00	0.00	0.00	LAND	P												
Juvenile	YOY_AP020293	0.00	1.00	0.00	1.00	0.00	0.00	0.00	0.00	LAND	P												
Juvenile	YOY_AP020294	0.00	1.00	0.00	1.00	0.00	0.00	0.00	0.00	LAND	P												
Juvenile	YOY_AP020295	0.31	0.69	0.00	0.00	0.00	0.00	0.00	1.00	LAND_BC	P												
Juvenile	YOY_AP020296	0.00	1.00	0.00	1.00	0.00	0.00	0.00	0.00	LAND	P												
Juvenile	YOY_AP020297	0.00	1.00	0.00	1.00	0.00	0.00	0.00	0.00	LAND	P												
Juvenile	YOY_AP020764	0.45	0.55	0.00	0.00	1.00	0.00	0.00	0.00	F1	P	BvAP018998	F	4/12/17	282	2.06E+01	1						
Juvenile	YOY_AP020299	0.00	1.00	0.00	1.00	0.00	0.00	0.00	0.00	LAND	P												
Juvenile	YOY_AP020300	0.00	1.00	0.00	1.00	0.00	0.00	0.00	0.00	LAND	P												
Juvenile	YOY_AP020301	0.00	1.00	0.00	1.00	0.00	0.00	0.00	0.00	LAND	P												
Juvenile	YOY_AP020302	0.00	1.00	0.00	1.00	0.00	0.00	0.00	0.00	LAND	P												
Juvenile	YOY_AP020303	0.00	1.00	0.00	1.00	0.00	0.00	0.00	0.00	LAND	P												
Juvenile	YOY_AP020304	0.00	1.00	0.00	1.00	0.00	0.00	0.00	0.00	LAND	P												
Juvenile	YOY_AP020305	0.00	1.00	0.00	1.00	0.00	0.00	0.00	0.00	LAND	P												
Juvenile	YOY_AP020306	0.00	1.00	0.00	1.00	0.00	0.00	0.00	0.00	LAND	P												
Juvenile	YOY_AP020307	0.00	1.00	0.00	1.00	0.00	0.00	0.00	0.00	LAND	P												
Juvenile	YOY_AP020308	0.00	1.00	0.00	1.00	0.00	0.00	0.00	0.00	LAND	P												
Juvenile	YOY_AP020309	0.00	1.00	0.00	1.00	0.00	0.00	0.00	0.00	LAND	P												
Juvenile	YOY_AP020310	0.00	1.00	0.00	1.00	0.00	0.00	0.00	0.00	LAND	P												
Juvenile	YOY_AP020311	0.00	1.00	0.00	1.00	0.00	0.00	0.00	0.00	LAND	P												
Juvenile	YOY_AP020312	0.00	1.00	0.00	1.00	0.00	0.00	0.00	0.00	LAND	P												
Juvenile	YOY_AP020313	0.00	1.00	0.00	1.00	0.00	0.00	0.00	0.00	LAND	P												
Juvenile	YOY_AP020314	0.00	1.00	0.00	1.00	0.00	0.00	0.00	0.00	LAND	P												
Juvenile	YOY_AP020315	0.00	1.00	0.00	1.00	0.00	0.00	0.00	0.00	LAND	P												
Juvenile	YOY_AP020316	0.00	1.00	0.00	1.00	0.00	0.00	0.00	0.00	LAND	P												
Juvenile	YOY_AP020317	0.00	1.00	0.00	1.00	0.00	0.00	0.00	0.00	LAND	P												
Juvenile	YOY_AP020318	0.00	1.00	0.00	1.00	0.00	0.00	0.00	0.00	LAND	P												

Juvenile	YOY_AP020319	0.00	1.00	0.00	1.00	0.00	0.00	0.00	0.00	LAND	P												
Juvenile	YOY_AP020320	0.00	1.00	0.00	1.00	0.00	0.00	0.00	0.00	LAND	P												
Juvenile	YOY_AP020321	0.00	1.00	0.00	1.00	0.00	0.00	0.00	0.00	LAND	P												
Juvenile	YOY_AP020322	0.00	1.00	0.00	1.00	0.00	0.00	0.00	0.00	LAND	P												
Juvenile	YOY_AP020323	0.00	1.00	0.00	1.00	0.00	0.00	0.00	0.00	LAND	P												
Juvenile	YOY_AP020324	0.00	1.00	0.00	1.00	0.00	0.00	0.00	0.00	LAND	P												
Juvenile	YOY_AP020325	0.00	1.00	0.00	1.00	0.00	0.00	0.00	0.00	LAND	P												
Juvenile	YOY_AP020326	0.00	1.00	0.00	1.00	0.00	0.00	0.00	0.00	LAND	P												
Juvenile	YOY_AP020327	0.00	1.00	0.00	1.00	0.00	0.00	0.00	0.00	LAND	P												
Juvenile	YOY_AP020328	0.00	1.00	0.00	1.00	0.00	0.00	0.00	0.00	LAND	P												
Juvenile	YOY_AP020329	0.00	1.00	0.00	1.00	0.00	0.00	0.00	0.00	LAND	P												
Juvenile	YOY_AP020330	0.00	1.00	0.00	1.00	0.00	0.00	0.00	0.00	LAND	P												
Juvenile	YOY_AP020331	0.00	1.00	0.00	1.00	0.00	0.00	0.00	0.00	LAND	P												
Juvenile	YOY_AP020332	0.00	1.00	0.00	1.00	0.00	0.00	0.00	0.00	LAND	P												
Juvenile	YOY_AP020333	0.00	1.00	0.00	1.00	0.00	0.00	0.00	0.00	LAND	P												
Juvenile	YOY_AP020334	0.00	1.00	0.00	1.00	0.00	0.00	0.00	0.00	LAND	P												
Juvenile	YOY_AP020335	0.00	1.00	0.00	1.00	0.00	0.00	0.00	0.00	LAND	P												
Juvenile	YOY_AP020336	0.00	1.00	0.00	1.00	0.00	0.00	0.00	0.00	LAND	P												
Juvenile	YOY_AP020337	0.00	1.00	0.00	1.00	0.00	0.00	0.00	0.00	LAND	P												
Juvenile	YOY_AP020338	0.00	1.00	0.00	1.00	0.00	0.00	0.00	0.00	LAND	P												
Juvenile	YOY_AP020339	0.00	1.00	0.00	1.00	0.00	0.00	0.00	0.00	LAND	P												
Juvenile	YOY_AP020340	0.00	1.00	0.00	1.00	0.00	0.00	0.00	0.00	LAND	P												
Juvenile	YOY_AP020341	0.00	1.00	0.00	1.00	0.00	0.00	0.00	0.00	LAND	P												
Juvenile	YOY_AP020342	0.00	1.00	0.00	1.00	0.00	0.00	0.00	0.00	LAND	P												
Juvenile	YOY_AP020343	0.00	1.00	0.00	1.00	0.00	0.00	0.00	0.00	LAND	P												
Juvenile	YOY_AP020345	0.00	1.00	0.00	1.00	0.00	0.00	0.00	0.00	LAND	P												
Juvenile	YOY_AP020346	0.00	1.00	0.00	1.00	0.00	0.00	0.00	0.00	LAND	P												
Juvenile	YOY_AP020347	0.00	1.00	0.00	1.00	0.00	0.00	0.00	0.00	LAND	P												
Juvenile	YOY_AP020348	0.00	1.00	0.00	1.00	0.00	0.00	0.00	0.00	LAND	P												
Juvenile	YOY_AP020349	0.00	1.00	0.00	1.00	0.00	0.00	0.00	0.00	LAND	P												
Juvenile	YOY_AP020350	0.00	1.00	0.00	1.00	0.00	0.00	0.00	0.00	LAND	P												
Juvenile	YOY_AP020351	0.00	1.00	0.00	1.00	0.00	0.00	0.00	0.00	LAND	P												
Juvenile	YOY_AP020352	0.00	1.00	0.00	1.00	0.00	0.00	0.00	0.00	LAND	P												
Juvenile	YOY_AP020807	0.50	0.50	0.00	0.00	1.00	0.00	0.00	0.00	F1	P	BvAP018796	M	4/12/17	259	1.26E+01	0.9904						
Juvenile	YOY_AP020354	0.00	1.00	0.00	1.00	0.00	0.00	0.00	0.00	LAND	P												
Juvenile	YOY_AP020355	0.00	1.00	0.00	1.00	0.00	0.00	0.00	0.00	LAND	P												
Juvenile	YOY_AP020356	0.00	1.00	0.00	1.00	0.00	0.00	0.00	0.00	LAND	P												
Juvenile	YOY_AP020357	0.00	1.00	0.00	1.00	0.00	0.00	0.00	0.00	LAND	P												
Juvenile	YOY_AP020675	1.00	0.00	1.00	0.00	0.00	0.00	0.00	0.00	ANAD	P	BvAP018810	M	4/12/17	266	4.84E+01	1	BvAP018646	M	4/12/17	254	4.30E+01	1
Juvenile	YOY_AP020359	0.00	1.00	0.00	1.00	0.00	0.00	0.00	0.00	LAND	P												
Juvenile	YOY_AP020360	0.00	1.00	0.00	1.00	0.00	0.00	0.00	0.00	LAND	P												
Juvenile	YOY_AP020361	0.00	1.00	0.00	1.00	0.00	0.00	0.00	0.00	LAND	P												
Juvenile	YOY_AP020362	0.28	0.73	0.00	0.00	0.00	0.00	0.00	1.00	LAND_BC	P												
Juvenile	YOY_AP020363	0.00	1.00	0.00	1.00	0.00	0.00	0.00	0.00	LAND	P												
Juvenile	YOY_AP020364	0.00	1.00	0.00	1.00	0.00	0.00	0.00	0.00	LAND	P												
Juvenile	YOY_AP020365	0.00	1.00	0.00	1.00	0.00	0.00	0.00	0.00	LAND	P												
Juvenile	YOY_AP020697	1.00	0.00	1.00	0.00	0.00	0.00	0.00	0.00	ANAD	P	BvAP018994	F	4/12/17	291	4.31E+01	1	BvAP018799	M	4/12/17	268	3.56E+01	1
Juvenile	YOY_AP020367	0.00	1.00	0.00	1.00	0.00	0.00	0.00	0.00	LAND	P												
Juvenile	YOY_AP020368	0.00	1.00	0.00	1.00	0.00	0.00	0.00	0.00	LAND	P												
Juvenile	YOY_AP020369	0.00	1.00	0.00	1.00	0.00	0.00	0.00	0.00	LAND	P												
Juvenile	YOY_AP020370	0.00	1.00	0.00	1.00	0.00	0.00	0.00	0.00	LAND	P												
Juvenile	YOY_AP020371	0.00	1.00	0.00	1.00	0.00	0.00	0.00	0.00	LAND	P												
Juvenile	YOY_AP020372	0.00	1.00	0.00	1.00	0.00	0.00	0.00	0.00	LAND	P												
Juvenile	YOY_AP020373	0.00	1.00	0.00	1.00	0.00	0.00	0.00	0.00	LAND	P												
Juvenile	YOY_AP020374	0.00	1.00	0.00	1.00	0.00	0.00	0.00	0.00	LAND	P												
Juvenile	YOY_AP020375	0.00	1.00	0.00	1.00	0.00	0.00	0.00	0.00	LAND	P												
Juvenile	YOY_AP020376	0.00	1.00	0.00	1.00	0.00	0.00	0.00	0.00	LAND	P												
Juvenile	YOY_AP020377	0.00	1.00	0.00	1.00	0.00	0.00	0.00	0.00	LAND	P												
Juvenile	YOY_AP020378	0.00	1.00	0.00	1.00	0.00	0.00	0.00	0.00	LAND	P												
Juvenile	YOY_AP020379	0.00	1.00	0.00	1.00	0.00	0.00	0.00	0.00	LAND	P												
Juvenile	YOY_AP020380	0.00	1.00	0.00	1.00	0.00	0.00	0.00	0.00	LAND	P												
Juvenile	YOY_AP020709	1.00	0.00	1.00	0.00	0.00	0.00	0.00	0.00	ANAD	P	BvAP019037	M	4/12/17	243	3.81E+01	1	BvAP018649	F	4/12/17	271	4.60E+01	1
Juvenile	YOY_AP020382	0.00	1.00	0.00	1.00	0.00	0.00	0.00	0.00	LAND	P												
Juvenile	YOY_AP020383	0.00	1.00	0.00	1.00	0.00	0.00	0.00	0.00	LAND	P												
Juvenile	YOY_AP020384	0.00	1.00	0.00	1.00	0.00	0.00	0.00	0.00	LAND	P												
Juvenile	YOY_AP020385	0.00	1.00	0.00	1.00	0.00	0.00	0.00	0.00	LAND	P												
Juvenile	YOY_AP020386	0.00	1.00	0.00	1.00	0.00	0.00	0.00	0.00	LAND	P												
Juvenile	YOY_AP020387	0.00	1.00	0.00	1.00	0.00	0.00	0.00	0.00	LAND	P												
Juvenile	YOY_AP020388	0.00	1.00	0.00	1.00	0.00	0.00	0.00	0.00	LAND	P												
Juvenile	YOY_AP020389	0.00	1.00	0.00	1.00	0.00	0.00	0.00	0.00	LAND	P												
Juvenile	YOY_AP020390	0.00	1.00	0.00	1.00	0.00	0.00	0.00	0.00	LAND	P												
Juvenile	YOY_AP020391	0.00	1.00	0.00	1.00	0.00	0.00	0.00	0.00	LAND	P												
Juvenile	YOY_AP020392	0.00	1.00	0.00	1.00	0.00	0.00	0.00	0.00	LAND	P												
Juvenile	YOY_AP020393	0.00	1.00	0.00	1.00	0.00	0.00	0.00	0.00	LAND	P												
Juvenile	YOY_AP020394	0.00	1.00	0.00	1.00	0.00	0.00	0.00	0.00	LAND	P												
Juvenile	YOY_AP020395	0.00	1.00	0.00	1.00	0.00	0.00	0.00	0.00	LAND	P												
Juvenile	YOY_AP020396	0.00	1.00	0.00	1.00	0.00	0.00	0.00	0.00	LAND	P												
Juvenile	YOY_AP020397	0.00	1.00	0.00	1.00	0.00	0.00	0.00	0.00	LAND	P												
Juvenile	YOY_AP020398	0.00	1.00	0.00	1.00	0.00	0.00	0.00	0.00	LAND	P												
Juvenile	YOY_AP020399	0.00	1.00	0.00	1.00	0.00	0.00	0.00	0.00	LAND	P												
Juvenile	YOY_AP020400	0.00	1.00	0.00	1.00	0.00	0.00	0.00	0.00	LAND	P												
Juvenile	YOY_AP020868	0.46	0.54	0.00	0.00	0.53	0.47	0.00	0.01	F1	P	BvAP017997	F	4/12/17	269	2.42E+01	1						
Juvenile	YOY_AP020402	0.00	1.00	0.00	1.00	0.00	0.00	0.00	0.00	LAND	P												
Juvenile	YOY_AP020403	0.00	1.00	0.00	1.00	0.00	0.00	0.00	0.00	LAND	P												
Juvenile	YOY_AP020404	0.00	1.00	0.00	1.00	0.00	0.00	0.00	0.00	LAND	P												
Juvenile	YOY_AP020405	0.27	0.73	0.00	0.00	0.00	0.00	0.00	1.00	LAND_BC	P												
Juvenile	YOY_AP020406	0.29	0.71	0.00	0.00	0.00	0.00	0.00	1.00	LAND_BC	P												
Juvenile	YOY_AP020407	0.00	1.00	0.00	1.00	0.00	0.00	0.00	0.00	LAND	P												
Juvenile	YOY_AP020408	0.00	1.00	0.00	1.00	0.00	0.00	0.00	0.00	LAND	P												
Juvenile	YOY_AP020409	0.00	1.00	0.00	1.00	0.00	0.00	0.00	0.00	LAND	P												
Juvenile	YOY_AP020410	0.00	1.00	0.00	1.00	0.00	0.00	0.00	0.00	LAND	P												
Juvenile	YOY_AP020411	0.00	1.00	0.00	1.00	0.00	0.00	0.00	0.00	LAND	P												
Juvenile	YOY_AP020412	0.16	0.84	0.00	0.00	0.00	0.00	0.00	1.00	LAND_BC	P												
Juvenile	YOY_AP020413	1.00	0.00	1.00	0.00	0.00	0.00	0.00	0.00	ANAD	P	BvAP017369	F	3/30/17	294	5.29E+01	1	BvAP016955	M	3/30/17	277	4.97E+01	1
Juvenile	YOY_AP020414	0.00	1.00	0.00	1.00	0.00	0.00	0.00	0.00	LAND	P												
Juvenile	YOY_AP020415	0.00	1.00	0.00	1.00	0.00	0.00	0.00	0.00	LAND	P												
Juvenile	YOY_AP020416	0.00	1.00	0.00	1.00	0.00	0.00	0.00	0.00	LAND	P												
Juvenile	YOY_AP020417	0.00	1.00	0.00	1.00	0.00	0.00	0.00	0.00	LAND	P												
Juvenile	YOY_AP020418	0.00	1.00	0.00	1.00	0.00	0.00	0.00	0.00	LAND	P												
Juvenile	YOY_AP020419	0.00	1.00	0.00	1.00	0.00	0.00	0.00	0.00	LAND	P												
Juvenile	YOY_AP020420	0.00	1.00	0.00	1.00	0.00	0.00	0.00	0.00	LAND	P												

Juvenile	YOY_AP020421	0.00	1.00	0.00	1.00	0.00	0.00	0.00	0.00	LAND	P												
Juvenile	YOY_AP020422	0.00	1.00	0.00	1.00	0.00	0.00	0.00	0.00	LAND	P												
Juvenile	YOY_AP020423	0.00	1.00	0.00	1.00	0.00	0.00	0.00	0.00	LAND	P												
Juvenile	YOY_AP020424	0.00	1.00	0.00	1.00	0.00	0.00	0.00	0.00	LAND	P												
Juvenile	YOY_AP020425	0.00	1.00	0.00	1.00	0.00	0.00	0.00	0.00	LAND	P												
Juvenile	YOY_AP020426	0.00	1.00	0.00	1.00	0.00	0.00	0.00	0.00	LAND	P												
Juvenile	YOY_AP020427	0.00	1.00	0.00	1.00	0.00	0.00	0.00	0.00	LAND	P												
Juvenile	YOY_AP020428	0.00	1.00	0.00	1.00	0.00	0.00	0.00	0.00	LAND	P												
Juvenile	YOY_AP019522	0.44	0.56	0.00	0.00	0.00	1.00	0.00	0.00	F1	P	BvAP019187	M	4/19/17	255	3.68E+01	1						
Juvenile	YOY_AP020430	0.00	1.00	0.00	1.00	0.00	0.00	0.00	0.00	LAND	P												
Juvenile	YOY_AP020431	0.00	1.00	0.00	1.00	0.00	0.00	0.00	0.00	LAND	P												
Juvenile	YOY_AP020432	0.00	1.00	0.00	1.00	0.00	0.00	0.00	0.00	LAND	P												
Juvenile	YOY_AP020433	0.00	1.00	0.00	1.00	0.00	0.00	0.00	0.00	LAND	P												
Juvenile	YOY_AP020434	0.00	1.00	0.00	1.00	0.00	0.00	0.00	0.00	LAND	P												
Juvenile	YOY_AP020435	0.00	1.00	0.00	1.00	0.00	0.00	0.00	0.00	LAND	P												
Juvenile	YOY_AP020436	0.00	1.00	0.00	1.00	0.00	0.00	0.00	0.00	LAND	P												
Juvenile	YOY_AP020437	0.00	1.00	0.00	1.00	0.00	0.00	0.00	0.00	LAND	P												
Juvenile	YOY_AP020438	0.00	1.00	0.00	1.00	0.00	0.00	0.00	0.00	LAND	P												
Juvenile	YOY_AP020439	0.00	1.00	0.00	1.00	0.00	0.00	0.00	0.00	LAND	P												
Juvenile	YOY_AP019530	0.57	0.43	0.00	0.00	0.68	0.30	0.01	0.00	F1	P	BvAP018329	M	4/19/17	255	3.34E+01	1						
Juvenile	YOY_AP020441	0.00	1.00	0.00	1.00	0.00	0.00	0.00	0.00	LAND	P												
Juvenile	YOY_AP020442	0.00	1.00	0.00	1.00	0.00	0.00	0.00	0.00	LAND	P												
Juvenile	YOY_AP020443	0.00	1.00	0.00	1.00	0.00	0.00	0.00	0.00	LAND	P												
Juvenile	YOY_AP020444	0.00	1.00	0.00	1.00	0.00	0.00	0.00	0.00	LAND	P												
Juvenile	YOY_AP020445	0.00	1.00	0.00	1.00	0.00	0.00	0.00	0.00	LAND	P												
Juvenile	YOY_AP019546	0.47	0.53	0.00	0.00	1.00	0.00	0.00	0.00	F1	P	BvAP018919	M	4/19/17	272	2.55E+01	1						
Juvenile	YOY_AP020447	0.00	1.00	0.00	1.00	0.00	0.00	0.00	0.00	LAND	P												
Juvenile	YOY_AP020448	0.00	1.00	0.00	1.00	0.00	0.00	0.00	0.00	LAND	P												
Juvenile	YOY_AP020449	0.00	1.00	0.00	1.00	0.00	0.00	0.00	0.00	LAND	P												
Juvenile	YOY_AP020450	0.00	1.00	0.00	1.00	0.00	0.00	0.00	0.00	LAND	P												
Juvenile	YOY_AP020451	0.00	1.00	0.00	1.00	0.00	0.00	0.00	0.00	LAND	P												
Juvenile	YOY_AP020452	0.00	1.00	0.00	1.00	0.00	0.00	0.00	0.00	LAND	P												
Juvenile	YOY_AP020453	0.00	1.00	0.00	1.00	0.00	0.00	0.00	0.00	LAND	P												
Juvenile	YOY_AP020454	0.00	1.00	0.00	1.00	0.00	0.00	0.00	0.00	LAND	P												
Juvenile	YOY_AP020455	0.00	1.00	0.00	1.00	0.00	0.00	0.00	0.00	LAND	P												
Juvenile	YOY_AP020456	0.00	1.00	0.00	1.00	0.00	0.00	0.00	0.00	LAND	P												
Juvenile	YOY_AP020755	1.00	0.00	1.00	0.00	0.00	0.00	0.00	0.00	ANAD	P	BvAP019334	M	4/12/17	279	4.70E+01	1	BvAP018488	F	4/19/17	259	3.93E+01	1
Juvenile	YOY_AP020458	0.00	1.00	0.00	1.00	0.00	0.00	0.00	0.00	LAND	P												
Juvenile	YOY_AP020459	0.00	1.00	0.00	1.00	0.00	0.00	0.00	0.00	LAND	P												
Juvenile	YOY_AP020460	0.19	0.81	0.00	0.00	0.00	0.00	0.00	1.00	LAND_BC	P												
Juvenile	YOY_AP020461	0.00	1.00	0.00	1.00	0.00	0.00	0.00	0.00	LAND	P												
Juvenile	YOY_AP020462	0.00	1.00	0.00	1.00	0.00	0.00	0.00	0.00	LAND	P												
Juvenile	YOY_AP020463	0.00	1.00	0.00	1.00	0.00	0.00	0.00	0.00	LAND	P												
Juvenile	YOY_AP020464	0.00	1.00	0.00	1.00	0.00	0.00	0.00	0.00	LAND	P												
Juvenile	YOY_AP020465	0.00	1.00	0.00	1.00	0.00	0.00	0.00	0.00	LAND	P												
Juvenile	YOY_AP020467	1.00	0.00	1.00	0.00	0.00	0.00	0.00	0.00	ANAD	P	BvAP018686	M	4/12/17	251	4.44E+01	1	BvAP017424	M	3/30/17	267	2.41E+01	1
Juvenile	YOY_AP020468	0.00	1.00	0.00	1.00	0.00	0.00	0.00	0.00	LAND	P												
Juvenile	YOY_AP020469	0.00	1.00	0.00	1.00	0.00	0.00	0.00	0.00	LAND	P												
Juvenile	YOY_AP020470	0.00	1.00	0.00	1.00	0.00	0.00	0.00	0.00	LAND	P												
Juvenile	YOY_AP020471	0.00	1.00	0.00	1.00	0.00	0.00	0.00	0.00	LAND	P												
Juvenile	YOY_AP020472	0.00	1.00	0.00	1.00	0.00	0.00	0.00	0.00	LAND	P												
Juvenile	YOY_AP020473	0.00	1.00	0.00	1.00	0.00	0.00	0.00	0.00	LAND	P												
Juvenile	YOY_AP020474	0.00	1.00	0.00	1.00	0.00	0.00	0.00	0.00	LAND	P												
Juvenile	YOY_AP020475	0.00	1.00	0.00	1.00	0.00	0.00	0.00	0.00	LAND	P												
Juvenile	YOY_AP020476	0.00	1.00	0.00	1.00	0.00	0.00	0.00	0.00	LAND	P												
Juvenile	YOY_AP020477	0.00	1.00	0.00	1.00	0.00	0.00	0.00	0.00	LAND	P												
Juvenile	YOY_AP020478	0.00	1.00	0.00	1.00	0.00	0.00	0.00	0.00	LAND	P												
Juvenile	YOY_AP020479	0.00	1.00	0.00	1.00	0.00	0.00	0.00	0.00	LAND	P												
Juvenile	YOY_AP020480	0.00	1.00	0.00	1.00	0.00	0.00	0.00	0.00	LAND	P												
Juvenile	YOY_AP020481	1.00	0.00	1.00	0.00	0.00	0.00	0.00	0.00	ANAD	P	BvAP018414	M	4/19/17	266	5.03E+01	1	BvAP017369	F	3/30/17	294	4.83E+01	1
Juvenile	YOY_AP020482	0.00	1.00	0.00	1.00	0.00	0.00	0.00	0.00	LAND	P												
Juvenile	YOY_AP020483	0.00	1.00	0.00	1.00	0.00	0.00	0.00	0.00	LAND	P												
Juvenile	YOY_AP020484	0.00	1.00	0.00	1.00	0.00	0.00	0.00	0.00	LAND	P												
Juvenile	YOY_AP020485	0.00	1.00	0.00	1.00	0.00	0.00	0.00	0.00	LAND	P												
Juvenile	YOY_AP020486	1.00	0.00	1.00	0.00	0.00	0.00	0.00	0.00	ANAD	P	BvAP019010	F	4/12/17	253	3.62E+01	1	BvAP017081	M	3/30/17	250	3.30E+01	1
Juvenile	YOY_AP020487	0.00	1.00	0.00	1.00	0.00	0.00	0.00	0.00	LAND	P												
Juvenile	YOY_AP020488	0.00	1.00	0.00	1.00	0.00	0.00	0.00	0.00	LAND	P												
Juvenile	YOY_AP020489	0.00	1.00	0.00	1.00	0.00	0.00	0.00	0.00	LAND	P												
Juvenile	YOY_AP020490	1.00	0.00	1.00	0.00	0.00	0.00	0.00	0.00	ANAD	P	BvAP018805	M	4/12/17	257	3.19E+01	1	BvAP017684	F	3/30/17	290	5.25E+01	1
Juvenile	YOY_AP020491	0.00	1.00	0.00	1.00	0.00	0.00	0.00	0.00	LAND	P												
Juvenile	YOY_AP020492	0.00	1.00	0.00	1.00	0.00	0.00	0.00	0.00	LAND	P												
Juvenile	YOY_AP020493	0.00	1.00	0.00	1.00	0.00	0.00	0.00	0.00	LAND	P												
Juvenile	YOY_AP019761	0.56	0.44	0.00	0.00	1.00	0.00	0.00	0.00	F1	P	BvAP018052	F	4/19/17	286	3.43E+01	1						
Juvenile	YOY_AP020495	0.00	1.00	0.00	1.00	0.00	0.00	0.00	0.00	LAND	P												
Juvenile	YOY_AP020496	0.00	1.00	0.00	1.00	0.00	0.00	0.00	0.00	LAND	P												
Juvenile	YOY_AP020497	0.00	1.00	0.00	1.00	0.00	0.00	0.00	0.00	LAND	P												
Juvenile	YOY_AP020498	0.00	1.00	0.00	1.00	0.00	0.00	0.00	0.00	LAND	P												
Juvenile	YOY_AP020499	0.00	1.00	0.00	1.00	0.00	0.00	0.00	0.00	LAND	P												
Juvenile	YOY_AP020500	0.00	1.00	0.00	1.00	0.00	0.00	0.00	0.00	LAND	P												
Juvenile	YOY_AP020501	0.00	1.00	0.00	1.00	0.00	0.00	0.00	0.00	LAND	P												
Juvenile	YOY_AP020502	0.00	1.00	0.00	1.00	0.00	0.00	0.00	0.00	LAND	P												
Juvenile	YOY_AP020503	0.00	1.00	0.00	1.00	0.00	0.00	0.00	0.00	LAND	P												
Juvenile	YOY_AP020504	0.00	1.00	0.00	1.00	0.00	0.00	0.00	0.00	LAND	P												
Juvenile	YOY_AP020505	0.00	1.00	0.00	1.00	0.00	0.00	0.00	0.00	LAND	P												
Juvenile	YOY_AP020506	0.00	1.00	0.00	1.00	0.00	0.00	0.00	0.00	LAND	P												
Juvenile	YOY_AP020756	1.00	0.00	1.00	0.00	0.00	0.00	0.00	0.00	ANAD	P	BvAP018792	M	4/12/17	261	2.58E+01	1	BvAP017947	F	4/12/17	274	3.02E+01	1
Juvenile	YOY_AP019924	0.48	0.53	0.00	0.00	0.00	1.00	0.00	0.00	F1	P	BvAP018494	M	4/19/17	258	2.77E+01	1						
Juvenile	YOY_AP020509	0.00	1.00	0.00	1.00	0.00	0.00	0.00	0.00	LAND	P												
Juvenile	YOY_AP020510	0.00	1.00	0.00	1.00	0.00	0.00	0.00	0.00	LAND	P												
Juvenile	YOY_AP020511	0.00	1.00	0.00	1.00	0.00	0.00	0.00	0.00	LAND	P												
Juvenile	YOY_AP020512	0.00	1.00	0.00	1.00	0.00	0.00	0.00	0.00	LAND	P												
Juvenile	YOY_AP020513	0.00	1.00	0.00	1.00	0.00	0.00	0.00	0.00	LAND	P												
Juvenile	YOY_AP020514	0.00	1.00	0.00	1.00	0.00	0.00	0.00	0.00	LAND	P												
Juvenile	YOY_AP020515	0.00	1.00	0.00	1.00	0.00	0.00	0.00	0.00	LAND	P												
Juvenile	YOY_AP020516	0.00	1.00	0.00	1.00	0.00	0.00	0.00	0.00	LAND	P												
Juvenile	YOY_AP020517	0.00	1.00	0.00	1.00	0.00	0.00	0.00	0.00	LAND	P												
Juvenile	YOY_AP020518	0.00	1.00	0.00	1.00	0.00	0.00	0.00	0.00	LAND	P												
Juvenile	YOY_AP020519	0.00	1.00	0.00	1.00	0.00	0.00	0.00	0.00	LAND	P												
Juvenile	YOY_AP020520	0.00	1.00	0.00	1.00	0.00	0.00	0.00	0.00	LAND	P												
Juvenile	YOY_AP020521	0.00	1.00	0.00	1.00	0.00	0.00	0.00	0.00	LAND	P												
Juvenile	YOY_AP020522	0.00	1.00	0.00	1.00	0.00	0.00	0.00	0.00	LAND	P												

Juvenile	YOY_AP020523	0.00	1.00	0.00	1.00	0.00	0.00	0.00	0.00	LAND	P												
Juvenile	YOY_AP020524	0.00	1.00	0.00	1.00	0.00	0.00	0.00	0.00	LAND	P												
Juvenile	YOY_AP020525	0.00	1.00	0.00	1.00	0.00	0.00	0.00	0.00	LAND	P												
Juvenile	YOY_AP020023	0.55	0.45	0.00	0.00	1.00	0.00	0.00	0.00	F1	P	BvAP018285	F	4/19/17	265	4.67E+01	1						
Juvenile	YOY_AP020770	0.99	0.01	1.00	0.00	0.00	0.00	0.00	0.00	ANAD	P	BvAP019287	M	4/12/17	274	4.22E+01	1	BvAP017776	F	4/12/17	286	4.13E+01	1
Juvenile	YOY_AP020528	0.00	1.00	0.00	1.00	0.00	0.00	0.00	0.00	LAND	P												
Juvenile	YOY_AP020529	0.00	1.00	0.00	1.00	0.00	0.00	0.00	0.00	LAND	P												
Juvenile	YOY_AP020530	0.00	1.00	0.00	1.00	0.00	0.00	0.00	0.00	LAND	P												
Juvenile	YOY_AP020531	1.00	0.00	1.00	0.00	0.00	0.00	0.00	0.00	ANAD	P	BvAP018432	F	4/19/17	259	4.67E+01	1	BvAP017114	M	3/30/17	270	4.44E+01	1
Juvenile	YOY_AP020532	0.00	1.00	0.00	1.00	0.00	0.00	0.00	0.00	LAND	P												
Juvenile	YOY_AP020533	0.00	1.00	0.00	1.00	0.00	0.00	0.00	0.00	LAND	P												
Juvenile	YOY_AP020534	0.00	1.00	0.00	1.00	0.00	0.00	0.00	0.00	LAND	P												
Juvenile	YOY_AP020535	0.00	1.00	0.00	1.00	0.00	0.00	0.00	0.00	LAND	P												
Juvenile	YOY_AP020537	0.00	1.00	0.00	1.00	0.00	0.00	0.00	0.00	LAND	P												
Juvenile	YOY_AP020538	0.00	1.00	0.00	1.00	0.00	0.00	0.00	0.00	LAND	P												
Juvenile	YOY_AP020539	0.00	1.00	0.00	1.00	0.00	0.00	0.00	0.00	LAND	P												
Juvenile	YOY_AP020540	0.00	1.00	0.00	1.00	0.00	0.00	0.00	0.00	LAND	P												
Juvenile	YOY_AP020541	0.00	1.00	0.00	1.00	0.00	0.00	0.00	0.00	LAND	P												
Juvenile	YOY_AP020542	0.00	1.00	0.00	1.00	0.00	0.00	0.00	0.00	LAND	P												
Juvenile	YOY_AP020543	0.00	1.00	0.00	1.00	0.00	0.00	0.00	0.00	LAND	P												
Juvenile	YOY_AP020544	0.00	1.00	0.00	1.00	0.00	0.00	0.00	0.00	LAND	P												
Juvenile	YOY_AP020545	0.00	1.00	0.00	1.00	0.00	0.00	0.00	0.00	LAND	P												
Juvenile	YOY_AP020546	0.00	1.00	0.00	1.00	0.00	0.00	0.00	0.00	LAND	P												
Juvenile	YOY_AP020547	0.00	1.00	0.00	1.00	0.00	0.00	0.00	0.00	LAND	P												
Juvenile	YOY_AP020548	0.00	1.00	0.00	1.00	0.00	0.00	0.00	0.00	LAND	P												
Juvenile	YOY_AP020549	0.00	1.00	0.00	1.00	0.00	0.00	0.00	0.00	LAND	P												
Juvenile	YOY_AP020550	0.00	1.00	0.00	1.00	0.00	0.00	0.00	0.00	LAND	P												
Juvenile	YOY_AP020551	0.00	1.00	0.00	1.00	0.00	0.00	0.00	0.00	LAND	P												
Juvenile	YOY_AP020552	0.00	1.00	0.00	1.00	0.00	0.00	0.00	0.00	LAND	P												
Juvenile	YOY_AP020553	0.00	1.00	0.00	1.00	0.00	0.00	0.00	0.00	LAND	P												
Juvenile	YOY_AP020554	0.00	1.00	0.00	1.00	0.00	0.00	0.00	0.00	LAND	P												
Juvenile	YOY_AP020555	0.00	1.00	0.00	1.00	0.00	0.00	0.00	0.00	LAND	P												
Juvenile	YOY_AP020556	0.00	1.00	0.00	1.00	0.00	0.00	0.00	0.00	LAND	P												
Juvenile	YOY_AP020557	0.00	1.00	0.00	1.00	0.00	0.00	0.00	0.00	LAND	P												
Juvenile	YOY_AP020558	0.00	1.00	0.00	1.00	0.00	0.00	0.00	0.00	LAND	P												
Juvenile	YOY_AP020559	0.00	1.00	0.00	1.00	0.00	0.00	0.00	0.00	LAND	P												
Juvenile	YOY_AP020560	0.00	1.00	0.00	1.00	0.00	0.00	0.00	0.00	LAND	P												
Juvenile	YOY_AP020561	0.00	1.00	0.00	1.00	0.00	0.00	0.00	0.00	LAND	P												
Juvenile	YOY_AP020562	0.00	1.00	0.00	1.00	0.00	0.00	0.00	0.00	LAND	P												
Juvenile	YOY_AP020563	0.00	1.00	0.00	1.00	0.00	0.00	0.00	0.00	LAND	P												
Juvenile	YOY_AP020564	0.00	1.00	0.00	1.00	0.00	0.00	0.00	0.00	LAND	P												
Juvenile	YOY_AP020565	0.00	1.00	0.00	1.00	0.00	0.00	0.00	0.00	LAND	P												
Juvenile	YOY_AP020566	0.00	1.00	0.00	1.00	0.00	0.00	0.00	0.00	LAND	P												
Juvenile	YOY_AP020567	0.00	1.00	0.00	1.00	0.00	0.00	0.00	0.00	LAND	P												
Juvenile	YOY_AP020568	0.00	1.00	0.00	1.00	0.00	0.00	0.00	0.00	LAND	P												
Juvenile	YOY_AP020569	0.00	1.00	0.00	1.00	0.00	0.00	0.00	0.00	LAND	P												
Juvenile	YOY_AP020776	1.00	0.00	1.00	0.00	0.00	0.00	0.00	0.00	ANAD	P	BvAP019311	M	4/12/17	260	3.93E+01	1	BvAP019212	F	4/19/17	278	4.98E+01	1
Juvenile	YOY_AP020571	0.00	1.00	0.00	1.00	0.00	0.00	0.00	0.00	LAND	P												
Juvenile	YOY_AP020572	0.00	1.00	0.00	1.00	0.00	0.00	0.00	0.00	LAND	P												
Juvenile	YOY_AP020573	0.00	1.00	0.00	1.00	0.00	0.00	0.00	0.00	LAND	P												
Juvenile	YOY_AP020574	1.00	0.00	1.00	0.00	0.00	0.00	0.00	0.00	ANAD	P	BvAP017401	F	3/30/17	261	3.24E+01	1	BvAP017300	M	3/30/17	268	3.88E+01	1
Juvenile	YOY_AP020575	0.00	1.00	0.00	1.00	0.00	0.00	0.00	0.00	LAND	P												
Juvenile	YOY_AP020576	0.00	1.00	0.00	1.00	0.00	0.00	0.00	0.00	LAND	P												
Juvenile	YOY_AP020577	0.00	1.00	0.00	1.00	0.00	0.00	0.00	0.00	LAND	P												
Juvenile	YOY_AP020578	0.00	1.00	0.00	1.00	0.00	0.00	0.00	0.00	LAND	P												
Juvenile	YOY_AP020579	0.00	1.00	0.00	1.00	0.00	0.00	0.00	0.00	LAND	P												
Juvenile	YOY_AP020580	0.00	1.00	0.00	1.00	0.00	0.00	0.00	0.00	LAND	P												
Juvenile	YOY_AP020581	0.00	1.00	0.00	1.00	0.00	0.00	0.00	0.00	LAND	P												
Juvenile	YOY_AP020582	0.00	1.00	0.00	1.00	0.00	0.00	0.00	0.00	LAND	P												
Juvenile	YOY_AP020583	0.00	1.00	0.00	1.00	0.00	0.00	0.00	0.00	LAND	P												
Juvenile	YOY_AP020584	0.00	1.00	0.00	1.00	0.00	0.00	0.00	0.00	LAND	P												
Juvenile	YOY_AP020585	0.00	1.00	0.00	1.00	0.00	0.00	0.00	0.00	LAND	P												
Juvenile	YOY_AP020586	0.00	1.00	0.00	1.00	0.00	0.00	0.00	0.00	LAND	P												
Juvenile	YOY_AP020587	0.00	1.00	0.00	1.00	0.00	0.00	0.00	0.00	LAND	P												
Juvenile	YOY_AP020588	0.00	1.00	0.00	1.00	0.00	0.00	0.00	0.00	LAND	P												
Juvenile	YOY_AP020589	0.00	1.00	0.00	1.00	0.00	0.00	0.00	0.00	LAND	P												
Juvenile	YOY_AP020590	0.00	1.00	0.00	1.00	0.00	0.00	0.00	0.00	LAND	P												
Juvenile	YOY_AP020591	0.33	0.67	0.00	0.00	0.00	0.24	0.00	0.76	LAND_BC	P												
Juvenile	YOY_AP020592	0.00	1.00	0.00	1.00	0.00	0.00	0.00	0.00	LAND	P												
Juvenile	YOY_AP020593	0.00	1.00	0.00	1.00	0.00	0.00	0.00	0.00	LAND	P												
Juvenile	YOY_AP020594	0.00	1.00	0.00	1.00	0.00	0.00	0.00	0.00	LAND	P												
Juvenile	YOY_AP020595	0.00	1.00	0.00	1.00	0.00	0.00	0.00	0.00	LAND	P												
Juvenile	YOY_AP020596	0.00	1.00	0.00	1.00	0.00	0.00	0.00	0.00	LAND	P												
Juvenile	YOY_AP020597	0.00	1.00	0.00	1.00	0.00	0.00	0.00	0.00	LAND	P												
Juvenile	YOY_AP020598	0.00	1.00	0.00	1.00	0.00	0.00	0.00	0.00	LAND	P												
Juvenile	YOY_AP020599	0.00	1.00	0.00	1.00	0.00	0.00	0.00	0.00	LAND	P												
Juvenile	YOY_AP020600	0.00	1.00	0.00	1.00	0.00	0.00	0.00	0.00	LAND	P												
Juvenile	YOY_AP020601	0.00	1.00	0.00	1.00	0.00	0.00	0.00	0.00	LAND	P												
Juvenile	YOY_AP020602	0.00	1.00	0.00	1.00	0.00	0.00	0.00	0.00	LAND	P												
Juvenile	YOY_AP020603	0.00	1.00	0.00	1.00	0.00	0.00	0.00	0.00	LAND	P												
Juvenile	YOY_AP020604	0.00	1.00	0.00	1.00	0.00	0.00	0.00	0.00	LAND	P												
Juvenile	YOY_AP020605	0.00	1.00	0.00	1.00	0.00	0.00	0.00	0.00	LAND	P												
Juvenile	YOY_AP020606	0.00	1.00	0.00	1.00	0.00	0.00	0.00	0.00	LAND	P												
Juvenile	YOY_AP020607	0.00	1.00	0.00	1.00	0.00	0.00	0.00	0.00	LAND	P												
Juvenile	YOY_AP020608	0.00	1.00	0.00	1.00	0.00	0.00	0.00	0.00	LAND	P												
Juvenile	YOY_AP020609	0.00	1.00	0.00	1.00	0.00	0.00	0.00	0.00	LAND	P												
Juvenile	YOY_AP020610	0.00	1.00	0.00	1.00	0.00	0.00	0.00	0.00	LAND	P												
Juvenile	YOY_AP020611	0.00	1.00	0.00	1.00	0.00	0.00	0.00	0.00	LAND	P												
Juvenile	YOY_AP020612	0.00	1.00	0.00	1.00	0.00	0.00	0.00	0.00	LAND	P												
Juvenile	YOY_AP020613	0.00	1.00	0.00	1.00	0.00	0.00	0.00	0.00	LAND	P												
Juvenile	YOY_AP020614	0.00	1.00	0.00	1.00	0.00	0.00	0.00	0.00	LAND	P												
Juvenile	YOY_AP020615	0.00	1.00	0.00	1.00	0.00	0.00	0.00	0.00	LAND	P												
Juvenile	YOY_AP020616	0.00	1.00	0.00	1.00	0.00	0.00	0.00	0.00	LAND	P												
Juvenile	YOY_AP020617	0.00	1.00	0.00	1.00	0.00	0.00	0.00	0.00	LAND	P												
Juvenile	YOY_AP020618	0.00	1.00	0.00	1.00	0.00	0.00	0.00	0.00	LAND	P												
Juvenile	YOY_AP020619	0.00	1.00	0.00	1.00	0.00	0.00	0.00	0.00	LAND	P												
Juvenile	YOY_AP020620	0.00	1.00	0.00	1.00	0.00	0.00	0.00	0.00	LAND	P												
Juvenile	YOY_AP020621	0.00	1.00	0.00	1.00	0.00	0.00	0.00	0.00	LAND	P												
Juvenile	YOY_AP020622	0.00	1.00	0.00	1.00	0.00	0.00	0.00	0.00	LAND	P												
Juvenile	YOY_AP020623	1.00	0.00	1.00	0.00	0.00	0.00	0.00	0.00	ANAD	P	BvAP018986	M	4/12/17	254	5.29E+01	1	?	?	?	?	?	?
Juvenile	YOY_AP020624	0.00	1.00	0.00	1.00	0.00	0.00	0.00	0.00	LAND	P												

Juvenile	YOY_AP020625	0.00	1.00	0.00	1.00	0.00	0.00	0.00	0.00	LAND	P												
Juvenile	YOY_AP020626	0.00	1.00	0.00	1.00	0.00	0.00	0.00	0.00	LAND	P												
Juvenile	YOY_AP020627	0.00	1.00	0.00	1.00	0.00	0.00	0.00	0.00	LAND	P												
Juvenile	YOY_AP020628	1.00	0.00	1.00	0.00	0.00	0.00	0.00	0.00	ANAD	P	BvAP018457	M	4/19/17	262	4.91E+01	1	BvAP017579	F	3/30/17	280	3.82E+01	1
Juvenile	YOY_AP020629	0.00	1.00	0.00	1.00	0.00	0.00	0.00	0.00	LAND	P												
Juvenile	YOY_AP020630	0.00	1.00	0.00	1.00	0.00	0.00	0.00	0.00	LAND	P												
Juvenile	YOY_AP020631	0.00	1.00	0.00	1.00	0.00	0.00	0.00	0.00	LAND	P												
Juvenile	YOY_AP020835	0.99	0.01	1.00	0.00	0.00	0.00	0.00	0.00	ANAD	P	BvAP019256	F	4/12/17	282	4.01E+01	1	BvAP019017	M	4/12/17	255	3.01E+01	1
Juvenile	YOY_AP020634	0.00	1.00	0.00	1.00	0.00	0.00	0.00	0.00	LAND	P												
Juvenile	YOY_AP020635	0.27	0.73	0.00	0.00	0.00	0.00	0.00	1.00	LAND_BC	P												
Juvenile	YOY_AP020636	0.00	1.00	0.00	1.00	0.00	0.00	0.00	0.00	LAND	P												
Juvenile	YOY_AP020637	0.00	1.00	0.00	1.00	0.00	0.00	0.00	0.00	LAND	P												
Juvenile	YOY_AP020638	0.00	1.00	0.00	1.00	0.00	0.00	0.00	0.00	LAND	P												
Juvenile	YOY_AP020639	0.00	1.00	0.00	1.00	0.00	0.00	0.00	0.00	LAND	P												
Juvenile	YOY_AP020640	0.00	1.00	0.00	1.00	0.00	0.00	0.00	0.00	LAND	P												
Juvenile	YOY_AP020641	0.00	1.00	0.00	1.00	0.00	0.00	0.00	0.00	LAND	P												
Juvenile	YOY_AP020642	0.00	1.00	0.00	1.00	0.00	0.00	0.00	0.00	LAND	P												
Juvenile	YOY_AP020890	1.00	0.00	1.00	0.00	0.00	0.00	0.00	0.00	ANAD	P	BvAP019264	M	4/12/17	265	4.70E+01	1	BvAP019010	F	4/12/17	253	4.86E+01	1
Juvenile	YOY_AP020644	0.00	1.00	0.00	1.00	0.00	0.00	0.00	0.00	LAND	P												
Juvenile	YOY_AP020645	0.00	1.00	0.00	1.00	0.00	0.00	0.00	0.00	LAND	P												
Juvenile	YOY_AP020646	0.00	1.00	0.00	1.00	0.00	0.00	0.00	0.00	LAND	P												
Juvenile	YOY_AP020647	0.00	1.00	0.00	1.00	0.00	0.00	0.00	0.00	LAND	P												
Juvenile	YOY_AP020648	0.00	1.00	0.00	1.00	0.00	0.00	0.00	0.00	LAND	P												
Juvenile	YOY_AP020649	0.00	1.00	0.00	1.00	0.00	0.00	0.00	0.00	LAND	P												
Juvenile	YOY_AP020650	0.00	1.00	0.00	1.00	0.00	0.00	0.00	0.00	LAND	P												
Juvenile	YOY_AP020651	0.00	1.00	0.00	1.00	0.00	0.00	0.00	0.00	LAND	P												
Juvenile	YOY_AP020652	0.00	1.00	0.00	1.00	0.00	0.00	0.00	0.00	LAND	P												
Juvenile	YOY_AP020653	0.29	0.71	0.00	0.00	0.00	0.00	0.00	1.00	LAND_BC	P												
Juvenile	YOY_AP020654	0.00	1.00	0.00	1.00	0.00	0.00	0.00	0.00	LAND	P												
Juvenile	YOY_AP020655	0.00	1.00	0.00	1.00	0.00	0.00	0.00	0.00	LAND	P												
Juvenile	YOY_AP020656	0.00	1.00	0.00	1.00	0.00	0.00	0.00	0.00	LAND	P												
Juvenile	YOY_AP020657	0.00	1.00	0.00	1.00	0.00	0.00	0.00	0.00	LAND	P												
Juvenile	YOY_AP020658	0.00	1.00	0.00	1.00	0.00	0.00	0.00	0.00	LAND	P												
Juvenile	YOY_AP020659	0.00	1.00	0.00	1.00	0.00	0.00	0.00	0.00	LAND	P												
Juvenile	YOY_AP020660	0.00	1.00	0.00	1.00	0.00	0.00	0.00	0.00	LAND	P												
Juvenile	YOY_AP020661	0.00	1.00	0.00	1.00	0.00	0.00	0.00	0.00	LAND	P												
Juvenile	YOY_AP020662	0.00	1.00	0.00	1.00	0.00	0.00	0.00	0.00	LAND	P												
Juvenile	YOY_AP020663	0.00	1.00	0.00	1.00	0.00	0.00	0.00	0.00	LAND	P												
Juvenile	YOY_AP020664	0.00	1.00	0.00	1.00	0.00	0.00	0.00	0.00	LAND	P												
Juvenile	YOY_AP020665	0.00	1.00	0.00	1.00	0.00	0.00	0.00	0.00	LAND	P												
Juvenile	YOY_AP020666	0.00	1.00	0.00	1.00	0.00	0.00	0.00	0.00	LAND	P												
Juvenile	YOY_AP020667	0.00	1.00	0.00	1.00	0.00	0.00	0.00	0.00	LAND	P												
Juvenile	YOY_AP020668	0.00	1.00	0.00	1.00	0.00	0.00	0.00	0.00	LAND	P												
Juvenile	YOY_AP020669	0.00	1.00	0.00	1.00	0.00	0.00	0.00	0.00	LAND	P												
Juvenile	YOY_AP020670	0.00	1.00	0.00	1.00	0.00	0.00	0.00	0.00	LAND	P												
Juvenile	YOY_AP020671	0.00	1.00	0.00	1.00	0.00	0.00	0.00	0.00	LAND	P												
Juvenile	YOY_AP020672	0.00	1.00	0.00	1.00	0.00	0.00	0.00	0.00	LAND	P												
Juvenile	YOY_AP020673	0.00	1.00	0.00	1.00	0.00	0.00	0.00	0.00	LAND	P												
Juvenile	YOY_AP020674	1.00	0.00	1.00	0.00	0.00	0.00	0.00	0.00	ANAD	P	BvAP018739	F	4/12/17	264	5.54E+01	1	BvAP016789	M	3/30/17	252	3.52E+01	1
Juvenile	YOY_AP020899	1.00	0.00	1.00	0.00	0.00	0.00	0.00	0.00	ANAD	P	BvAP019060	F	4/12/17	269	3.48E+01	1	BvAP018629	M	4/12/17	245	3.52E+01	1
Juvenile	YOY_AP020676	0.00	1.00	0.00	1.00	0.00	0.00	0.00	0.00	LAND	P												
Juvenile	YOY_AP020677	0.00	1.00	0.00	1.00	0.00	0.00	0.00	0.00	LAND	P												
Juvenile	YOY_AP020678	0.00	1.00	0.00	1.00	0.00	0.00	0.00	0.00	LAND	P												
Juvenile	YOY_AP020679	0.00	1.00	0.00	1.00	0.00	0.00	0.00	0.00	LAND	P												
Juvenile	YOY_AP020680	0.00	1.00	0.00	1.00	0.00	0.00	0.00	0.00	LAND	P												
Juvenile	YOY_AP020681	0.00	1.00	0.00	1.00	0.00	0.00	0.00	0.00	LAND	P												
Juvenile	YOY_AP020682	0.00	1.00	0.00	1.00	0.00	0.00	0.00	0.00	LAND	P												
Juvenile	YOY_AP020683	0.00	1.00	0.00	1.00	0.00	0.00	0.00	0.00	LAND	P												
Juvenile	YOY_AP020684	0.00	1.00	0.00	1.00	0.00	0.00	0.00	0.00	LAND	P												
Juvenile	YOY_AP020685	1.00	0.00	1.00	0.00	0.00	0.00	0.00	0.00	ANAD	P	BvAP019240	F	4/12/17	282	4.87E+01	1	BvAP017154	M	3/30/17	267	3.73E+01	1
Juvenile	YOY_AP020686	0.00	1.00	0.00	1.00	0.00	0.00	0.00	0.00	LAND	P												
Juvenile	YOY_AP019912	1.00	0.00	1.00	0.00	0.00	0.00	0.00	0.00	ANAD	P	BvAP019156	M	4/19/17	266	5.21E+01	1	BvAP017935	M	4/12/17	260	3.75E+01	1
Juvenile	YOY_AP020688	0.00	1.00	0.00	1.00	0.00	0.00	0.00	0.00	LAND	P												
Juvenile	YOY_AP020689	0.00	1.00	0.00	1.00	0.00	0.00	0.00	0.00	LAND	P												
Juvenile	YOY_AP020690	0.00	1.00	0.00	1.00	0.00	0.00	0.00	0.00	LAND	P												
Juvenile	YOY_AP020691	0.00	1.00	0.00	1.00	0.00	0.00	0.00	0.00	LAND	P												
Juvenile	YOY_AP020692	0.00	1.00	0.00	1.00	0.00	0.00	0.00	0.00	LAND	P												
Juvenile	YOY_AP020693	0.00	1.00	0.00	1.00	0.00	0.00	0.00	0.00	LAND	P												
Juvenile	YOY_AP020694	0.00	1.00	0.00	1.00	0.00	0.00	0.00	0.00	LAND	P												
Juvenile	YOY_AP019954	1.00	0.00	1.00	0.00	0.00	0.00	0.00	0.00	ANAD	L	BvAP018374	F	4/19/17	253	3.79E+01	1	BvAP017248	M	4/12/17	270	3.65E+01	1
Juvenile	YOY_AP020696	0.00	1.00	0.00	1.00	0.00	0.00	0.00	0.00	LAND	P												
Juvenile	YOY_AP019972	1.00	0.00	1.00	0.00	0.00	0.00	0.00	0.00	ANAD	L	BvAP018411	M	4/19/17	265	3.46E+01	1	BvAP017743	F	4/12/17	262	5.62E+01	1
Juvenile	YOY_AP019981	1.00	0.00	1.00	0.00	0.00	0.00	0.00	0.00	ANAD	L	BvAP018531	F	4/19/17	279	4.28E+01	1	BvAP018327	M	4/19/17	260	2.72E+01	1
Juvenile	YOY_AP020699	0.00	1.00	0.00	1.00	0.00	0.00	0.00	0.00	LAND	P												
Juvenile	YOY_AP020700	0.00	1.00	0.00	1.00	0.00	0.00	0.00	0.00	LAND	P												
Juvenile	YOY_AP020701	0.00	1.00	0.00	1.00	0.00	0.00	0.00	0.00	LAND	P												
Juvenile	YOY_AP020702	0.00	1.00	0.00	1.00	0.00	0.00	0.00	0.00	LAND	P												
Juvenile	YOY_AP020703	0.00	1.00	0.00	1.00	0.00	0.00	0.00	0.00	LAND	P												
Juvenile	YOY_AP020704	0.00	1.00	0.00	1.00	0.00	0.00	0.00	0.00	LAND	P												
Juvenile	YOY_AP020705	0.00	1.00	0.00	1.00	0.00	0.00	0.00	0.00	LAND	P												
Juvenile	YOY_AP020706	0.00	1.00	0.00	1.00	0.00	0.00	0.00	0.00	LAND	P												
Juvenile	YOY_AP020707	0.00	1.00	0.00	1.00	0.00	0.00	0.00	0.00	LAND	P												
Juvenile	YOY_AP020708	0.00	1.00	0.00	1.00	0.00	0.00	0.00	0.00	LAND	P												
Juvenile	YOY_AP019999	1.00	0.00	1.00	0.00	0.00	0.00	0.00	0.00	ANAD	P	BvAP019207	F	4/19/17	306	4.21E+01	1	BvAP017999	M	4/12/17	280	3.56E+01	1
Juvenile	YOY_AP020710	0.00	1.00	0.00	1.00	0.00	0.00	0.00	0.00	LAND	P												
Juvenile	YOY_AP020711	0.00	1.00	0.00	1.00	0.00	0.00	0.00	0.00	LAND	P												
Juvenile	YOY_AP020712	0.00	1.00	0.00	1.00	0.00	0.00	0.00	0.00	LAND	P												
Juvenile	YOY_AP020713	0.00	1.00	0.00	1.00	0.00	0.00	0.00	0.00	LAND	P												
Juvenile	YOY_AP020714	0.00	1.00	0.00	1.00	0.00	0.00	0.00	0.00	LAND	P												
Juvenile	YOY_AP020715	0.00	1.00	0.00	1.00	0.00	0.00	0.00	0.00	LAND	P												
Juvenile	YOY_AP020716	0.00	1.00	0.00	1.00	0.00	0.00	0.00	0.00	LAND	P												
Juvenile	YOY_AP020717	0.00	1.00	0.00	1.00	0.00	0.00	0.00	0.00	LAND	P												
Juvenile	YOY_AP020718	0.00	1.00	0.00	1.00	0.00	0.00	0.00	0.00	LAND	P												
Juvenile	YOY_AP020719	0.00	1.00	0.00	1.00	0.00	0.00	0.00	0.00	LAND	P												
Juvenile	YOY_AP020720	0.00	1.00	0.00	1.00	0.00	0.00	0.00	0.00	LAND	P												
Juvenile	YOY_AP020721	0.00	1.00	0.00	1.00	0.00	0.00	0.00	0.00	LAND	P												
Juvenile	YOY_AP020722	0.00	1.00	0.00	1.00	0.00	0.00	0.00	0.00	LAND	P												
Juvenile	YOY_AP020723	0.00	1.00	0.00	1.00	0.00	0.00	0.00	0.00	LAND	P												
Juvenile	YOY_AP020724	0.00	1.00	0.00	1.00	0.00	0.00	0.00	0.00	LAND	P												
Juvenile	YOY_AP020725	0.00	1.00	0.00	1.00	0.00	0.00	0.00	0.00	LAND	P												
Juvenile	YOY_AP020726	0.00	1.00	0.00	1.00	0.00	0.00	0.00	0.00	LAND	P												

Juvenile	YOY_AP020727	0.00	1.00	0.00	1.00	0.00	0.00	0.00	0.00	LAND	P												
Juvenile	YOY_AP020728	0.00	1.00	0.00	1.00	0.00	0.00	0.00	0.00	LAND	P												
Juvenile	YOY_AP020729	0.00	1.00	0.00	1.00	0.00	0.00	0.00	0.00	LAND	P												
Juvenile	YOY_AP020730	0.00	1.00	0.00	1.00	0.00	0.00	0.00	0.00	LAND	P												
Juvenile	YOY_AP020731	0.00	1.00	0.00	1.00	0.00	0.00	0.00	0.00	LAND	P												
Juvenile	YOY_AP020732	0.00	1.00	0.00	1.00	0.00	0.00	0.00	0.00	LAND	P												
Juvenile	YOY_AP020733	0.00	1.00	0.00	1.00	0.00	0.00	0.00	0.00	LAND	P												
Juvenile	YOY_AP020734	0.00	1.00	0.00	1.00	0.00	0.00	0.00	0.00	LAND	P												
Juvenile	YOY_AP020735	0.00	1.00	0.00	1.00	0.00	0.00	0.00	0.00	LAND	P												
Juvenile	YOY_AP020736	0.00	1.00	0.00	1.00	0.00	0.00	0.00	0.00	LAND	P												
Juvenile	YOY_AP020737	0.00	1.00	0.00	1.00	0.00	0.00	0.00	0.00	LAND	P												
Juvenile	YOY_AP020260	0.99	0.01	1.00	0.00	0.00	0.00	0.00	0.00	ANAD	P	BvAP019377	M	4/19/17	248	2.99E+01	1	BvAP019253	F	4/12/17	282	4.10E+01	1
Juvenile	YOY_AP020739	0.00	1.00	0.00	1.00	0.00	0.00	0.00	0.00	LAND	P												
Juvenile	YOY_AP020740	0.00	1.00	0.00	1.00	0.00	0.00	0.00	0.00	LAND	P												
Juvenile	YOY_AP020741	0.00	1.00	0.00	1.00	0.00	0.00	0.00	0.00	LAND	P												
Juvenile	YOY_AP020742	0.00	1.00	0.00	1.00	0.00	0.00	0.00	0.00	LAND	P												
Juvenile	YOY_AP020743	0.00	1.00	0.00	1.00	0.00	0.00	0.00	0.00	LAND	P												
Juvenile	YOY_AP020286	1.00	0.00	1.00	0.00	0.00	0.00	0.00	0.00	ANAD	P	BvAP018246	M	4/19/17	257	5.02E+01	1	BvAP018148	F	4/19/17	255	6.11E+01	1
Juvenile	YOY_AP020745	0.00	1.00	0.00	1.00	0.00	0.00	0.00	0.00	LAND	P												
Juvenile	YOY_AP020746	0.00	1.00	0.00	1.00	0.00	0.00	0.00	0.00	LAND	P												
Juvenile	YOY_AP020747	0.00	1.00	0.00	1.00	0.00	0.00	0.00	0.00	LAND	P												
Juvenile	YOY_AP020748	0.00	1.00	0.00	1.00	0.00	0.00	0.00	0.00	LAND	P												
Juvenile	YOY_AP020749	0.00	1.00	0.00	1.00	0.00	0.00	0.00	0.00	LAND	P												
Juvenile	YOY_AP020750	0.00	1.00	0.00	1.00	0.00	0.00	0.00	0.00	LAND	P												
Juvenile	YOY_AP020751	0.00	1.00	0.00	1.00	0.00	0.00	0.00	0.00	LAND	P												
Juvenile	YOY_AP020752	0.00	1.00	0.00	1.00	0.00	0.00	0.00	0.00	LAND	P												
Juvenile	YOY_AP020753	0.00	1.00	0.00	1.00	0.00	0.00	0.00	0.00	LAND	P												
Juvenile	YOY_AP020754	0.00	1.00	0.00	1.00	0.00	0.00	0.00	0.00	LAND	P												
Juvenile	YOY_AP020381	1.00	0.00	1.00	0.00	0.00	0.00	0.00	0.00	ANAD	P	BvAP019206	F	4/19/17	257	4.28E+01	1	BvAP018798	M	4/12/17	269	3.88E+01	1
Juvenile	YOY_AP020643	1.00	0.00	1.00	0.00	0.00	0.00	0.00	0.00	ANAD	P	BvAP019663	M	4/19/17	276	3.99E+01	1	BvAP017926	F	4/12/17	283	3.65E+01	1
Juvenile	YOY_AP020757	0.00	1.00	0.00	1.00	0.00	0.00	0.00	0.00	LAND	P												
Juvenile	YOY_AP020758	0.00	1.00	0.00	1.00	0.00	0.00	0.00	0.00	LAND	P												
Juvenile	YOY_AP020759	0.00	1.00	0.00	1.00	0.00	0.00	0.00	0.00	LAND	P												
Juvenile	YOY_AP020760	0.00	1.00	0.00	1.00	0.00	0.00	0.00	0.00	LAND	P												
Juvenile	YOY_AP020761	0.00	1.00	0.00	1.00	0.00	0.00	0.00	0.00	LAND	P												
Juvenile	YOY_AP020762	0.00	1.00	0.00	1.00	0.00	0.00	0.00	0.00	LAND	P												
Juvenile	YOY_AP020763	0.00	1.00	0.00	1.00	0.00	0.00	0.00	0.00	LAND	P												
Juvenile	YOY_AP020261	0.45	0.55	0.00	0.00	0.00	1.00	0.00	0.00	F1	P	BvAP019209	M	4/19/17	253	2.08E+01	1						
Juvenile	YOY_AP020765	0.00	1.00	0.00	1.00	0.00	0.00	0.00	0.00	LAND	P												
Juvenile	YOY_AP020766	0.00	1.00	0.00	1.00	0.00	0.00	0.00	0.00	LAND	P												
Juvenile	YOY_AP020767	0.00	1.00	0.00	1.00	0.00	0.00	0.00	0.00	LAND	P												
Juvenile	YOY_AP020768	0.00	1.00	0.00	1.00	0.00	0.00	0.00	0.00	LAND	P												
Juvenile	YOY_AP020769	0.00	1.00	0.00	1.00	0.00	0.00	0.00	0.00	LAND	P												
Juvenile	YOY_AP020687	1.00	0.00	1.00	0.00	0.00	0.00	0.00	0.00	ANAD	P	BvAP018432	F	4/19/17	259	4.04E+01	1	BvAP018001	M	4/12/17	259	4.37E+01	1
Juvenile	YOY_AP020771	0.00	1.00	0.00	1.00	0.00	0.00	0.00	0.00	LAND	P												
Juvenile	YOY_AP020772	0.00	1.00	0.00	1.00	0.00	0.00	0.00	0.00	LAND	P												
Juvenile	YOY_AP020773	0.00	1.00	0.00	1.00	0.00	0.00	0.00	0.00	LAND	P												
Juvenile	YOY_AP020774	0.00	1.00	0.00	1.00	0.00	0.00	0.00	0.00	LAND	P												
Juvenile	YOY_AP020775	0.00	1.00	0.00	1.00	0.00	0.00	0.00	0.00	LAND	P												
Juvenile	YOY_AP020695	0.99	0.01	1.00	0.00	0.00	0.00	0.00	0.00	ANAD	P	BvAP019206	F	4/19/17	257	2.57E+01	1	BvAP018634	M	4/12/17	283	4.49E+01	1
Juvenile	YOY_AP020777	0.00	1.00	0.00	1.00	0.00	0.00	0.00	0.00	LAND	P												
Juvenile	YOY_AP020778	0.00	1.00	0.00	1.00	0.00	0.00	0.00	0.00	LAND	P												
Juvenile	YOY_AP020779	0.00	1.00	0.00	1.00	0.00	0.00	0.00	0.00	LAND	P												
Juvenile	YOY_AP020780	0.00	1.00	0.00	1.00	0.00	0.00	0.00	0.00	LAND	P												
Juvenile	YOY_AP020781	0.00	1.00	0.00	1.00	0.00	0.00	0.00	0.00	LAND	P												
Juvenile	YOY_AP020782	0.00	1.00	0.00	1.00	0.00	0.00	0.00	0.00	LAND	P												
Juvenile	YOY_AP020783	0.00	1.00	0.00	1.00	0.00	0.00	0.00	0.00	LAND	P												
Juvenile	YOY_AP020784	0.00	1.00	0.00	1.00	0.00	0.00	0.00	0.00	LAND	P												
Juvenile	YOY_AP020785	0.00	1.00	0.00	1.00	0.00	0.00	0.00	0.00	LAND	P												
Juvenile	YOY_AP020786	0.00	1.00	0.00	1.00	0.00	0.00	0.00	0.00	LAND	P												
Juvenile	YOY_AP020787	0.00	1.00	0.00	1.00	0.00	0.00	0.00	0.00	LAND	P												
Juvenile	YOY_AP020788	0.00	1.00	0.00	1.00	0.00	0.00	0.00	0.00	LAND	P												
Juvenile	YOY_AP020698	1.00	0.00	1.00	0.00	0.00	0.00	0.00	0.00	ANAD	P	BvAP019209	M	4/19/17	253	3.84E+01	1	BvAP018399	M	4/19/17	266	4.26E+01	1
Juvenile	YOY_AP020790	0.00	1.00	0.00	1.00	0.00	0.00	0.00	0.00	LAND	P												
Juvenile	YOY_AP020791	0.00	1.00	0.00	1.00	0.00	0.00	0.00	0.00	LAND	P												
Juvenile	YOY_AP020792	0.00	1.00	0.00	1.00	0.00	0.00	0.00	0.00	LAND	P												
Juvenile	YOY_AP020793	0.00	1.00	0.00	1.00	0.00	0.00	0.00	0.00	LAND	P												
Juvenile	YOY_AP020794	0.00	1.00	0.00	1.00	0.00	0.00	0.00	0.00	LAND	P												
Juvenile	YOY_AP020795	0.00	1.00	0.00	1.00	0.00	0.00	0.00	0.00	LAND	P												
Juvenile	YOY_AP020796	0.00	1.00	0.00	1.00	0.00	0.00	0.00	0.00	LAND	P												
Juvenile	YOY_AP020797	0.00	1.00	0.00	1.00	0.00	0.00	0.00	0.00	LAND	P												
Juvenile	YOY_AP020798	0.00	1.00	0.00	1.00	0.00	0.00	0.00	0.00	LAND	P												
Juvenile	YOY_AP020799	0.00	1.00	0.00	1.00	0.00	0.00	0.00	0.00	LAND	P												
Juvenile	YOY_AP020800	0.00	1.00	0.00	1.00	0.00	0.00	0.00	0.00	LAND	P												
Juvenile	YOY_AP020801	0.00	1.00	0.00	1.00	0.00	0.00	0.00	0.00	LAND	P												
Juvenile	YOY_AP020802	0.00	1.00	0.00	1.00	0.00	0.00	0.00	0.00	LAND	P												
Juvenile	YOY_AP020803	0.00	1.00	0.00	1.00	0.00	0.00	0.00	0.00	LAND	P												
Juvenile	YOY_AP020804	0.00	1.00	0.00	1.00	0.00	0.00	0.00	0.00	LAND	P												
Juvenile	YOY_AP020805	0.00	1.00	0.00	1.00	0.00	0.00	0.00	0.00	LAND	P												
Juvenile	YOY_AP020806	0.21	0.79	0.00	0.00	0.00	0.00	0.00	1.00	LAND_BC	P												
Juvenile	YOY_AP020298	0.47	0.53	0.00	0.00	0.85	0.15	0.00	0.00	F1	P	BvAP019209	M	4/19/17	253	1.84E+01	1						
Juvenile	YOY_AP020808	0.00	1.00	0.00	1.00	0.00	0.00	0.00	0.00	LAND	P												
Juvenile	YOY_AP020809	0.00	1.00	0.00	1.00	0.00	0.00	0.00	0.00	LAND	P												
Juvenile	YOY_AP020810	0.00	1.00	0.00	1.00	0.00	0.00	0.00	0.00	LAND	P												
Juvenile	YOY_AP020811	0.00	1.00	0.00	1.00	0.00	0.00	0.00	0.00	LAND	P												
Juvenile	YOY_AP020812	0.00	1.00	0.00	1.00	0.00	0.00	0.00	0.00	LAND	P												
Juvenile	YOY_AP020813	0.00	1.00	0.00	1.00	0.00	0.00	0.00	0.00	LAND	P												
Juvenile	YOY_AP020814	0.00	1.00	0.00	1.00	0.00	0.00	0.00	0.00	LAND	P												
Juvenile	YOY_AP020815	0.00	1.00	0.00	1.00	0.00	0.00	0.00	0.00	LAND	P												
Juvenile	YOY_AP020816	0.26	0.74	0.00	0.00	0.00	0.00	0.00	1.00	LAND_BC	P												
Juvenile	YOY_AP020817	0.71	0.29	0.00	0.00	0.00	0.33	0.67	0.00	ANAD_BC	P	BvAP018570	F	4/12/17	272	3.17E+01	1						
Juvenile	YOY_AP020818	0.00	1.00	0.00	1.00	0.00	0.00	0.00	0.00	LAND	P												
Juvenile	YOY_AP020820	0.00	1.00	0.00	1.00	0.00	0.00	0.00	0.00	LAND	P												
Juvenile	YOY_AP020821	0.00	1.00	0.00	1.00	0.00	0.00	0.00	0.00	LAND	P												
Juvenile	YOY_AP020822	0.00	1.00	0.00	1.00	0.00	0.00	0.00	0.00	LAND	P												
Juvenile	YOY_AP020823	0.00	1.00	0.00	1.00	0.00	0.00	0.00	0.00	LAND	P												
Juvenile	YOY_AP020825	1.00	0.00	1.00	0.00	0.00	0.00	0.00	0.00	ANAD	P	BvAP018635	F	4/12/17	284	2.69E+01	1	BvAP017386	M	3/30/17	267	5.40E+01	1
Juvenile	YOY_AP020826	0.00	1.00	0.00	1.00	0.00	0.00	0.00	0.00	LAND	P												
Juvenile	YOY_AP020827	0.00	1.00	0.00	1.00	0.00	0.00	0.00	0.00	LAND	P												
Juvenile	YOY_AP020828	0.00	1.00	0.00	1.00	0.00	0.00	0.00	0.00	LAND	P												
Juvenile	YOY_AP020829	0.00	1.00	0.00	1.00	0.00	0.00	0.00	0.00	LAND	P												

Juvenile	YOY_AP020830	0.51	0.49	0.00	0.00	1.00	0.00	0.00	0.00	F1	P	BvAP019214	F	4/19/17	280	2.56E+01	1						
Juvenile	YOY_AP020831	0.99	0.01	1.00	0.00	0.00	0.00	0.00	0.00	ANAD	P	BvAP018312	M	4/19/17	255	5.74E+01	1	BvAP017488	F	3/30/17	280	2.99E+01	1
Juvenile	YOY_AP020832	0.00	1.00	0.00	1.00	0.00	0.00	0.00	0.00	LAND	P												
Juvenile	YOY_AP020833	0.78	0.23	0.00	0.00	0.00	0.00	1.00	0.00	ANAD_BC	P	BvAP018052	F	4/19/17	286	5.17E+01	1						
Juvenile	YOY_AP020834	0.00	1.00	0.00	1.00	0.00	0.00	0.00	0.00	LAND	P												
Juvenile	YOY_AP020738	1.00	0.00	1.00	0.00	0.00	0.00	0.00	0.00	ANAD	P	BvAP019338	M	4/19/17	252	4.55E+01	1	BvAP019062	F	4/12/17	266	2.44E+01	1
Juvenile	YOY_AP020836	0.00	1.00	0.00	1.00	0.00	0.00	0.00	0.00	LAND	P												
Juvenile	YOY_AP020837	1.00	0.00	1.00	0.00	0.00	0.00	0.00	0.00	ANAD	P	BvAP017462	M	3/30/17	274	4.58E+01	1	BvAP016945	F	3/30/17	280	5.33E+01	1
Juvenile	YOY_AP020838	0.00	1.00	0.00	1.00	0.00	0.00	0.00	0.00	LAND	P												
Juvenile	YOY_AP020744	1.00	0.00	1.00	0.00	0.00	0.00	0.00	0.00	ANAD	P	BvAP019382	M	4/19/17	243	2.80E+01	1	BvAP018432	F	4/19/17	259	3.75E+01	1
Juvenile	YOY_AP020840	0.28	0.73	0.00	0.00	0.00	0.00	0.00	1.00	LAND_BC	P												
Juvenile	YOY_AP020841	1.00	0.00	1.00	0.00	0.00	0.00	0.00	0.00	ANAD	P	BvAP017575	M	3/30/17	278	3.04E+01	1	BvAP017147	F	3/30/17	266	3.05E+01	1
Juvenile	YOY_AP020842	0.00	1.00	0.00	1.00	0.00	0.00	0.00	0.00	LAND	P												
Juvenile	YOY_AP020843	0.00	1.00	0.00	1.00	0.00	0.00	0.00	0.00	LAND	P												
Juvenile	YOY_AP020845	0.00	1.00	0.00	1.00	0.00	0.00	0.00	0.00	LAND	P												
Juvenile	YOY_AP020846	0.00	1.00	0.00	1.00	0.00	0.00	0.00	0.00	LAND	P												
Juvenile	YOY_AP020847	0.00	1.00	0.00	1.00	0.00	0.00	0.00	0.00	LAND	P												
Juvenile	YOY_AP020848	0.00	1.00	0.00	1.00	0.00	0.00	0.00	0.00	LAND	P												
Juvenile	YOY_AP020849	0.00	1.00	0.00	1.00	0.00	0.00	0.00	0.00	LAND	P												
Juvenile	YOY_AP020850	0.00	1.00	0.00	1.00	0.00	0.00	0.00	0.00	LAND	P												
Juvenile	YOY_AP020851	0.00	1.00	0.00	1.00	0.00	0.00	0.00	0.00	LAND	P												
Juvenile	YOY_AP020852	0.00	1.00	0.00	1.00	0.00	0.00	0.00	0.00	LAND	P												
Juvenile	YOY_AP020853	1.00	0.00	1.00	0.00	0.00	0.00	0.00	0.00	ANAD	P	BvAP017093	M	3/30/17	246	3.69E+01	1	BvAP017033	F	3/30/17	273	4.77E+01	1
Juvenile	YOY_AP020854	0.00	1.00	0.00	1.00	0.00	0.00	0.00	0.00	LAND	P												
Juvenile	YOY_AP020855	0.00	1.00	0.00	1.00	0.00	0.00	0.00	0.00	LAND	P												
Juvenile	YOY_AP020856	0.00	1.00	0.00	1.00	0.00	0.00	0.00	0.00	LAND	P												
Juvenile	YOY_AP020857	0.00	1.00	0.00	1.00	0.00	0.00	0.00	0.00	LAND	P												
Juvenile	YOY_AP020858	0.00	1.00	0.00	1.00	0.00	0.00	0.00	0.00	LAND	P												
Juvenile	YOY_AP020859	0.00	1.00	0.00	1.00	0.00	0.00	0.00	0.00	LAND	P												
Juvenile	YOY_AP020860	0.00	1.00	0.00	1.00	0.00	0.00	0.00	0.00	LAND	P												
Juvenile	YOY_AP020861	0.00	1.00	0.00	1.00	0.00	0.00	0.00	0.00	LAND	P												
Juvenile	YOY_AP020862	0.00	1.00	0.00	1.00	0.00	0.00	0.00	0.00	LAND	P												
Juvenile	YOY_AP020863	0.00	1.00	0.00	1.00	0.00	0.00	0.00	0.00	LAND	P												
Juvenile	YOY_AP020864	0.00	1.00	0.00	1.00	0.00	0.00	0.00	0.00	LAND	P												
Juvenile	YOY_AP020865	0.00	1.00	0.00	1.00	0.00	0.00	0.00	0.00	LAND	P												
Juvenile	YOY_AP020866	0.55	0.45	0.00	0.00	1.00	0.00	0.00	0.00	F1	P	BvAP019212	F	4/19/17	278	3.21E+01	1						
Juvenile	YOY_AP020867	0.00	1.00	0.00	1.00	0.00	0.00	0.00	0.00	LAND	P												
Juvenile	YOY_AP020440	0.49	0.51	0.00	0.00	0.00	0.99	0.00	0.00	F1	P												
Juvenile	YOY_AP020869	0.00	1.00	0.00	1.00	0.00	0.00	0.00	0.00	LAND	P												
Juvenile	YOY_AP020870	0.00	1.00	0.00	1.00	0.00	0.00	0.00	0.00	LAND	P												
Juvenile	YOY_AP020871	0.00	1.00	0.00	1.00	0.00	0.00	0.00	0.00	LAND	P												
Juvenile	YOY_AP020872	0.00	1.00	0.00	1.00	0.00	0.00	0.00	0.00	LAND	P												
Juvenile	YOY_AP020873	0.00	1.00	0.00	1.00	0.00	0.00	0.00	0.00	LAND	P												
Juvenile	YOY_AP020874	0.00	1.00	0.00	1.00	0.00	0.00	0.00	0.00	LAND	P												
Juvenile	YOY_AP020789	1.00	0.00	1.00	0.00	0.00	0.00	0.00	0.00	ANAD	P	BvAP019361	M	4/19/17	260	4.29E+01	1	BvAP019141	F	4/12/17	270	3.79E+01	1
Juvenile	YOY_AP020876	0.00	1.00	0.00	1.00	0.00	0.00	0.00	0.00	LAND	P												
Juvenile	YOY_AP020877	0.00	1.00	0.00	1.00	0.00	0.00	0.00	0.00	LAND	P												
Juvenile	YOY_AP020878	0.00	1.00	0.00	1.00	0.00	0.00	0.00	0.00	LAND	P												
Juvenile	YOY_AP020879	0.00	1.00	0.00	1.00	0.00	0.00	0.00	0.00	LAND	P												
Juvenile	YOY_AP020880	0.00	1.00	0.00	1.00	0.00	0.00	0.00	0.00	LAND	P												
Juvenile	YOY_AP020881	0.00	1.00	0.00	1.00	0.00	0.00	0.00	0.00	LAND	P												
Juvenile	YOY_AP020882	0.00	1.00	0.00	1.00	0.00	0.00	0.00	0.00	LAND	P												
Juvenile	YOY_AP020883	0.00	1.00	0.00	1.00	0.00	0.00	0.00	0.00	LAND	P												
Juvenile	YOY_AP020884	0.00	1.00	0.00	1.00	0.00	0.00	0.00	0.00	LAND	P												
Juvenile	YOY_AP020885	0.00	1.00	0.00	1.00	0.00	0.00	0.00	0.00	LAND	P												
Juvenile	YOY_AP020886	0.00	1.00	0.00	1.00	0.00	0.00	0.00	0.00	LAND	P												
Juvenile	YOY_AP020887	0.00	1.00	0.00	1.00	0.00	0.00	0.00	0.00	LAND	P												
Juvenile	YOY_AP020888	0.00	1.00	0.00	1.00	0.00	0.00	0.00	0.00	LAND	P												
Juvenile	YOY_AP020889	0.00	1.00	0.00	1.00	0.00	0.00	0.00	0.00	LAND	P												
Juvenile	YOY_AP020839	1.00	0.00	1.00	0.00	0.00	0.00	0.00	0.00	ANAD	P	BvAP018135	M	4/19/17	275	4.89E+01	1	BvAP017804	F	4/12/17	268	3.49E+01	1
Juvenile	YOY_AP020891	0.00	1.00	0.00	1.00	0.00	0.00	0.00	0.00	LAND	P												
Juvenile	YOY_AP020892	0.00	1.00	0.00	1.00	0.00	0.00	0.00	0.00	LAND	P												
Juvenile	YOY_AP020893	0.00	1.00	0.00	1.00	0.00	0.00	0.00	0.00	LAND	P												
Juvenile	YOY_AP020894	0.00	1.00	0.00	1.00	0.00	0.00	0.00	0.00	LAND	P												
Juvenile	YOY_AP020895	0.00	1.00	0.00	1.00	0.00	0.00	0.00	0.00	LAND	P												
Juvenile	YOY_AP020896	0.00	1.00	0.00	1.00	0.00	0.00	0.00	0.00	LAND	P												
Juvenile	YOY_AP020897	0.00	1.00	0.00	1.00	0.00	0.00	0.00	0.00	LAND	P												
Juvenile	YOY_AP020898	0.00	1.00	0.00	1.00	0.00	0.00	0.00	0.00	LAND	P												
Juvenile	YOY_AP020875	0.99	0.01	1.00	0.00	0.00	0.00	0.00	0.00	ANAD	P	BvAP019206	F	4/19/17	257	2.45E+01	1	BvAP018798	M	4/12/17	269	3.38E+01	1
Juvenile	YOY_AP020900	0.00	1.00	0.00	1.00	0.00	0.00	0.00	0.00	LAND	P												
Juvenile	YOY_AP020901	0.00	1.00	0.00	1.00	0.00	0.00	0.00	0.00	LAND	P												

ANAD - anadromous

LAND - landlocked

ANAD_BC - andromous backcross

LAND_BC - landlocked backcross

P - Pelagic

L - littoral
