# Supplementary material for: Restoration‐mediated secondary contact leads to introgression of alewife ecotypes separated by a colonial‐era dam
Source: Evol Appl. 2019 Nov 18;13(4):652–64. doi: 10.1111/eva.12890 (PMC7086056; doi:10.1111/eva.12890)
Supplement: Supplementary file 2 [file EVA-13-652-s002.pdf]

Supplementary Material

Restoration mediated secondary contact leads to introgression of alewife ecotypes separated by a colonial-era dam

Supplementary material I: Details of Microhaplotype development from ddRAD-seq data

Initially, we mined previously generated ddRAD-seq data from range-wide anadromous and landlocked alewife populations for sequences containing multiple SNPs and haplotypes (Baetscher, Hasselman, Reid, Palkovacs, & Garza, 2017). We also prepared an additional library with DNA from 12 anadromous Bride Brook and 12 landlocked Rogers Lake alewife. DNA was extracted, normalized and digested with the restriction enzymes *SbfI* and *EcoRI* following the protocol described by Peterson et al. (2012). Fragment sizes of 450 bp were selected using a Pippin Prep (Sage Science). The prepared library was run on a MiSeq instrument (Illumina Inc.) with a 2 x 300 bp paired-end sequencing protocol. Stacks v. 1.3.4 (Catchen, Hohenlohe, Bassham, Amores, & Cresko, 2013) was used for initial analysis with the following criteria -m 4, -M 4, -n 2 when assembling unique loci. Data from both of the ddRAD libraries were then filtered for loci containing 2 – 8 SNPs and with sequences in 60 % of samples. Loci were deemed suitable for retention if multiple haplotypes were observed and they were not obviously deviating from Hardy-Weinberg equilibrium (HWE), which we observed in the R package *microhaplot* (Ng, https://doi.org/10.5281/zenodo.820110). We then evaluated candidate loci with the Basic Local Alignment Search Tool (BLAST, Altschul, Gish, Miller, Myers, & Lipman, 1990) and the BLAST-Like Alignment Tool (BLAT, Kent, 2002) to identify and avoid duplicated or potentially paralogous loci. After coverage filtering of the two ddRAD-seq

libraries, 2,810 potential loci were identified in the previous ddRAD dataset and 1,407 identified from the new ddRAD dataset. We identified 231 unique loci with appropriate polymorphism that passed our initial quality control filters and for which primers could be designed using Batch Primer3 (You et al., 2008). These loci were then used to create the final amplicon sequencing panel of 114 polymorphic loci.

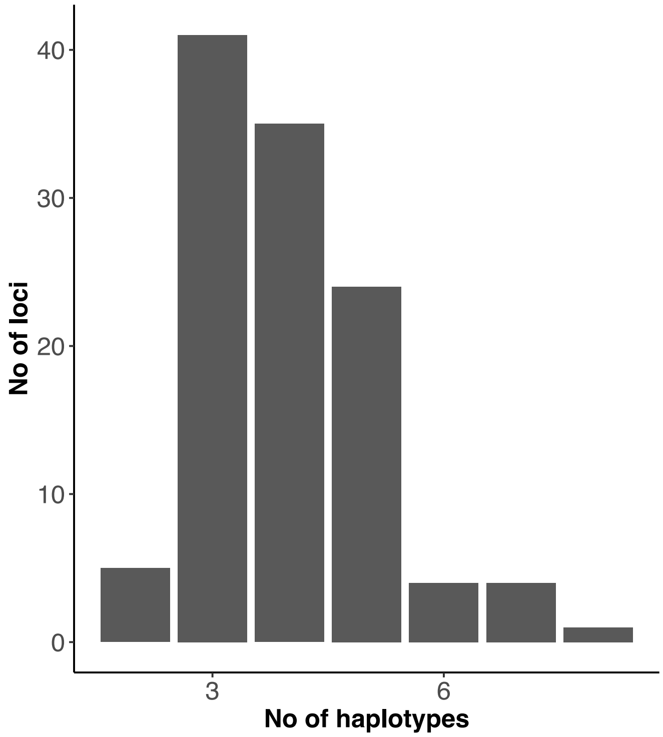

Figure S1 The distribution of the number of haplotypes of 114 microhaplotypes from 384 adult anadromous alewife. This represents all the haplotypes identified in the study.

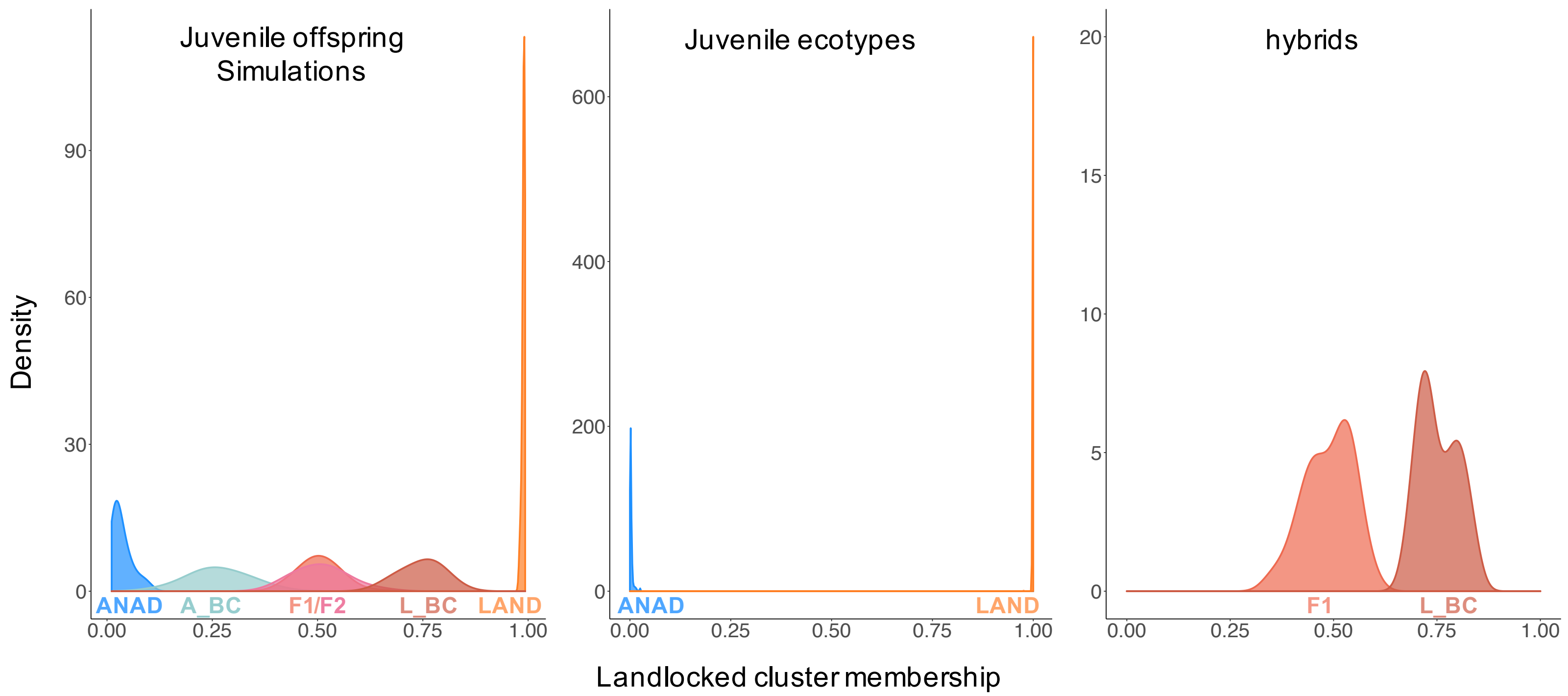

Figure S2 Bayesian posterior probability distributions for assignment of pure and hybrid classes to the landlocked group. Anadromous offspring are indicated in blue (ANAD), anadromous backcrosses (A_BC) in light blue, F1 hybrids in coral, F2 hybrids in violet, landlocked backcrosses (L_BC) in brown and pure landlocked (LAND) offspring in orange. A) Bayesian assignment of simulated individuals showing the expected distribution of q-values: F1 and F2 hybrids are expected to have entirely overlapping distributions. B) Bayesian assignment of anadromous and landlocked juveniles in 2017. C) Assignment of juveniles to hybrid classes in 2017. Anadromous backcrosses not shown, as there were only two individuals assigned to this group.

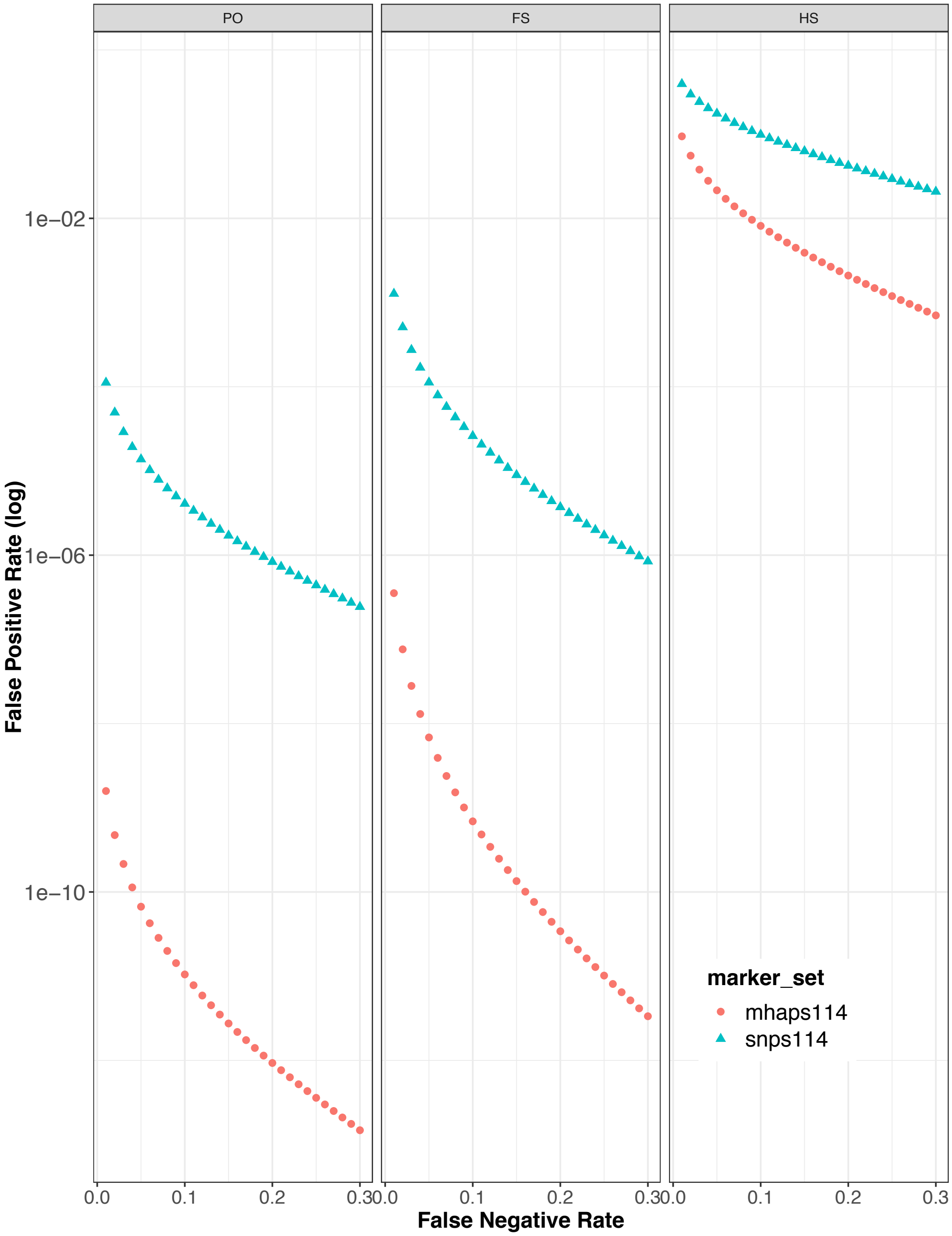

Figure S3 Comparison of power for kin identification with microhaplotypes and SNPs. Simulated false-positive rates for single parent-offspring pairs (PO), full-siblings (FS) and half-siblings (HS) at set false-negative rates with 114 microhaplotypes and for the single SNP in each locus with the highest heterozygosity (i.e., the most informative SNP).

References

Altschul, S.F., Gish, W., Miller, W., Myers, E.W., & Lipman, D.J. (1990). Basic local alignment search tool. *Journal of Molecular Biology*, *215*, 403–410.

Baetscher, D.S., Hasselman, D.J., Reid, K., Palkovacs, E.P., & Garza, J.C. (2017). Discovery and characterization of single nucleotide polymorphisms in two anadromous alosine fishes of conservation concern. *Ecology and Evolution*, *7*, 6638–6648.

Catchen, J., Hohenlohe, P.A., Bassham, S., Amores, A., & Cresko, W.A. (2013). Stacks: an analysis tool set for population genomics. *Molecular Ecology*, *22*, 3124–3140.

Kent, W.J. (2002). BLAT—the BLAST-like alignment tool. *Genome Research*, *12*, 656-664.

Peterson, B.K., Weber, J.N., Kay, E.H., Fisher, H.S., & Hoekstra, H.E. (2012). Double digest RADseq: an inexpensive method for de novo SNP discovery and genotyping in model and non-model species. *PloS One*, *7*, e37135.

You, F.M., Huo, N., Gu, Y.Q., Luo, M.C., Ma, Y., Hane, D., ... & Anderson, O.D. (2008). BatchPrimer3: a high throughput web application for PCR and sequencing primer design. *BMC Bioinformatics*, *9*, 253.
